# Tuning π-Acceptor/σ-Donor Ratio of the 2-Isocyanoazulene Ligand: Non-Fluorinated Rival of Pentafluorophenyl Isocyanide and Trifluorovinyl Isocyanide Discovered

**DOI:** 10.3390/molecules26040981

**Published:** 2021-02-12

**Authors:** Mason D. Hart, John J. Meyers, Zachary A. Wood, Toshinori Nakakita, Jason C. Applegate, Nathan R. Erickson, Nikolay N. Gerasimchuk, Mikhail V. Barybin

**Affiliations:** 1 Department of Chemistry, University of Kansas, 1567 Irving Hill Road, Lawrence, KS 66045, USA; Hart.mason.d@gmail.com (M.D.H.); woodz@usc.edu (Z.A.W.); toshinori.nakakita@yok.hamayaku.ac.jp (T.N.); jappleg2@emporia.edu (J.C.A.); nathan.erickson@csupueblo.edu (N.R.E.); 2 Department of Chemistry & Physics, Clayton State University, 2000 Clayton State Blvd., Morrow, GA 30260, USA; 3 Department of Chemistry, Missouri State University, 901 S. National Ave., Springfield, MO 65897, USA

**Keywords:** chromium pentacarbonyl, isocyanide, azulene, back-bonding, metal-to-ligand charge transfer, voltammetry

## Abstract

Isocyanoazulenes (CNAz) constitute a relatively new class of isocyanoarenes that offers rich structural and electronic diversification of the organic isocyanide ligand platform. This article considers a series of 2-isocyano-1,3-X_2_-azulene ligands (X = H, Me, CO_2_Et, Br, and CN) and the corresponding zero-valent complexes thereof, [(OC)_5_Cr(2-isocyano-1,3-X_2_-azulene)]. Air- and thermally stable, X-ray structurally characterized 2-isocyano-1,3-dimethylazulene may be viewed as a non-benzenoid aromatic congener of 2,6-dimethyphenyl isocyanide (2,6-xylyl isocyanide), a longtime “workhorse” aryl isocyanide ligand in coordination chemistry. Single crystal X-ray crystallographic {Cr–CNAz bond distances}, cyclic voltametric {*E_1/2_*(Cr^0/1+^)}, ^13^C NMR {δ(^13^CN), δ(^13^CO)}, UV-vis {dπ(Cr) → pπ*(CNAz) Metal-to-Ligand Charge Transfer}, and FTIR {ν_N__≡__C_, ν_C__≡__O_, k_C__≡__O_} analyses of the [(OC)_5_Cr(2-isocyano-1,3-X_2_-azulene)] complexes provided a multifaceted, quantitative assessment of the π-acceptor/σ-donor characteristics of the above five 2-isocyanoazulenes. In particular, the following inverse linear relationships were documented: δ(^13^CO_trans_) vs. δ(^13^CN), δ(^13^CO_cis_) vs. δ(^13^CN), and δ(^13^CO_trans_) vs. k_C__≡__O,trans_ force constant. Remarkably, the *net* electron withdrawing capability of the 2-isocyano-1,3-dicyanoazulene ligand rivals those of perfluorinated isocyanides CNC_6_F_5_ and CNC_2_F_3_.

## 1. Introduction

Organic isocyanides (C≡NR) are isolobal with carbon monoxide (C≡O) and offer attractive versatility as ligands in coordination chemistry from both steric and electronic standpoints [1,2,3,4,5,6,7]. Indeed, the steric encumbrance exerted by the substituent R is adjustable to a substantial extent [4,6,7]. In addition, modifying the electron withdrawing/donating properties of R affects the π-acceptor/σ-donor ratio (i.e., the *net* electron accepting or donating capability) of the CNR ligand [3]. The fundamental quest for matching or exceeding the π-acceptor/σ-donor ratio of CO using the isocyanide ligand platform spans more than three decades [3,4,6]. To date, only polyfluorinated isocyanides, such as the extremely unstable and hazardous CNCF_3_ [8,9], CNC_2_F_3_ [10,11], and CNC_6_F_5_ [12], have been shown to exhibit π-acceptor/σ-donor ratios comparable to or significantly approaching that of CO. In 2015, Figueroa and coworkers described convenient synthetic access to three exceptionally bulky fluorinated *m*-terphenyl isocyanides, which have reasonably good thermal and air stability [6]. Notably, CN(2,6-(3,5-(CF_3_)_2_C_6_H_3_)_2_-4-F-C_6_H_2_) (abbreviated as CN*p*-FAr^DArF2^) nearly rivals CNC_6_F_5_ in terms of the *net* relative electron accepting potential facilitated by M(dπ) → CNR(pπ*) back-bonding [4].

**Figure 1:**
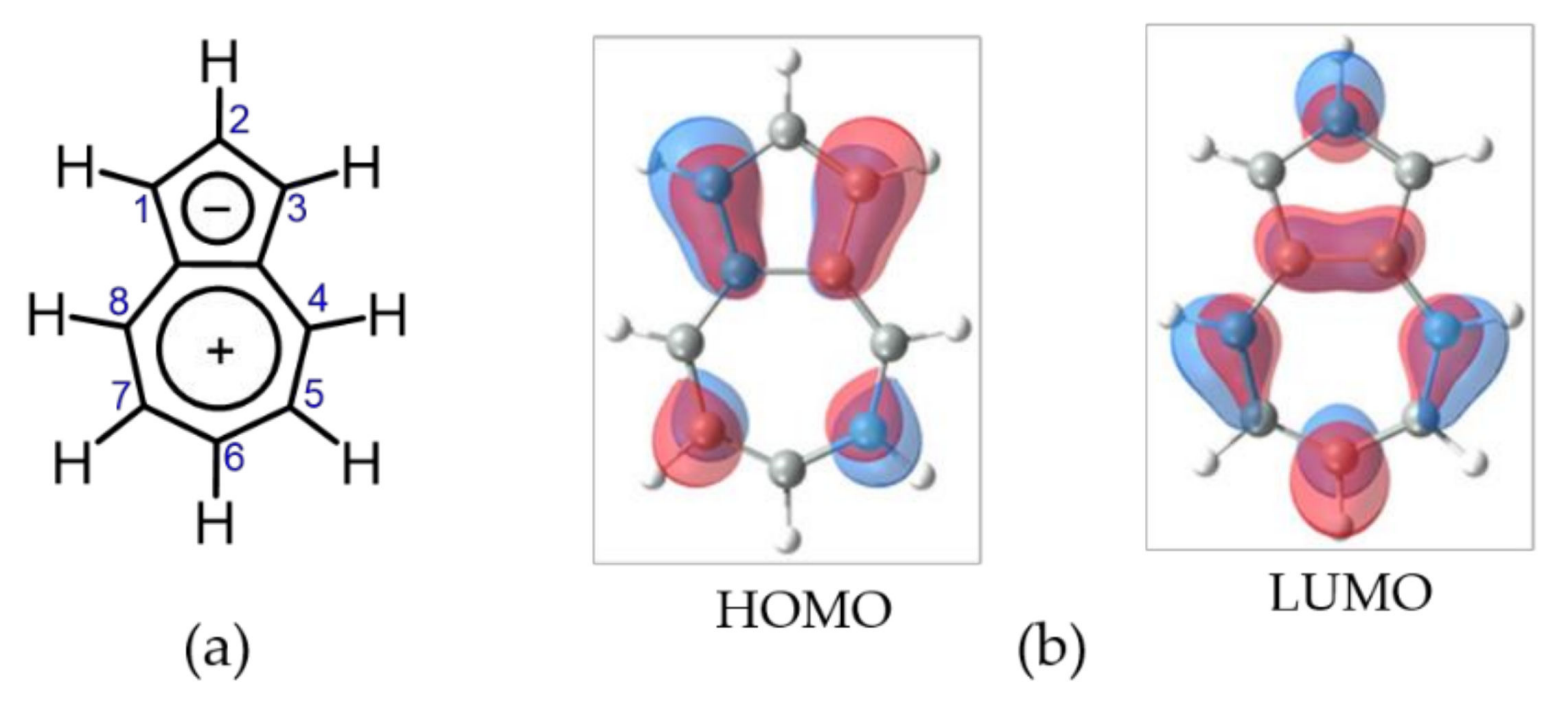
(**a**) Zwitterionic resonance form of azulene and the atom numbering scheme for the azulenic framework; (**b**) Highest Occupied Molecular Orbital (HOMO) and Lowest Unoccupied Molecular Orbital (LUMO) of azulene.

Among the currently known isocyanide ligands with a purely hydrocarbon substituent R, 4-isocyanoazulene and 6-isocyanoazulene have the highest π-acceptor/σ-donor ratios [13]. Azulene (C_10_H_8_) is a dark blue colored bicyclic aromatic hydrocarbon. Because of the uneven π-electron density distribution between its fused 5- and 7-membered *sp^2^* carbon rings, azulene has a dipole moment of 1.08 Debye (Figure 1a) [14]. Moreover, in contrast to benzenoid aromatic π-systems, this non-benzenoid isomer of naphthalene features complementary orbital density profiles within its Highest Occupied Molecular Orbital (HOMO) and Lowest Unoccupied Molecular Orbital (LUMO), as illustrated in Figure 1b [15].

Isocyanoazulenes (CNAz) form a distinct class of isocyanoarenes where the position of attachment of the isocyano functionality to the azulenic core (1, 2, 4, 5, or 6) has a profound effect on the ligands’ physicochemical properties, including their π-acceptor/σ-donor characteristics [13,16]. Advances in the coordination and surface chemistry of the 2-isocyanoazulene motif have yielded a quasi-molecular rectifier [17], an on-chip micro-supercapacitor [18], the first molecular π-linker asymmetrically anchored through both isocyano and mercapto junction groups [19], as well as various azulenic and biazulenic self-assembled monolayer films on metallic gold [19,20,21,22]. In addition, single molecules and nanostructures of 2-isocyano-1,3-di-*tert*-butoxycarbonylazulene adsorbed on Au(111), where the 2-isocyanoazulenic moieties are decoupled from the gold surface by bulky *tert*-butoxycarbonyl substituents, have been recently shown to be amenable to controlled manipulations by Scanning Tunneling Microscopy (STM) methods [23]. Redox-active complexes of ruthenium(II) tetraphenylporphyrin, featuring coordinated 2-isocyanoazulene [24], and liquid crystals based on the 2-isocyanoazulene complexes of gold(I) [25] have been reported as well.

**Figure 2:**
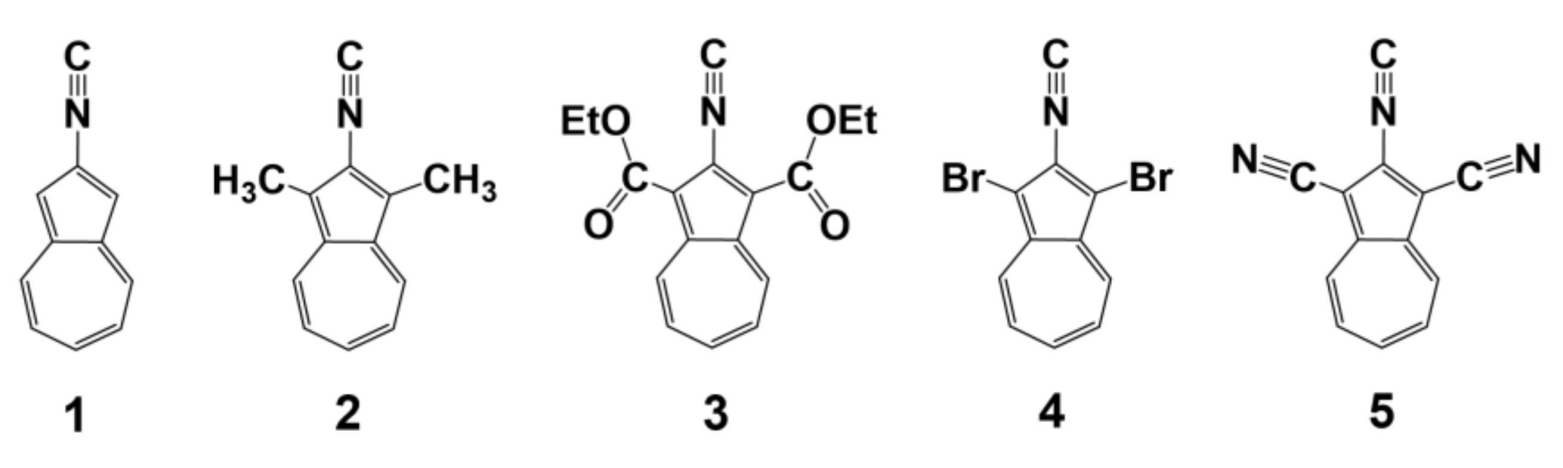
Five 2-isocyanoazulene ligands considered in this work: 2-isocyanoazulene (**1**), 2-isocyano-1,3-dimethylazulene (**2**), 2-isocyano-1,3-diethoxycarbonylazulene (**3**), 2-isocyano-1,3-dibromoazulene (**4**), and 2-isocyano-1,3-dicyanoazulene (**5**).

Herein, we consider the impact of the functionalization of carbon atoms 1 and 3 of the azulenic scaffold on the π-acceptor/σ-donor characteristics of the 2-isocyanoazulene ligand platform (compounds **1**–**5** in Figure 2). These properties were assessed through the comparative analyses of X-ray crystallographic; electrochemical; and ^13^C NMR, FTIR, and UV-vis spectroscopic data for the corresponding complexes [(OC)_5_Cr(CNAz)]. The parent compound, 2-isocyanoazulene (**1**), is an air- and thermally stable crystalline solid (mp: 70–73 °C) [13]. Whereas most relatively volatile isocyanides have a characteristic pungent, often disagreeable odor [26], **1** is nearly odorless (although some of us find that it exerts a very mild, minty scent). We demonstrate that 2-isocyano-1,3-dicyanoazulene (**5**) pushes the higher end limit of the achievable π-acceptor/σ-donor ratio of the isocyanoarene ligands without requiring fluorination. This limit has been hitherto bracketed by pentafluorophenyl isocyanide, CNC_6_F_5_ [12].

## 2. Results

### 2.1. Synthesis and Properties of 2-Isocyano-1,3-Dimethylazulene (2)

**Scheme 1:**
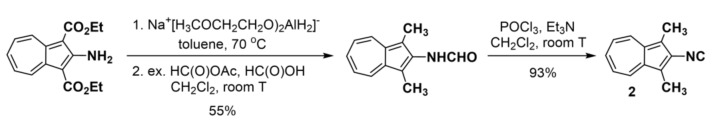
Synthesis of 2-isocyano-1,3-dimethylazulene (**2**).

Syntheses of the 2-isocyanoazulene ligands **1**, **3**, and **5** via formylation of the corresponding 2-aminoazulenes followed by dehydration of the resulting 2-formamidoazulenes were described in our previous reports [13,19,21]. In order to access 2-isocyano-1,3-dimethylazulene **2**, we envisioned converting both ester groups in 2-amino-1,3-diethoxycarbonylazulene into the methyl substituents via chemical reduction. Among various reductants considered, sodium bis(2-methoxyethoxy)aluminumhydride (Red-Al^®^) proved to be superior in effecting the reduction of the ester functionalities without compromising the integrity of the azulenic moiety. Formylation of the 2-amino-1,3-dimethylazulene intermediate (red oil) with acetic-formic anhydride afforded 2-formamido-1,3-dimethylazulene as a fluffy blue powder after workup. In a CDCl_3_ solution at 25 °C, this formamide exists as an unequal mixture of two conformational rotamers due to hindered internal rotation about the amide’s C-N bond. The ^1^H NMR resonances for the NHCHO unit of the more abundant *trans* H-C(O)-N(Az)-H rotamer show a characteristic ^3^*J*_HH_ coupling of 11 Hz [27]. Dehydration of 2-formamido-1,3-dimethylazulene afforded green microcrystals of **2** in a high yield (Scheme 1).

**Figure 3:**
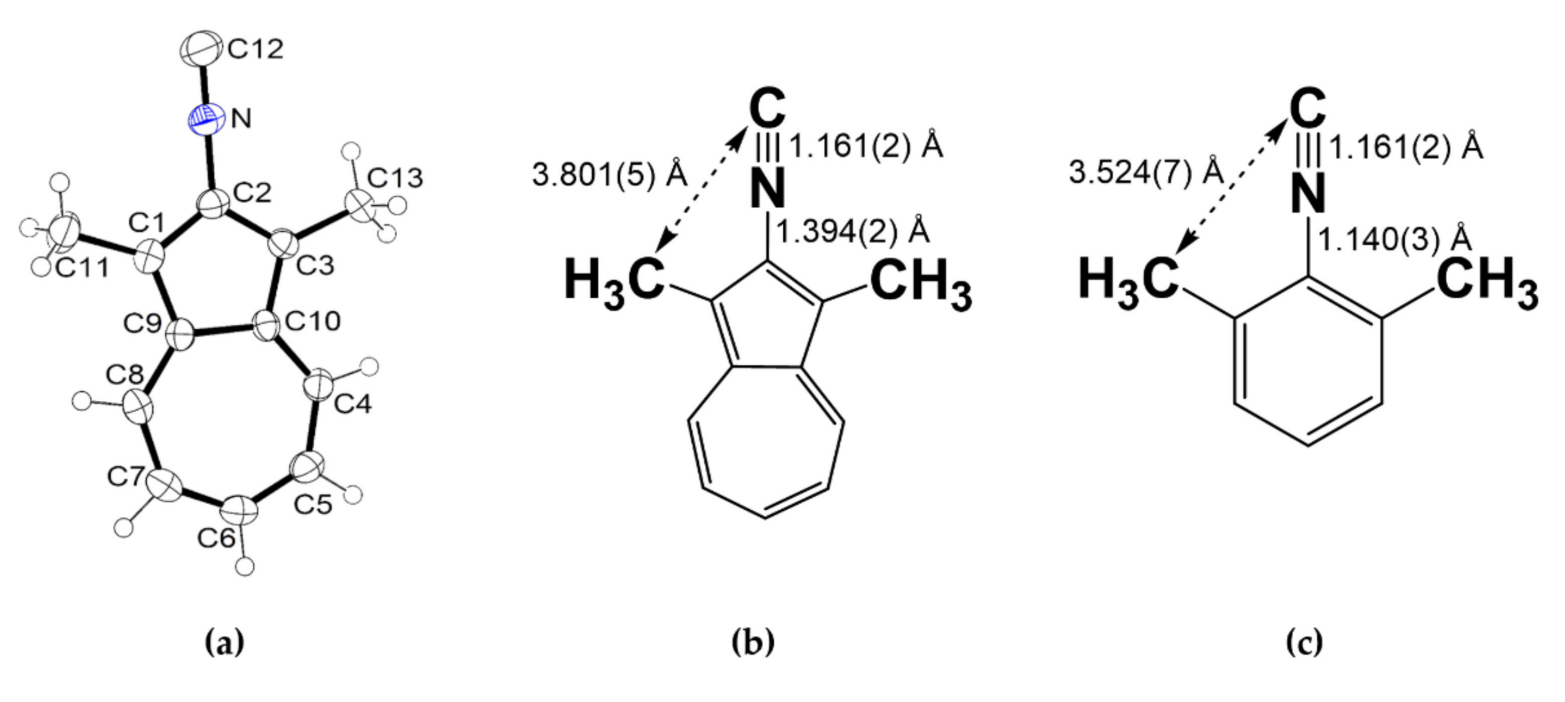
(**a**) Solid-state structure of **2**, 50% thermal ellipsoids; (**b**) C≡N, N–C bond lengths, and average C(methyl)⋅⋅⋅C(isocyano) distance in the solid-state structure of **2**; (**c**) C≡N, N–C bond lengths, and C(methyl)⋅⋅⋅C(isocyano) distance in the solid-state structure of 2,6-xylyl isocyanide (average parameters for two crystallographically independent molecules [30]).

Similar to **1**, **2** (m.p. 99–101 °C) is air- and thermally stable under ambient conditions and has only a very mild, inoffensive odor. These properties are in stark contrast with those of 2,6-dimethylphenyl isocyanide (2,6-CNXyl), which has been arguably the most commonly employed isocyanoarene in synthetic organic and organometallic chemistry for decades [28]. Indeed, 2,6-CNXyl (m.p. 73–74 °C) has a pungent odor, is air-sensitive, and must be stored at T < 5 °C to avoid extensive decomposition. The IR (ν_N≡C_), ^13^C NMR, and ^14^N NMR signatures of the isocyano group in **2** are comparable to the corresponding characteristics documented for **1** [13] and 2,6-CNXyl [29] (Table 1). Moreover, the X-ray structure of **2** revealed very close similarities between the metric parameters of the C≡N–C units in **2** and those in 2,6-CNXyl, previously reported by Schmidbaur et al. [30] (Figure 3). As expected from the geometric considerations, the steric encumbrance exerted by the methyl substituents with respect to the isocyano unit is only slightly less pronounced in **2** than in 2,6-CNXyl (Figure 3b,c). Notably, the *ortho* protection of the isocyano functionality with hydrocarbon substituents is often critical for the stability of highly electron-rich transition metal–benzenoid isocyanoarene complexes, especially isocyanometalates [3].

**Figure 4:**
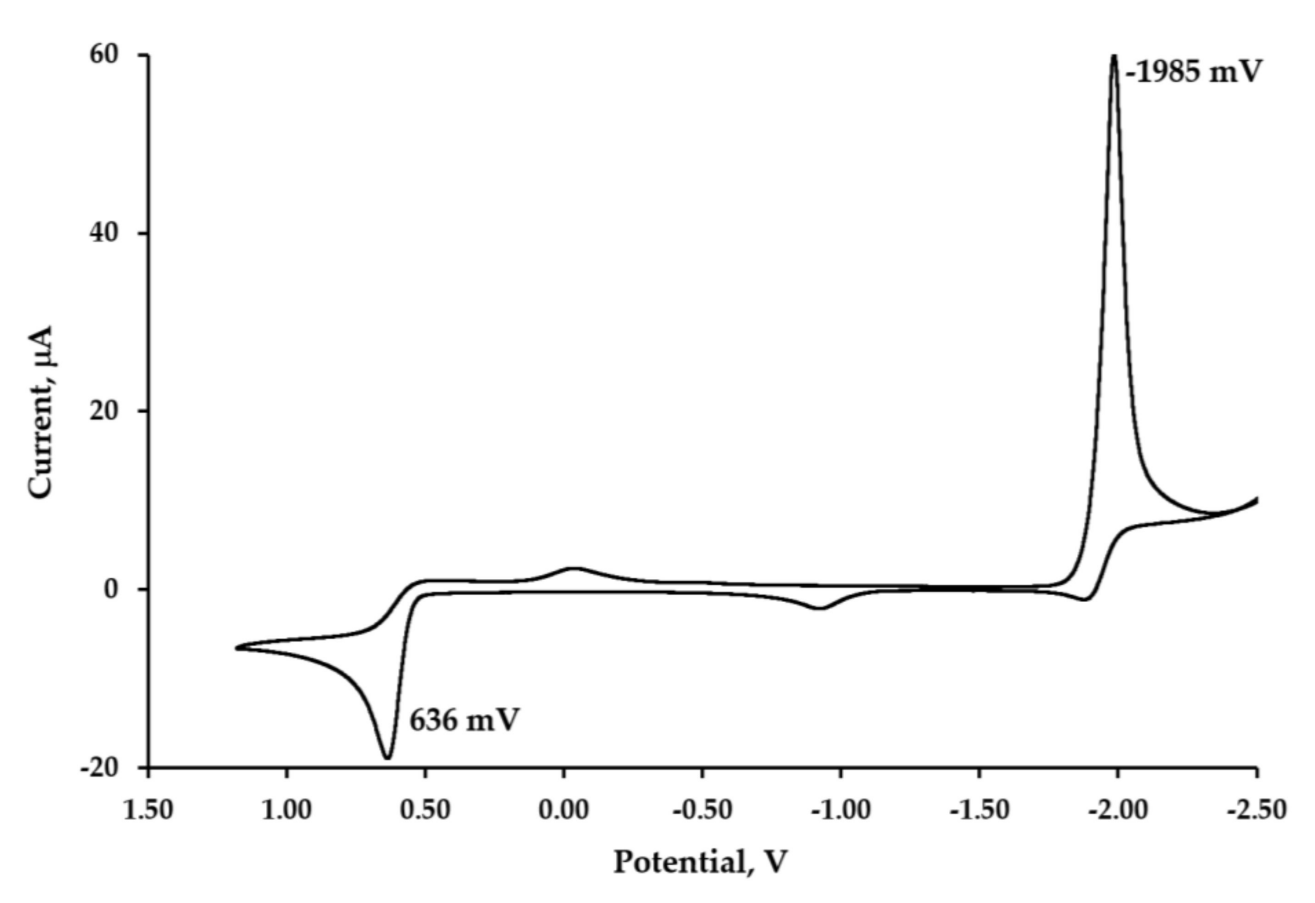
Cyclic voltammogram of ca. 0.02 M solution of **2** in 0.1 M [^n^Bu_4_N][PF_6_]/CH_2_Cl_2_ vs. external Cp_2_Fe/Cp_2_Fe^+^ at 25 °C. Scan rate = 100 mV/s.

The cyclic voltammetry profile of **2** in CH_2_Cl_2_/[^n^Bu_4_N][PF_6_] shows the redox tolerance span of ca. 2.6 V (Figure 4) between the oxidation and reduction waves. The reduction of **2** is accompanied by an adsorption process as evidenced by the characteristic shape of the cathodic wave. Electrochemical reduction and oxidation of monoazulenic derivatives are usually irreversible due to dimerization of the resulting radical-anion or radical-cation, respectively [31]. Following Mikkelsen’s interpretation [31] of the qualitatively similar cyclic voltammetry profiles of other azulenic derivatives, the anodic current at −0.92 V in Figure 4 likely signifies the oxidation of the biazulenic dianion, whereas the cathodic current at −0.04 V corresponds to the reduction of the biazulenic dication. For **2**, the oxidation potential is 0.28 V less positive, and the reduction potential is 0.13 V more negative compared to the corresponding values documented for **1**, which is consistent with the electron-releasing nature of the methyl substituents in **2**. The smaller effect of 1,3-methylation of the 2-isocyanoazulene scaffold on its reduction (vs. oxidation) potential reflects the lack of orbital density at the 1,3-carbon atoms in the LUMO of **1** [15]. In general, incorporation of an electron donating substituent at an odd-numbered position of the azulenic framework results in raising the energy of its HOMO while much less significantly affecting the energy of its LUMO (Figure 1b) [15,16].

### 2.2. Preparation of Complexes [(OC)_5_Cr(2-Isocyano-1,3-X_2_-Azulene)], X = H, CH_3_, CO_2_Et, Br, and CN

**Scheme 2:**
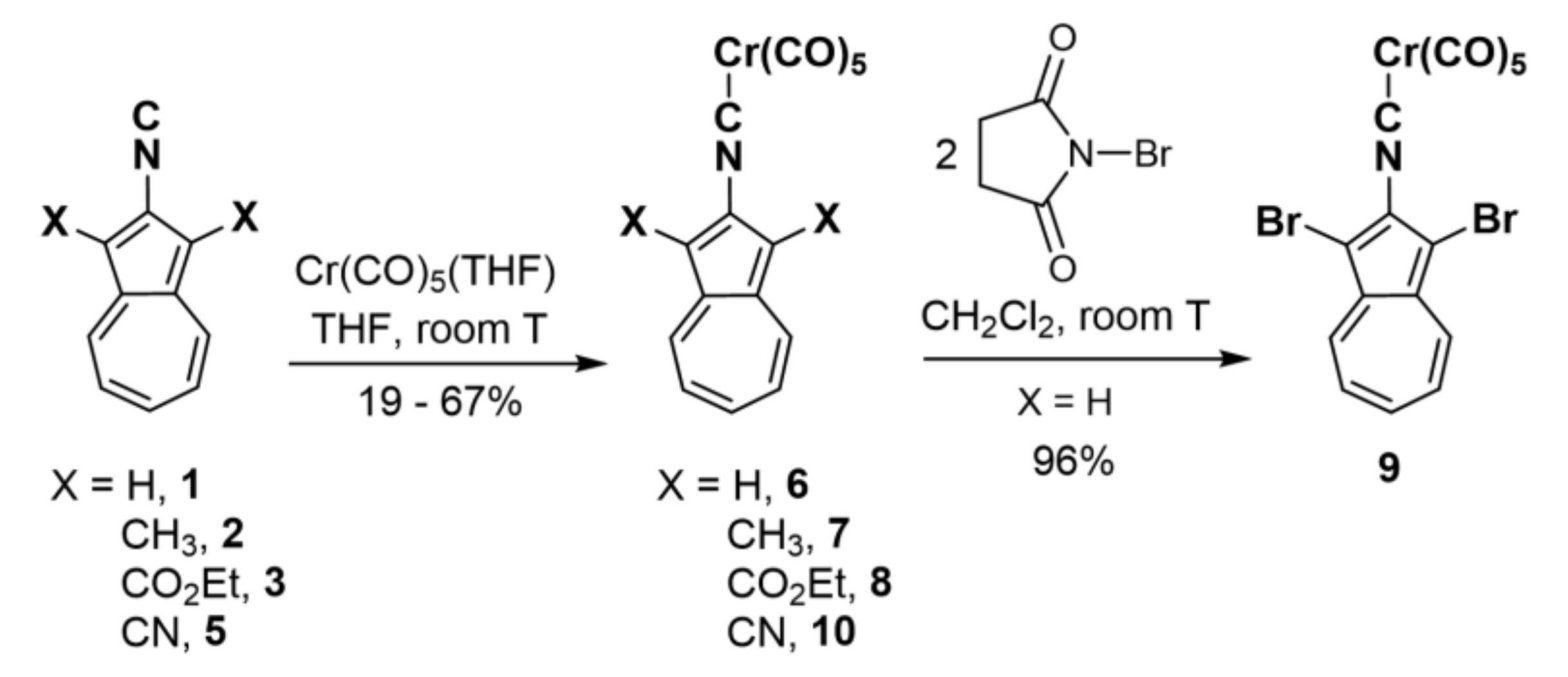
Syntheses of (OC)_5_Cr(CNAz) complexes **6**–**10**.

Combining an orange solution of [Cr(CO)_5_(THF)], generated in situ by UV-photolysis of Cr(CO)_6_ in THF, with 2-isocyanoazulene ligands **1**, **2**, **3**, or **5**, afforded the corresponding deeply colored, air- and thermally stable complexes [Cr(CO)_5_(CNAz)] complexes **6**, **7**, **8**, and 1**0** (Scheme 2). Treatment of [(OC)_5_Cr(2-isocyanoazulene)] (**6**) with two equivalents of N-bromosuccinimide resulted in bromination of the carbon–atom positions 1 and 3 of the coordinated 2-isocyanoazulene to give, nearly quantitatively, complex **9**, which features the 2-isocyanoazulene ligand **4** bound to the Cr(CO)_5_ unit (Scheme 2). This bromination reaction represents a relatively rare example of selectively modifying a C≡NR ligand’s substituent R without affecting the structural transformation/integrity of the rest of the complex [10,11,19].

### 2.3. X-Ray Crystallographic Analysis of 6, 7, 8, and 10

**Figure 5:**
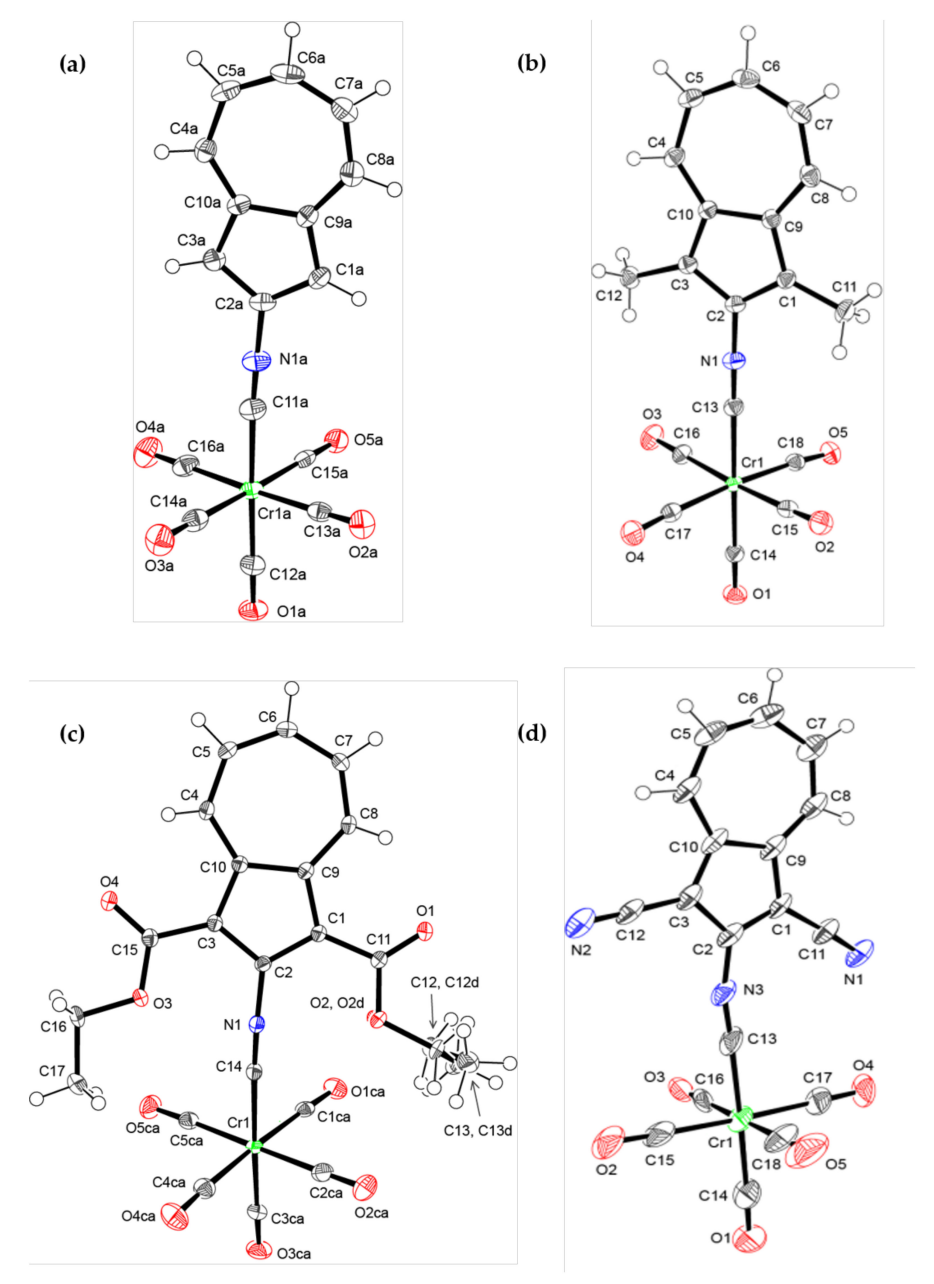
(**a**) One of two crystallographically independent molecules in the solid-state structure of **6** (the other molecule is shown in Figure S5); (**b**) the solid-state structure of **7**; (**c**) the solid-state structure of **8** (one ethoxy group shows a minor disorder over two positions; also see Figure S6); (**d**) the solid-state structure of **10**. All thermal ellipsoids are drawn at the 50% probability level.

Single crystals of **6**, **7**, **8**, and **10** suitable for X-ray diffraction studies were grown by diffusion of pentane layered over a CH_2_Cl_2_ solution of **6**, **7**, or **10** and by slow evaporation of the solvent from a CH_2_Cl_2_ solution of **8**. The molecular structures of complexes **6**, **7**, **8**, and **10** determined by single crystal X-ray crystallography are illustrated in Figure 5. For **6**, one of two crystallographically independent molecules is shown (Figure 5a). One of the ethyl ester groups in the X-ray structure of **8** exhibits a minor positional disorder over two occupancies (Figure 5c and Figure S6). The pertinent metric data for the above new [(OC)_5_Cr(CNR)] species, along with those for [(OC)_5_Cr(CN^t^Bu)] [32], [(OC)_5_Cr(CNC_2_F_3_)] [10], and [(OC)_5_Cr(CN*p*-FAr^DArF2^)] [6], are collated in Table 2. For **6**, **7**, **8**, and **10**, the Cr–CNR bond length, which reflects the π-acceptor/σ-donor ability of the CNR ligand (particularly, the extent of π back-bonding interaction between the chromium(0) center and the isocyanide) [33], is significantly shorter than that in [(OC)_5_Cr(CN^t^Bu)]. Moreover, the Cr–CNR bond distance of 1.937(2) Å in **10** is 0.03 Å shorter than the corresponding Cr–C bond length in [(OC)_5_Cr(CN*p*-FAr^DArF2^)] and even slightly shorter (albeit at the edge of the 3σ criterion margin) than the Cr–CNR bond distance of 1.942(2) Å documented for perfluorinated [(OC)_5_Cr(CNC_2_F_3_)] (Table 2). While **10** and [(OC)_5_Cr(CNC_2_F_3_)] feature the shortest Cr–CNR bonds among the compounds listed in Table 2, these complexes have the longest Cr–CO_trans_ bonds, which is consistent with the CNR and CO_trans_ ligands being in direct competition with each other for Cr(dπ) → L(pπ*) back-bonding. In addition, the data in Table 2 show that **10** has the longest C–NR distance and the most bent C–N–C angle (164.8(3)°). Both of these features can be viewed as hallmarks of more pronounced Cr(dπ) → CNR(pπ*) back-bonding, exerting partial rehybridization of the N-atom toward the *sp^2^* configuration [3,4]. However, for a metal–isocyanide complex, deviation of the C-N-C angles from linearity must be taken cum grano salis because the magnitude of such deviation can be affected by steric constraints in the vicinity of the coordinated isocyano junction and/or by crystal packing forces (*cf*. two crystallographically unique molecules of **6**: Table 2; Figure 5a, Figures S4 and S5).

### 2.4. Electronic Absorption, Infrared, and ^13^C NMR Spectroscopic Studies of 6, 7, 8, 9 and 10

**Figure 6:**
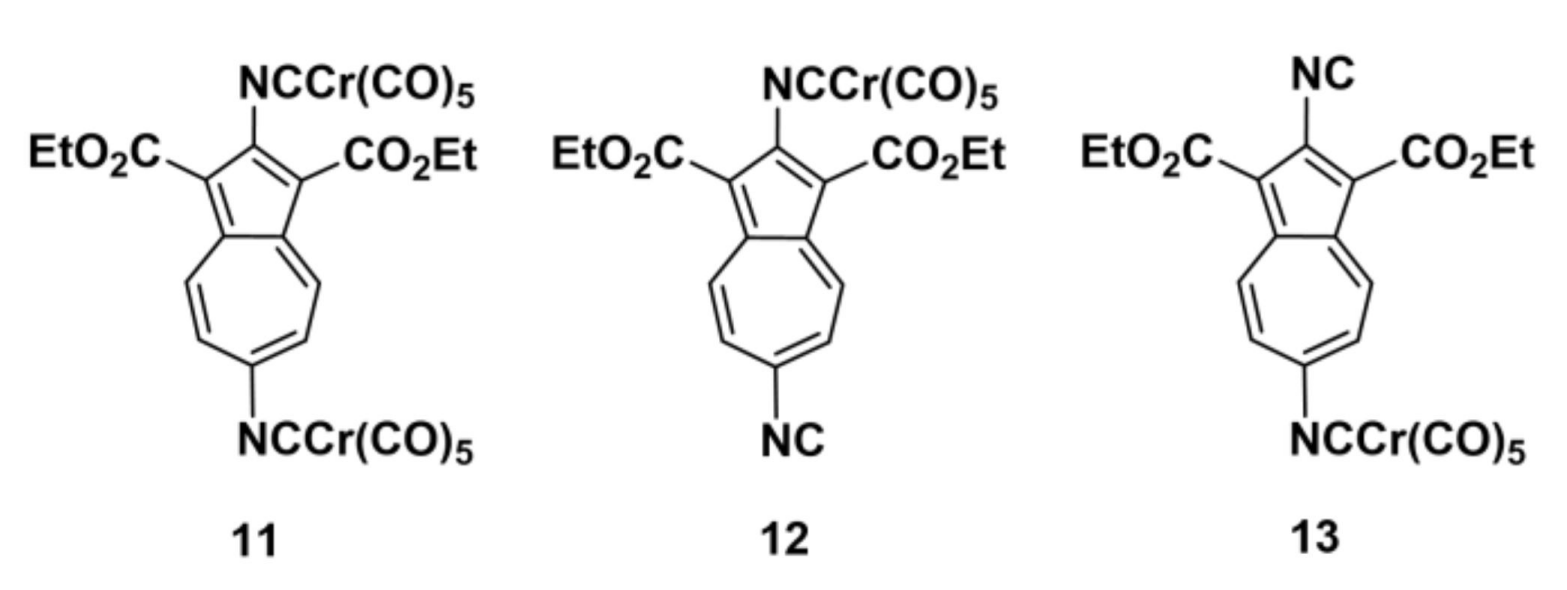
Di- and mononuclear complexes of 2,6-diisocyano-1,3-diethoxycarbonylazulene with [Cr(CO)_5_] [34].

**Figure 7:**
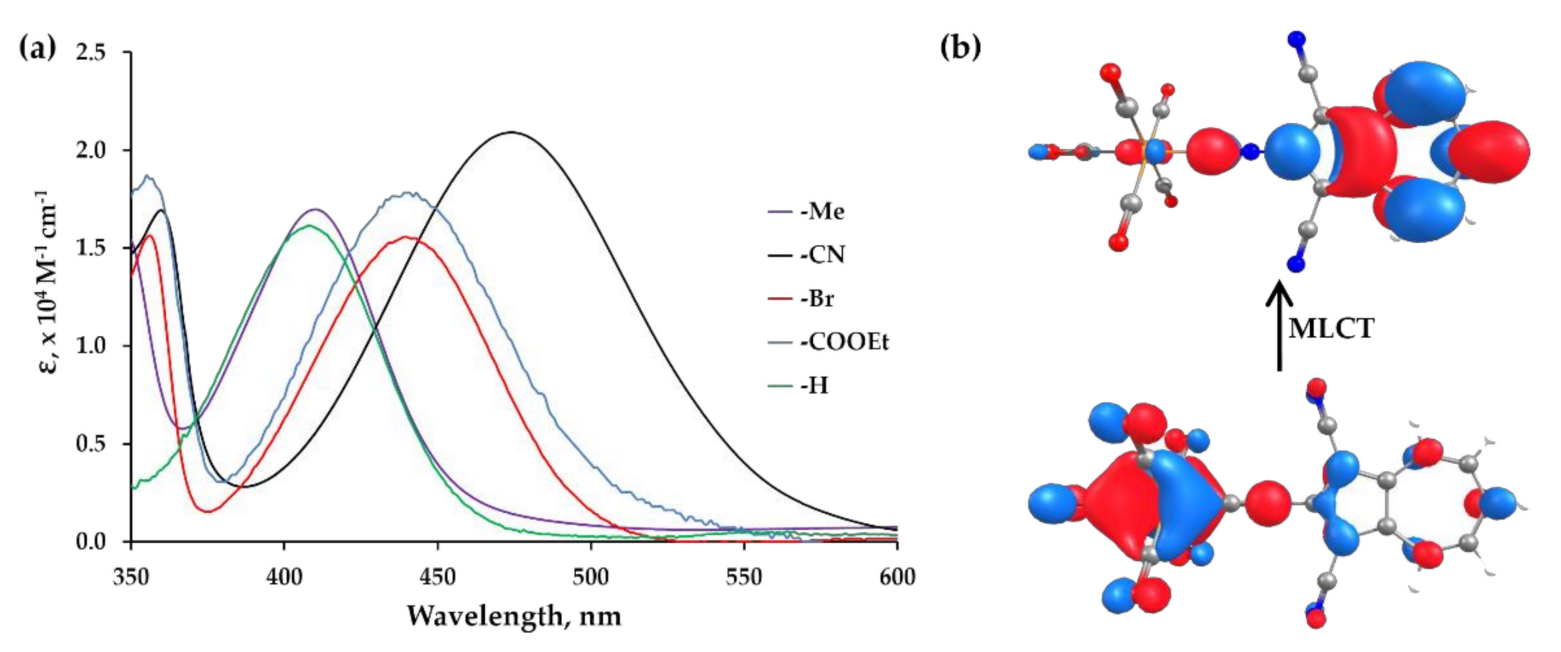
(**a**) Electronic absorption spectra of **6** (green), **7** (purple), **8** (blue), **9** (red), and **10** (black) in CH_2_Cl_2_; (**b**) DFT-calculated molecular orbitals involved in Cr(dπ) → CNAz(pπ*) MLCT for **10**.

In our previous report [34], we showed via Time-Dependent Density Functional Theory (TDDFT) analysis that the highly intense bands at 522 nm in the electronic absorption spectrum of dinuclear complex [{(OC)_5_Cr}_2_(μ-2,6-diisocyano-1,3-diethoxycarbonylazulene)] (**11**, Figure 6) and at ca. 480 nm in the electronic absorption spectra of isomeric mononuclear complexes [(OC)_5_Cr(2,6-diisocyano-1,3-diethoxycarbonylazulene)] (**12** and **13**, Figure 6) in CH_2_Cl_2_ solutions arise from Cr(dπ) → 2,6-diisocyano-1,3-diethoxycarbonylazulene(pπ*) charge transfer excitations. By analogy, we assign the prominent bands at 409, 410, 441, 440, and 474 nm in the electronic absorption spectra (visible region) of CH_2_Cl_2_ solutions of **6**, **7**, **8**, **9** and **10**, respectively, to Cr(dπ) → CNAz(pπ*) Metal-to-Ligand Charge Transfer (MLCT) (Figure 7a). For example, the molecular orbitals involved in such MLCT in **10** are illustrated in Figure 7b. The energy of this MLCT transition decreases in the order of increasing the electron withdrawing character of the substituents at the 1,3-positions of the 2-isocyanoazulene ligand, i.e., H (**6**) ≈ CH_3_ (**7)** > CO_2_Et (**8**) ≈ Br (**9**) > CN (**10**) (Table 3). The MLCT energy depresses further upon incorporating an electron withdrawing substituent at an even-numbered position of the coordinated 2-isocyanoazulene ligand. For instance, a 40 nm red shift of the Cr(dπ) → CNAz(pπ*) MLCT band occurs when the H-atom at position 6 of the azulenic scaffold in **8** is replaced with an isocyano substituent (complex **12**).

Coordination of an isocyanide ligand to the Cr(CO)_5_ motif exerts two mutually opposing effects on the vibrational force constant k_N≡C_ and, hence, on the υ_N≡C_ stretching frequency [4,6]. The σ-donation of the lone pair on the isocyanide’s carbon atom to the metal center strengthens the C≡N bond, because the molecular orbital involving this lone pair is antibonding with respect to the C≡N bond in the free isocyanide ligand. On the other hand, the M(dπ) → CNR(pπ*) back-bonding interaction weakens the C≡N bond. The υ_N≡C_ bands at 2127, 2115, 2127, 2121, and 2114 cm^−1^ documented for the free isocyanides **1**, **2**, **3**, **4**, and **5**, respectively, shift to higher energy by 11, 14, 13, 11, and 6 cm^−1^ upon formation of the corresponding complexes **6**, **7**, **8**, **9**, and **10** (Table 4). While the σ-bonding chromium-isocyanide interaction overpowers π-back-bonding in terms of its influence on υ_N≡C_, the relatively small blue shift in υ_N≡C_ accompanying the complexation of **5** to form **10** indicates a substantially higher π-acceptor/σ-donor ratio of **5** compared to those of **1**, **2**, **3**, and **4**.

**Figure 8:**
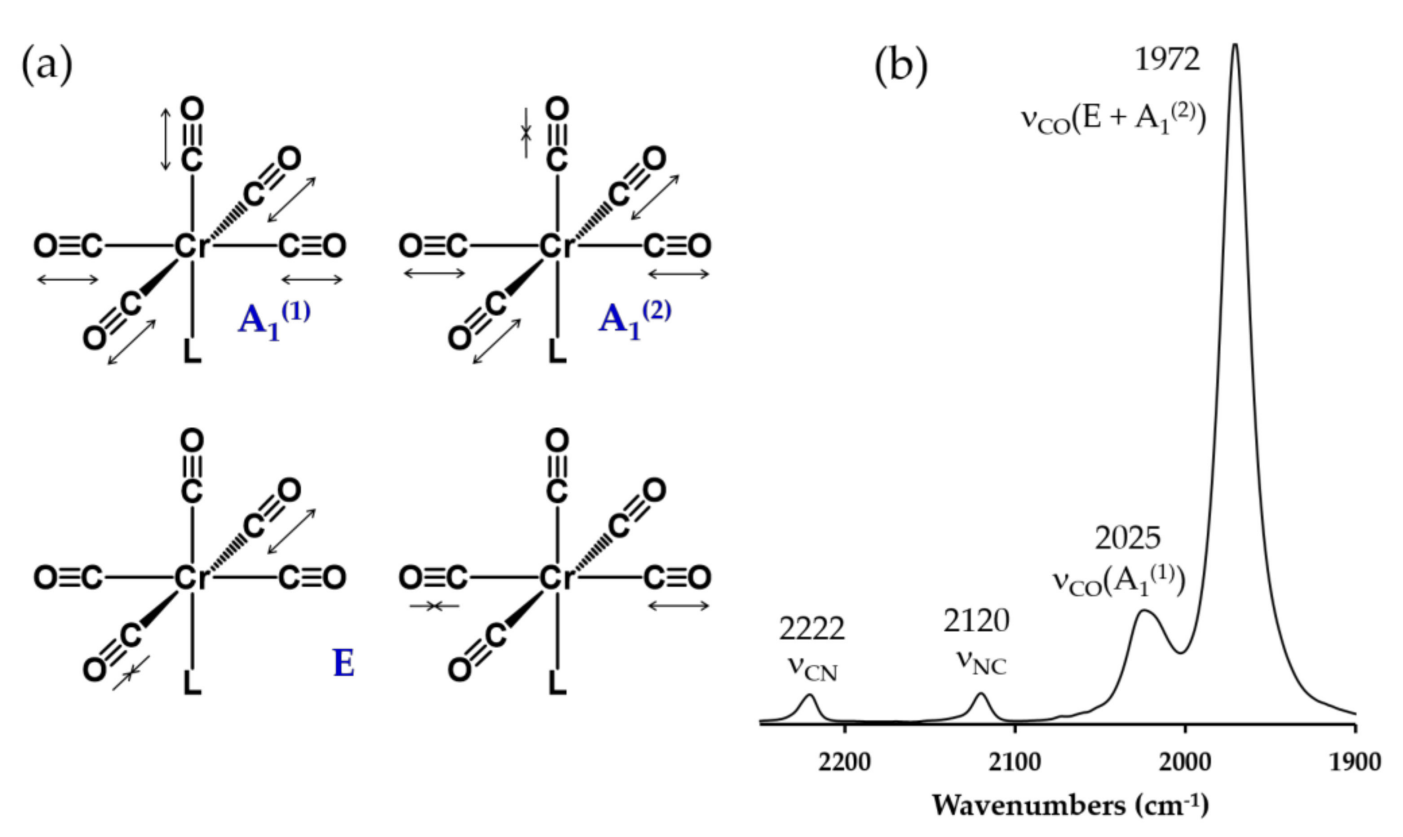
(**a**) IR-active υ_C≡O_ vibrational modes for [M(CO)_5_L]; (**b**) FTIR spectrum of **10** in CH_2_Cl_2_.

**Figure 9:**
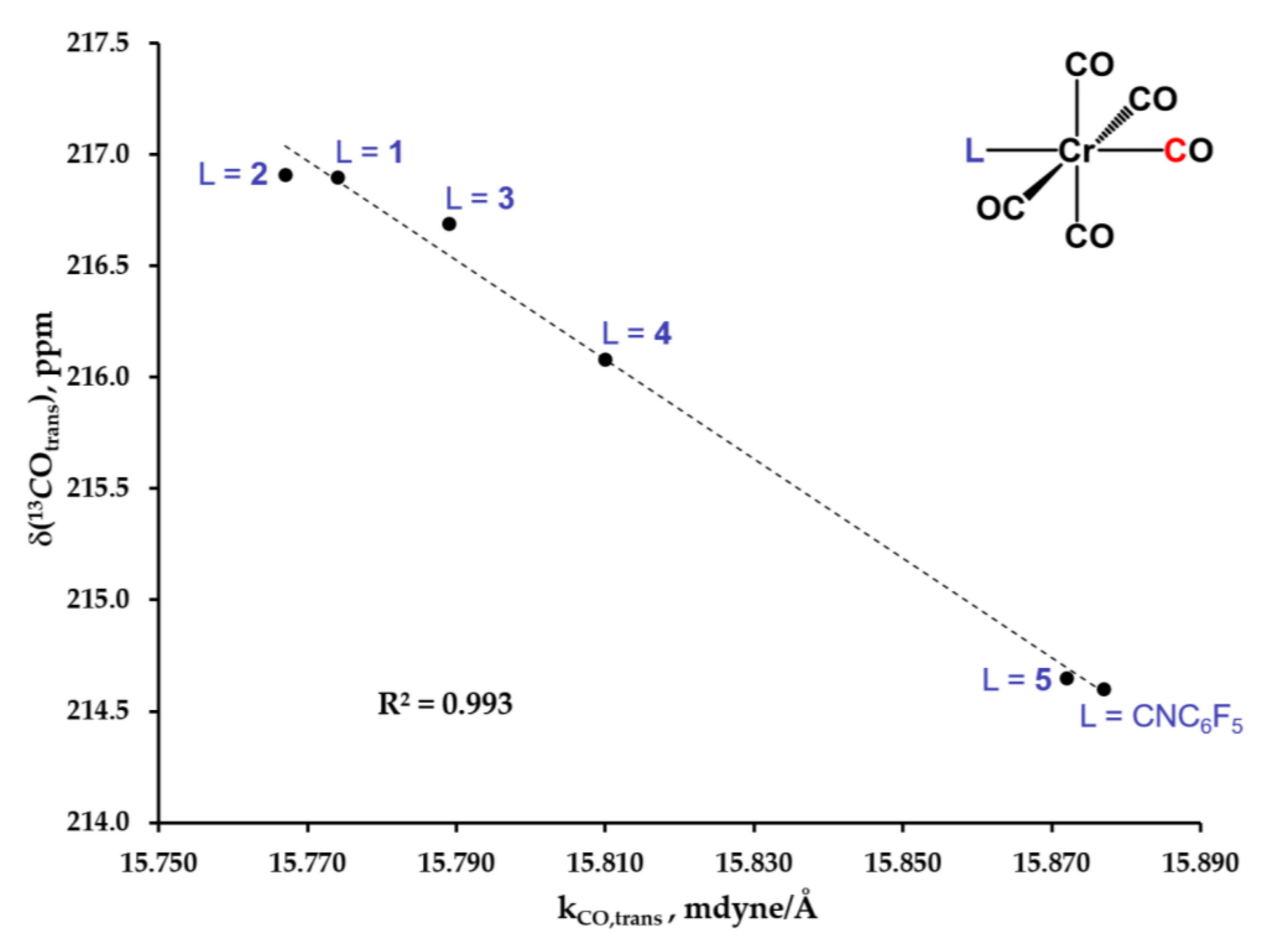
Plot of ^13^C NMR chemical shifts δ(^13^CO_trans_) vs. k_CO,trans_ for the series of complexes **6**, **7**, **8**, **9**, **10**, and [(OC)_5_Cr(CNC_6_F_5_)] [12]. All ^13^C NMR data were recorded for solutions in CDCl_3_. The IR spectra of **6**, **7**, **8**, **9**, and **10** were recorded for solutions in CH_2_Cl_2_, and the IR spectrum of [(OC)_5_Cr(CNC_6_F_5_)] was obtained for a solution in pentane.

The FTIR spectra of **6**–**10** revealed typical υ_C≡O_ absorption profiles for the nearly C_4_v-symmetric [M(CO)_5_L] complexes (Table 4). As in the case of many other [M(CO)_5_(CNR)] complexes, the υ_C≡O_(A_1_^(2)^) band in the FTIR spectra of **6**–**10** is completely obscured by the very intense υ_C≡O_(E) band (e.g., Figure 8). This band overlap inherently limits precision in determining the value of υ_C≡O_(A_1_^(2)^). The π-acceptor/σ-donor characteristics of the isocyanide ligand in [(OC)_5_Cr(CNR)] affect electron richness of the Cr center, which, in turn, is reflected in the magnitudes of the carbonyl vibrational force constants k_C__≡O,trans_ and k_C≡O,cis._ The approximate values of these force constants can be deduced from the υ_C≡O_ data by applying the Cotton–Kraihanzel approximation [35] or the newer variation thereof developed by Karakaş and Kaya [36]. We employed the latter approach to calculate the k_C≡O,trans_ values for **6**–**10**, as well as for [(OC)_5_Cr(CNC_6_F_5_)], from the corresponding υ_C≡O_(A_1_^(1)^) and υ_C≡O_(A_1_^(2)^) experimental data. As shown in the right column of Table 4, the magnitude of k_C≡O,trans_ increases in the order **7** < **6** < **8** < **9** < **10** ≈ [(OC)_5_Cr(CNC_6_F_5_)]. Notably, there is a clear inverse-linear correlation between the ^13^C NMR chemical shifts δ and the corresponding vibrational force constants k_C≡O,trans_ for the above series of complexes (Figure 9). Thus, the π-acceptor/σ-donor ratio of the 2-isocyano-1,3-dicyanoazulene ligand **5** is comparable to that of CNC_6_F_5_.

**Figure 10:**
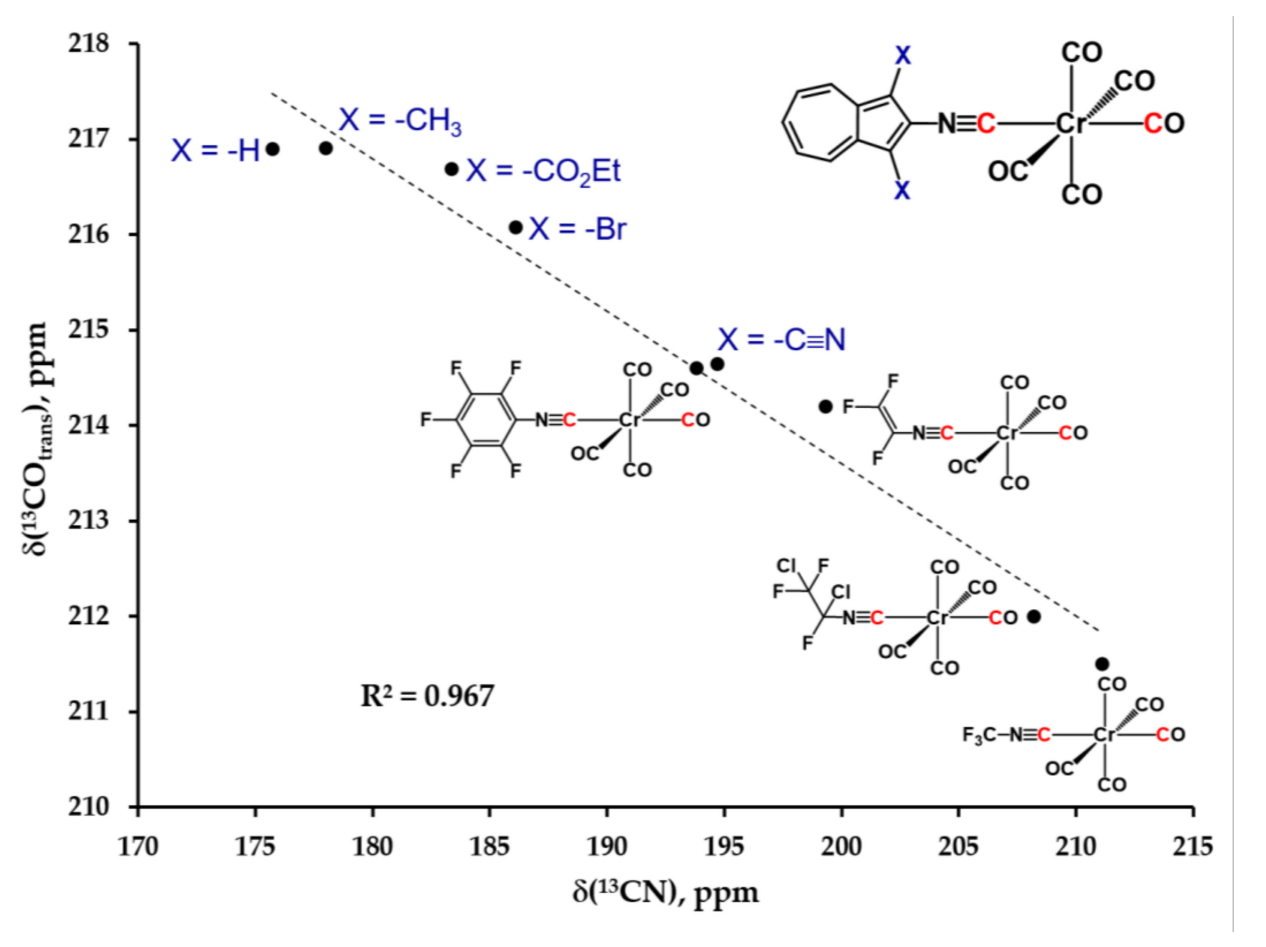
Plot of ^13^C NMR chemical shifts δ(^13^CO_trans_) vs. δ(^13^CN) for the series of [(OC)_5_Cr(CNR)] complexes listed in Table 5. All ^13^C NMR data were collected for solutions in CDCl_3_.

Recently, we demonstrated the utility of ^13^C NMR δ(^13^CO_trans_) or δ(^13^CO_cis_) vs. δ(^13^CN) inverse-linear correlations in assessing the π-acceptor/σ-donor capabilities of 6-substituted 2-isocyano-1,3-diethoxycarbonylazulene ligands in [(OC)_5_Cr(CNAz)] complexes [19]. Similarly, Table 5 compiles the ^13^C NMR data documented for the [(OC)_5_Cr(CN)] cores of **6**–**10** as well a few related complexes containing polyfluorinated isocyanide ligands. Figure 10 features a graphical representation of the δ(^13^CO_trans_) vs. δ(^13^CN) data, whereas the analogous δ(^13^CO_cis_) vs. δ(^13^CN) plot is provided in Figure S7. The inverse-linear correlations revealed in Figure 10 and Figure S7 underscore the electronic tunability of the 2-isocyanoazulene ligand platform through 1,3-subsitution of the azulenic moiety and further confirm that the π-acceptor/σ-donor ratio of the non-fluorinated isocyanide ligand **5** rivals those of perfluorinated CNC_6_F_5_ and CNC_2_F_3_.

### 2.5. Electrochemical Studies

**Figure 11:**
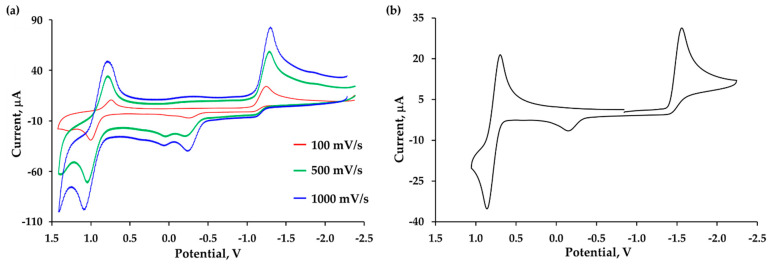
(**a**) Variable scan rate (100, 500, and 1000 mV/s) cyclic voltammograms for ca. 0.02 M solution of **10** in CH_2_Cl_2_ with 0.1 M [^n^BuN][PF_6_]; (**b**) cyclic voltammogram of ca. 0.02 M solution of **8** in CH_2_Cl_2_ with 0.1 M [^n^BuN][PF_6_]. Scan rate = 100 mV/s.

Complexes **6**, **7**, **8**, and **10** undergo an irreversible, azulene-centered reduction with the *E*_p,c_ values (vs. Cp_2_Fe/Cp_2_Fe^+^) spanning 0.68 V and becoming less negative in the order of the decreasing net electron releasing character of the substituents X at positions 1 and 3 of the azulenic moiety: −2.06 V (**7**, X = CH_3_), −1.86 V (**6**, X = H), −1.56 V (**8**, X = CO_2_Et), −1.38 V (**10**, X = CN); scan rate = 100 mV/s. The variable scan rate set of cyclic voltammograms for **10** dissolved in CH_2_Cl_2_ containing [^n^Bu_4_N][PF_6_] electrolyte is illustrated in Figure 11a. At the scan rate of 1000 mV/s, **10** undergoes Cr-centered oxidation at the half-wave potential *E*_1/2_(Cr^0/+^) = 946 mV (i_c_/i_a_ = 0.8) vs. Cp_2_Fe/Cp_2_Fe^+^. In comparison, the Cr^0^ → Cr^+^ oxidations of [(OC)_5_Cr(2,4,6-CNC_6_H_2_Cl_3_)] and [(OC)_5_Cr(4-CNC_6_H_4_CF_3_)] occur at *E*_1/2_ = 771 mV (i_c_/i_a_ = 0.92) and *E*_1/2_ = 706 mV (i_c_/i_a_ = 0.91), respectively, as documented by Johnston at the same scan rate of 1000 mV/s in CH_3_CN/[^n^Bu_4_N]BF_4_] [37]. This indicates that the π-acceptor/σ-donor ratio of 2-isocyano-1,3-dicyanoazulene (**5**) is substantially higher than those of 2,4,6-trichlorophenyl isocyanide and 4-trifluoromethylphenyl isocyanide. In fact, the half-wave redox potential of [(OC)_5_Cr(4-CNC_6_H_4_CF_3_)] nearly matches that of **8** {*E*_1/2_(Cr^0/+^) = 777 mV, i_c_/i_a_ = 0.83 at 100 mV/s scan rate, Figure 11b}. Thus, 2-isocyano-1,3-diethoxycarbonylazulene (**3**) is similar to 2,4,6-trichlorophenyl isocyanide in terms of its electron donating/withdrawing characteristics as ligand. For **6** and **7**, the Cr^0^ → Cr^+^ oxidation is at least partially masked by irreversible oxidation of the azulenic moieties in these complexes (Figures S8 and S9).

## 3. Conclusions

In this work, we systematically considered the structural, spectroscopic (UV-vis, ^13^C NMR, and FTIR), as well as electrochemical properties of the [(OC)_5_Cr(CNAz)] complexes containing 1,3-substituted 2-isocyanoazulene ligands. While 2-isocyanoazulenes **1** and **2** may be viewed as thermally and air stable, nearly odorless congeners of the malodorous and air- and thermally sensitive phenyl isocyanide and 2,6-xylyl isocyanide, respectively, the benzenoid analogues of **3** and **5** (i.e., 2,6-dialkoxycarbonylphenyl isocyanide and 2,6-dicyanophenyl isocyanide), are presently unknown. In our experience, free 2-isocyano-1,3-dibromoazulene **4**, which would be analogous to 2,6-dibromophenyl isocyanide [38], proved to have quite limited thermal stability and is, therefore, best formed through 1,3-bromination of the already coordinated “parent” 2-isocyanoazulene **1**. The π-acceptor/σ-donor ratio of the 1,3-X_2_-substituted isocyanoazulene ligands increases in the order of X being CH_3_ ≈ H < CO_2_Et < Br << CN. The relative π-acceptor/σ-donor ratio of 2-isocyano-1,3-dicyanoazulene **5** rivals those of perfluorinated CNC_6_F_5_ and CNC_2_F_3_ ligands. In the context of the *net* π-acidity of isocyanoarenes, the upper limit has belonged to CNC_6_F_5_ to date. Thus, cyanation is an effective alternative to polyfluorination in enhancing the *net* π-acidity of isocyanoarene ligands, as we demonstrated in this study for the isocyanide ligand platform featuring the highly polarizable azulenic π-system with a relatively small aromatic delocalization stabilization energy [39].

## 4. Materials and Methods

### 4.1. General Procedures, Starting Materials, and Equipment

Synthetic operations that required inert atmosphere conditions were performed under 99.5% argon purified by passage through columns of activated BASF catalyst and molecular sieves. All connections involving the gas purification systems were made of glass, metal, or other materials impermeable to air. Solutions were transferred via stainless steel cannulas whenever possible. Standard Schlenk techniques were employed with a double manifold vacuum line. Dichloromethane was distilled over CaH_2_. Tetrahydrofuran (THF) and toluene were distilled over Na/benzophenone. Pentane was distilled over Na/benzophenone dissolved in a minimum amount of diglyme. Triethylamine was distilled over NaOH. Following purification, all distilled solvents were stored under argon. Deuterated chloroform was purchased from Cambridge Isotope Laboratories (Tewksbury, MA, USA) and stored over activated molecular sieves.

Infrared spectra were recorded on a PerkinElmer Spectrum 100 FTIR spectrometer with samples sealed in 0.1 mm NaCl cells. NMR samples were analyzed on Bruker Avance 400 or 500 spectrometers. ^1^H and ^13^C NMR chemical shifts are given with reference to residual solvent resonances relative to Si(CH_3_)_4_. ^14^N NMR chemical shifts are referenced to liquid NH_3_ at 25 °C. A solution of N,N-dimethylformamide in CD_2_Cl_2_ was used as an external ^14^N NMR reference {δ(^14^N) = 103.8 ppm vs. liquid NH_3_ at 25 °C}. UV-vis spectra were recorded in CH_2_Cl_2_ at 24 °C using a CARY 100 spectrophotometer.

Cyclic voltammetric (CV) experiments on ca. 0.02 mM solutions of **6**, **7**, **8**, and **10** were conducted at room temperature using an EPSILON (Bioanalytical Systems, INC., West Lafayette, IN) electrochemical workstation. The electrochemical cell was placed in an argon-filled Vacuum Atmospheres glovebox. Tetrabutylammonium hexafluorophosphate ([^n^Bu_4_N][PF_6_], 0.1 M solution in CH_2_Cl_2_) was used as the supporting electrolyte. CV data were recorded using a three-component system consisting of a platinum working electrode, platinum wire auxiliary electrode, and a glass encased non-aqueous silver/silver chloride reference electrode with a scan rate of 100 mV/s. The reference Ag/Ag^+^ electrode was monitored with the ferrocenium/ferrocene couple. Prior to each CV scan, IR compensation was achieved by measuring the uncompensated solution resistance followed by incremental compensation and circuit stability testing. Background CV scans of the electrolyte solution were recorded before adding the analytes. All potentials (E_1/2_) were referenced to an external ferrocene/ferrocenium couple.

Melting points are uncorrected and were determined for samples in capillary tubes sealed under argon. Elemental analyses (C, H, N) were carried out by Chemisar/Guelph Chemical Laboratories Ltd., ON, Canada or by Micro-Analysis Inc., Wilmington, DE, USA. 2-Amino-1,3-diethoxycarbonylazulene [40], acetic-formic anhydride [41], 2-isocyanoazulene (**1**) [13], 2-isocyano-1,3-diethoxycarbonylazulene (**3**) [20], 2-isocyano-1,3-dicyanoazulene (**5**) [21], and [(OC)_5_Cr(2-isocyano-1,3-diethoxycarbonylazulene)] (**8**) [19] were prepared according to the literature procedures. All other reagents were obtained from commercial sources and used as received. Davisil (200–425 mesh, type 60 Å) silica gel was used for chromatographic purifications.

All Density Functional Theory (DFT) calculations were performed using the ORCA (v.3.0.1) program [42]. Geometric optimizations for azulene and **10** were performed using the BP86 functional [43,44] with a Def2-TZVP (Alrichs triple-ζ valence polarized basis set) [45,46]. The resolution of identity approximation (RI) was used along with the Def2-TZVP/J auxiliary basis set [47]. Single point energy calculations used to create images of orbital densities were then performed using the B3LYP functional [48,49,50], a Def2-TZVP basis set, and a Def2-TZVP/J auxiliary basis set [47,51]. The Cartesian coordinates for the DFT-optimized structures of azulene and **10** are provided in Tables S6 and S7.

### 4.2. Synthesis of 2-Formamido-1,3-Dimethylazulene

A 60 wt. % solution of sodium bis(2-methoxyethoxy)aluminum dihydride (Red-Al^®^) in toluene (2.50 mL, 8.74 mmol) was slowly added to a cold (0 °C) solution of 2-amino-1,3-diethoxycarbonylazulene (0.2513 g, 0.8746 mmol) in 25 mL of toluene over a period of 1 h. The reaction mixture was stirred for 15 h at 70 °C, at which point the reaction flask was opened to air, and its content was poured slowly into a beaker containing 50 mL of 10% aqueous NaOH. After 15 min of stirring to quench any remaining Red-Al^®^, the organic layer was separated, and the aqueous fraction was extracted with Et_2_O (1 × 30 mL). The organic fractions were combined, dried over anhydrous MgSO_4_, and filtered. All solvent was removed from the filtrate in vacuo. The resulting dark red oil (presumably, 2-amino-1,3-dimethylazulene) was dissolved in a minimum amount of CH_2_Cl_2_, and this solution was added to a freshly prepared mixture of formic acid (3.50 mL) and acetic-formic anhydride (2.95 mL). After stirring for 1.5 h, all volatiles were removed on a rotary evaporator, and the residue was treated with 100 mL of 10% aqueous NaHCO_3_ with stirring to quench/neutralize any remaining anhydride/acid. The aqueous phase was extracted with CH_2_Cl_2_ (2 × 20 mL). The organic fractions were combined and dried over anhydrous MgSO_4_. Filtration followed by solvent removal from the filtrate on a rotary evaporator produced a dark residue, which was subjected to column chromatography on silica gel using 15 vol. % Et_2_O in CH_2_Cl_2_ as eluent. A blue band was collected, which gave 2-formamido-1,3-dimethylazulene (0.0945 g, 0.4743 mmol) in a 55% yield as a blue powder after solvent removal and drying of the solid at 10^−2^ torr. Mp: 178–181 °C. In a CDCl_3_ solution, 2-formamido-1,3-dimethylazulene exists as a mixture of two rotamers due to hindered rotation about the C-N bond. In the following NMR data, resonances corresponding to the minor (*cis*) rotamer are designated with an asterisk. ^1^H NMR (400 MHz, CDCl_3_, 25 °C): δ 2.60 (s, 6H, C*H*_3_), 7.12 (t, 2H, *H*^5,7^, ^3^*J*_HH_ = 10 Hz), 7.51 (t, 1H, *H*^6^, ^3^*J*_HH_ = 10 Hz), 8.08 (d, 1H, N*H*, ^3^*J*_HH_ = 11 Hz), 8.15 (d, 2H, *H*^4,8^, ^3^*J*_HH_ = 10 Hz), 8.70 (d, 1H, C*H*O, ^3^*J*_HH_ = 11 Hz); 2.53 (s, 6H, C*H*_3_*), 7.05 (t, 2H, *H*^5,7^*, ^3^*J*_HH_ = 10 Hz), 7.41 (s, 1H, NH*), 7.48 (t, 1H, H^6^*, ^3^*J*_HH_ = 10 Hz), 8.15 (d, 2H, *H*^4,8*^, ^3^*J*_HH_ = 10 Hz), 8.55 (s, 1H, C*H*O*) ppm. ^13^C{^1^H} NMR (125.8 MHz, CDCl_3_, 25 °C): δ 10.5 (*C*H_3_), 115.8, 122.4, 132.5, 135.9, 136.3, 141.2 (azulenic C), 164.2 (*C*HO); 10.6 (*C*H_3_*) ppm.

### 4.3. Synthesis of 2-Isocyano-1,3-Dimethylazulene (2)

Excess phosphorous oxychloride (0.243 mL, 2.61 mmol) was added to a solution of 2-formamido-1,3-dimethylazulene (0.450 g, 2.26 mmol) and freshly distilled triethylamine (7.86 mL) in 50 mL of dry CH_2_Cl_2_ at room temperature. The reaction mixture was stirred for 0.5 h and then quenched with 100 mL of 10% aqueous NaHCO_3_. The organic layer was separated, and the aqueous phase was extracted with CH_2_Cl_2_ (3 × 30 mL). The organic fractions were combined, dried over anhydrous MgSO_4_, and filtered. Solvent removal from the filtrate in vacuo gave a green microcrystalline product, which was subjected to column chromatography on silica gel using neat CH_2_Cl_2_ as eluent. A dark aqua-blue band was collected, which afforded green crystalline **2** (0.379 g, 2.09 mmol) in a 93% yield following solvent removal and drying at 10^-2^ torr. The isocyanide **2** can be further purified via recrystallization from hexanes (slow evaporation). Mp: 99–101 °C. Anal. Calcd. for C_13_H_11_N: C, 86.15; H, 6.12; N, 7.73. Found: C, 85.53; H, 5.90; N, 7.77. IR (CH_2_Cl_2_): υ_N≡C_ 2115 cm^−1^. ^1^H NMR (400 MHz, CDCl_3_, 25 °C): δ 2.64 (s, 6H, C*H*_3_), 7.09 (t, 2H, *H*^5,7^, ^3^*J*_HH_ = 10 Hz), 7.55 (t, 1H, *H*^6^, ^3^*J*_HH_ = 10 Hz), 8.18 (d, 2H, *H*^4,8^, ^3^*J*_HH_ = 10 Hz) ppm. ^13^C{^1^H} NMR (125.8 MHz, CDCl_3_, 25 °C): 10.1 (*C*H_3_), 120.2, 122.7, 134.8, 135.7, 139.2 (azulenic *C*), 170.7 (isocyano *C*) ppm. ^14^N NMR (36.2 MHz, CDCl_3_, 25 °C): 172.3 ppm. UV-vis (CH_2_Cl_2_, λ_max_ (log ε)): 732 (2.34), 671 (2.68), 624 (2.70), 354 (3.81), 338 (3.68), 298 (4.71), 290 (4.80), 242 (4.26) nm.

### 4.4. Synthesis of [(OC)_5_Cr(2-Isocyanoazulene)] (6)

A red-orange solution of [Cr(CO)_5_(THF)] was prepared in situ by photolysis of Cr(CO)_6_ (0.120 g, 0.540 mmol) dissolved in 50 mL of THF using a Hanovia Hg 450 W immersion lamp. Upon completion of the photolysis as judged by IR of the mixture in the υ_C≡O_ region, a solution of 2-isocyanoazulene (0.125 g, 0.816 mmol) in 50 mL of THF was added via cannula to the [Cr(CO)_5_(THF)] solution at room temperature. The resulting mixture was stirred for 18 h. The reactor was then opened to air, and its content was concentrated to dryness under vacuum. The residue was subjected to column chromatography on silica gel using 1:1 CH_2_Cl_2_ / hexanes eluent. The first eluted band, dark green in color, afforded microcrystalline **6** (0.112 g, 0.324 mmol) in a 60% yield after solvent removal and drying of the product at 10^−2^ torr. Mp: 137–139 °C. Anal. Calcd. for C_16_H_7_CrNO_5_: C, 55.67; H, 2.04; N, 4.06. Found: C, 55.34; H, 2.08; N, 3.97. IR (CH_2_Cl_2_): υ_N≡C_ 2138 m, υ_C≡O_ 2052 m (A_1_^(1)^), 1999 vw (B_1_), 1957 s (A_1_^(2)^ + E) cm^−1^. ^1^H NMR (400 MHz, CDCl_3_, 25 °C): δ 7.29 (s, 2H, *H^1,3^*), 7.33 (t, 2H, *H^5,7^*, ^3^*J*_HH_ = 10 Hz), 7.69 (t, 1H, *H^6^*, ^3^*J*_HH_ = 10 Hz), 8.34 (d, 2H, *H^4,8^*, ^3^*J*_HH_ = 10 Hz) ppm. ^13^C{^1^H} NMR (CDCl_3_, 125.8 MHz, 25 °C): δ 113.68, 125.59, 131.44, 138.61, 139.16 (azulenic *C*), 175.71 (isocyano *C*), 214.64 (*C*O, *cis*), 216.90 (*C*O, *trans*) ppm. UV-vis (CH_2_Cl_2_, λ_max_ (log ε)): 409 (4.21), 284 (4.83), 236 (4.69) nm.

### 4.5. Synthesis of [(OC)_5_Cr(2-Isocyano-1,3-Dimethylazulene)] (7)

A red-orange solution of [Cr(CO)_5_(THF)] was prepared in situ by photolysis of Cr(CO)_6_ (0.097 g, 0.441 mmol) dissolved in 60 mL of THF over a period of 2.5 h using a Hanovia Hg 450 W immersion lamp. A solution of **2** (0.100 g, 0.552 mmol) in 20 mL of THF was added via cannula to the Cr(CO)_5_(THF) solution at room temperature. The reaction mixture gradually turned dark green and was stirred for 16 h. All solvent was removed under vacuum to provide a dark residue, which was subjected to column chromatography on silica gel using neat CH_2_Cl_2_ to elute a dark green band. The collected dark green solution was then concentrated to dryness under reduced pressure. The resulting solid was recrystallized via slow evaporation of CH_2_Cl_2_ to afford green crystals of **7** (0.110 g, 0.294 mmol) in a 67% yield. Mp: 160–164 °C (dec). Anal. Calcd. for C_18_H_11_CrNO_5_: C, 57.92; H, 2.97; N, 3.75. Found: C, 57.98; H, 3.11; N, 3.72. IR (CH_2_Cl_2_): υ_N__≡C_ 2129 m, υ_C__≡O_ 2001 w (B_1_), 2050 m (A_1_^(1)^), 1957 s (A_1_^(2)^ + E) cm^-1^. ^1^H NMR (500 MHz, CDCl_3_, 25 °C): δ 2.63 (s, 6H, C*H*_3_), 7.10 (t, 2H, *H^5,7^*, ^3^*J*_HH_ = 10 Hz), 7.53 (t, 1H, *H^6^*, ^3^*J*_HH_ = 10 Hz), 8.17 (d, 2H, *H^4,8^*, ^3^*J*_HH_ = 10 Hz) ppm. ^13^C{^1^H} NMR (CDCl_3_, 125.8 MHz, 25 °C): δ 10.02 (*C*H_3_), 119.70, 122.84, 134.87, 135.30, 138.88 (azulenic *C*), 177.99 (isocyano *C*), 214.68 (*C*O, *cis*), 216.91 (*C*O, *trans*) ppm. UV-vis (CH_2_Cl_2_, λ_max_ (log ε)): 724 (2.76), 620 (2.91), 410 (4.23) nm.

### 4.6. Synthesis of [(OC)_5_Cr(2-Isocyano-1,3-Dibromoazulene)] (9)

*N*-Bromosuccinimide (0.017 g, 0.096 mmol) was added to a solution of **6** (0.017 g, 0.048 mmol) in 50 mL of CH_2_Cl_2_. The resulting mixture was stirred for 1.5 h at room temperature. The solvent was then removed under reduced pressure to give a green residue, which was subjected to column chromatography on silica gel using neat hexanes to elute a light green band. This green solution was concentrated to dryness, and the solid was dried at 10^−2^ torr to afford green powdered **9** (0.023 g, 0.046 mmol) in a 96% yield. Anal. Calcd. for C_16_H_5_CrNO_5_Br_2_: C, 38.20; H, 1.00; N, 2.78. Found: C, 37.58; H, 1.21; N, 2.68. IR (CH_2_Cl_2_): υ_N≡C_ 2132 m, υ_C≡O_ 2042 s (A_1_^(1)^), 1963 vs (A_1_^(2)^ + E) cm^−1^. ^1^H NMR (400 MHz, CDCl_3_, 25 °C): δ 7.42 (t, 2H, *H^5,7^*, ^3^*J*_HH_ = 10 Hz), 7.74 (t, 1H, *H^6^*, ^3^*J*_HH_ = 10 Hz), 8.33 (d, 2H, *H^4,8^*, ^3^*J*_HH_ = 10 Hz) ppm. ^13^C{^1^H} NMR (CDCl_3_, 125.8 MHz, 25 °C): δ 98.77, 126.32, 135.12, 138.16, 140.93, (azulenic *C*), 186.07 (isocyano *C*), 214.10 (*C*O, *cis*), 216.08 (*C*O, *trans*) ppm. UV-vis (CH_2_Cl_2_, λ_max_ (log ε)): 440 (4.19), 356 (4.19), 299 (4.70), 235 (4.71) nm.

Note: Free 2-isocyano-1,3-dibromoazulene ligand **4** can be generated via bromination of 2-aminoazulene with 2 equivalents of N-bromosuccinimide followed by formylation of the resulting 2-amino-1,3-dibromoazulene and subsequent dehydration of the resulting 2-formamido-1,3-dibromoazulene using the procedure described for the synthesis of **2**. However, while **4** was characterized in solution by FTIR and NMR, this compound proved to be exceedingly thermally sensitive to be isolated pure in the solid state. IR (CH_2_Cl_2_): υ_N__≡C_ 2121 cm^−1^. ^1^H NMR (400 MHz, CDCl_3_, 25 °C): δ 7.42 (t, 2H, *H*^5,7^, ^3^*J*_HH_ = 10 Hz), 7.78 (t, 1H, *H*^6^, ^3^*J*_HH_ = 10 Hz), 8.38 (d, 2H, *H*^4,8^, ^3^*J*_HH_ = 10 Hz) ppm. ^13^C{^1^H} NMR (100.6 MHz, CDCl_3_, 25 °C): 99.11, 126.23, 134.91, 139.10, 141.74 (azulenic *C*), 175.30 (isocyano *C*) ppm.

### 4.7. Synthesis of [(OC)_5_Cr(2-Isocyano-1,3-Dicyanoazulene)] (10)

A red-orange solution of [Cr(CO)_5_(THF)] was prepared in situ by photolysis of Cr(CO)_6_ (0.145 g, 0.659 mmol) dissolved in 70 mL of THF over a period of 3 h using a Hanovia Hg 450 W immersion lamp. A solution of 2-isocyano-1,3-dicyanoazulene (0.200 g, 0.984 mmol) in 20 mL of THF was added via cannula to the [Cr(CO)_5_(THF)] solution at room temperature. The reaction mixture gradually turned dark brown and was stirred for 17 h. All solvent was removed under vacuum to provide a dark orange-brown residue, which was subjected to column chromatography on silica gel using neat CH_2_Cl_2_. The orange-colored third band was collected and concentrated to dryness under reduced pressure. The resulting orange-brown residue was recrystallized via slow evaporation of CH_2_Cl_2_ to afford orange crystalline **10** (0.050 g, 0.126 mmol) in a 19% yield. Mp: 197–201 °C (dec). Anal. Calcd. for C_18_H_5_CrN_3_O_5_: C, 54.70; H, 1.28; N, 10.63. Found: C, 54.60; H, 1.47; N, 10.43. IR (CH_2_Cl_2_): υ_C≡N_ 2222 w, υ_N≡C_ 2120 m, υ_C≡O_ 2025 s (A_1_^(1)^), 1972 s (A_1_^(2)^ + E) cm^−1^. ^1^H NMR (500 MHz, CDCl_3_, 25 °C): δ 7.95 (t, 2H, *H^5,7^*, ^3^*J*_HH_ = 10 Hz), 8.16 (t, 1H, *H^6^*, ^3^*J*_HH_ = 10 Hz), 8.74 (d, 2H, *H^4,8^*, ^3^*J*_HH_ = 10 Hz) ppm. ^13^C{^1^H} NMR (CDCl_3_, 125.8 MHz, 25 °C): δ 95.16 (cyano *C*), 112.57, 133.00, 139.72, 142.63, 143.14 (azulenic *C*), 194.69 (isocyano *C*), 213.09 (*C*O, *cis*), 214.65 (*C*O, *trans*) ppm. UV-vis (CH_2_Cl_2_, λ_max_ (log ε)): 734 (2.55), 474 (4.32), 360 (4.23) nm.

### 4.8. X-ray Crystallographic Work

Single-crystal X-ray diffraction data were collected using graphite-monochromated MoKα radiation (λ = 0.71073 Å) on Bruker APEX 2 diffractometers equipped with a SMART CCD area detector. The Cambridge Crystallographic Data Centre (CCDC) entries 1,536,382, 1,536,384, 1,536,383, 1,449,131, 1,536,385 contain the supplementary crystallographic data for compounds **2**, **6**, **7**, **8**, and **10**, respectively. These data can be obtained free of charge via www.ccdc.cam.ac.uk/data_request/cif; by emailing data_request@ccdc.cam.ac.uk; or by contacting The Cambridge Crystallographic Data Centre, 12, Union Road, Cambridge CB2 1EZ, UK; fax: +44-1223-336033.

Crystal data for **2**, C_13_H_11_N (M = 181.23 g/mol): orthorhombic, space group Pbca (no. 61), a = 13.789(3) Å, b = 9.154(2) Å, c = 15.737(3) Å, V = 1986.3(6) Å^3^, Z = 8, T = 100(2) K, μ(MoKα) = 0.071 mm^−1^, D_calc_ = 1.212 g/cm^3^, 20632 reflections collected (3.70° ≤ Θ ≤ 30.10°), 2903 unique (R_int_ = 0.070). The final R1 was 0.058 (I > 2σ(I)), and wR2 was 0.155 (all data).

Crystal data for **6**, C_16_H_7_CrNO_5_ (M = 345.23 g/mol): monoclinic, space group P21/c (no. 14), a = 7.3181(8) Å, b = 33.828(4) Å, c = 13.3970(12) Å, β = 115.582(5), V = 2991.4(6) Å^3^, Z = 8, T = 100(2) K, μ(MoKα) = 0.789 mm^−1^, D_calc_ = 1.533 g/cm^3^, 29797 reflections collected (1.20° ≤ Θ ≤ 26.41°), 6118 unique (R_int_ = 0.0956). The final R1 was 0.0522 (I > 2σ(I)), and wR2 was 0.1303 (all data).

Crystal data for **7**, C_18_H_11_CrNO_5_ (M = 373.28 g/mol): monoclinic, space group P21/c (no. 14), a = 14.0814(9) Å, b = 16.5225(10) Å, c = 7.3496(5) Å, β = 103.8080(1), V = 1660.54(18) Å^3^, Z = 4, T = 100(2) K, μ(MoKα) = 0.717 mm^−1^, D_calc_ = 1.493 g/cm^3^, 23401 reflections collected (1.49° ≤ Θ ≤ 29.52°), 4610 unique (R_int_ = 0.0210). The final R1 was 0.0340 (I > 2σ(I)), and wR2 was 0.0933 (all data).

Crystal data for **8**, C_22_H_15_CrNO_9_ (M = 489.35 g/mol): monoclinic, space group C2/c (no. 15), a = 23.9424(14) Å, b = 15.9966(9) Å, c = 12.3639(7) Å), β = 116.633(1), V = 4232.9(4) Å^3^, Z = 8, T = 100(2) K, μ(MoKα) = 0.596 mm^−1^, D_calc_ = 1.536 g/cm^3^, 27455 reflections collected (1.59° ≤ Θ ≤ 27.88°), 5047 unique (R_int_ = 0.0337). The final R1 was 0.0343 (I > 2σ(I)), and wR2 was 0.0864 (all data).

Crystal data for **10**, C_18_H_5_CrN_3_O_5_ (M = 395.25 g/mol): triclinic, space group P-1 (no. 2), a = 6.1716(14) Å, b = 9.244(2) Å, c = 15.070(3) Å, α = 85.048(4)°, β = 87.266(4)°, γ = 76.705(4)°, V = 833.2(3) Å^3^, Z = 2, T = 100(2) K, μ(MoKα) = 0.723 mm^−1^, D_calc_ = 1.575 g/cm^3^, 9244 reflections collected (1.36° ≤ Θ ≤ 25.00°), 2929 unique (R_int_ = 0.0309). The final R1 was 0.0426 (I > 2σ(I)), and wR2 was 0.1089 (all data).

## Figures and Tables

**Table 1:** Selected FTIR, ^13^C NMR, and ^14^N NMR data for **1**, **2**, and 2,6-CNXyl.

CNR	υ_N≡C_, cm^−1^	δ(*^13^C*≡N), ppm	δ(*C*≡*^14^N*), ppm
**1**	2127 ^1^	168.6 ^2,5^	175.3 ^3,5^
**2**	2115 ^1^	170.7 ^2^	172.3 ^3^
2,6-CNXyl	2117 ^1^	167.7 ^2^	175.6 ^4,6^

^1^ In CH_2_Cl_2_. ^2^ In CDCl_3_ vs. Si(*C*H_3_)_4_. ^3^ In CDCl_3_ vs. NH_3_(l). ^4^ In CH_2_Cl_2_ vs. NH_3_(l). ^5^ [13]. ^6^ [29].

**Table 2:** Selected bond distances (Å) and angles (°) for [(OC)_5_Cr(CNR)] complexes.

Complex	d(Cr–CN)	d(C≡N)	d(Cr-CO_trans_)	∠C-N-C
[(OC)_5_Cr(CN^t^Bu)] ^1^	2.016	1.150	1.872	177.9
**6** ^2^	1.974(4) 1.987(4)	1.155(4) 1.153(4)	1.879(4) 1.882(4)	178.(4) 170.5(3)
**7**	1.981(1)	1.165(2)	1.885(1)	178.9(2)
**8**	1.977(2)	1.163(2)	1.881(2)	168.9(2)
[(OC)_5_Cr(CN*p*-FAr^DArF2^)] ^3^	1.967(2)	1.162(3)	1.894(2)	172.6(2)
[(OC)_5_Cr(CNC_2_F_3_)] ^4^	1.942(2)	1.162(2)	1.909(2)	173.6(2)
**10**	1.937(2)	1.176(3)	1.901(3)	164.8(3)

^1^ [32]. ^2^ Data for two crystallographically independent molecules in the unit cell of **6**. ^3^ [6]. ^4^ [10].

**Table 3:** Properties of the MLCT (L = CNAz) bands observed in the electronic absorption spectra of **6**, **7**, **8**, **9**, and **10** in CH_2_Cl_2_ at 24 °C.

(OC)_5_Cr(CNAz) Complex	λ_max_, nm	υ_max_, cm^−1^
**6**	409	24,450
**7**	410	24,390
**8**	441 ^1^	22,676
**9**	440	22,727
**10**	474	21,097

^1^ [19].

**Table 4:** Infrared signatures of **6**–**10** and [(OC)_5_Cr(CNC_6_F_5_)] in υ_N≡C_ and υ_C≡O_ regions ^1^.

Complex	υ_N≡C_(A_1_), cm^−1^	υ_C≡O_(A_1_^(1)^), cm^−1^	ν_C__≡O_(A_1_^(2)^), cm^−1^	ν_C__≡O_(E), cm^−1^	k_C≡O,trans_, mdyne/Å
**7** ^2^	2129	2050	1957	1957	15.767
**6** ^2^	2138	2052	1957	1957	15.774
**8** ^2^	2140	2049	1959	1959	15.789
**9** ^2^	2132	2042	1963	1963	15.816
**10** ^2^	2120	2025	1972	1972	15.875
[(OC)_5_Cr(CNC_6_F_5_)] ^3^	2125	2041	1968	1968	15.877

^1^ Complexes are listed in order of increasing k_C≡O,trans_ magnitude. ^2^ In CH_2_Cl_2_. ^3^ In pentane [12].

**Table 5:** ^13^C NMR data for the [(OC)_5_Cr(CN)] core in complexes [(OC)_5_Cr(CNR)] dissolved in CDCl_3_.

Complex	δ(*^13^C*N), ppm	δ(*^13^C*O_trans_), ppm	δ(*^13^C*O_cis_), ppm
**6**	175.64	216.83	214.58
**7**	177.99	216.91	214.68
**8** ^1^	183.36	216.69	214.60
**9**	186.07	216.08	214.10
**10**	194.69	214.65	213.09
[(OC)_5_Cr(CNC_6_F_5_)] ^2^	193.8	214.6	213.3
[(OC)_5_Cr(CNC_2_F_3_)] ^3^	199.3	214.2	213.0
[(OC)_5_Cr(CNC(ClF)C(ClF_2_))] ^3^	208.2	212.0	212.0
[(OC)_5_Cr(CNCF_3_)] ^4^	211.1	211.5	211.7

^1^ [19]. ^2^ [12]. ^3^ [10]. ^4^ [8].

## Supplementary Materials

The following are available online: X-ray crystallographic details for **2**, **6**, **7**, **8**, and **10**; ^13^C NMR δ(CO_cis_) vs. δ(CN) trend plot, cyclic voltammograms for **6** and **7**, and XYZ Cartesian coordinates for azulene and **10**.

## References

[1] Hoffmann R. (1982). Building Bridges Between Inorganic and Organic Chemistry (Nobel Lecture). Angew. Chem. Int. Ed..

[2] Giustiniano M., Basso A., Mercalli V., Massarotti A., Novellino E., Tron G.C., Zhu J. (2017). To each his own: Isonitriles for all flavors. Functionalized isocyanides as valuable tools in organic synthesis. Chem. Soc. Rev..

[3] Barybin M.V., Meyers J.J.J., Neal B.M. (2013). ChemInform Abstract: Renaissance of Isocyanoarenes as Ligands in Low-Valent Organometallics. Chemin.

[4] Treichel P.M. (1973). Transition Metal-Isocyanide Complexes. Adv. Organomet. Chem..

[5] Boyarskiy V.P., Bokach N.A., Luzyanin K.V., Kukushkin V.Y. (2015). Metal-Mediated and Metal-Catalyzed Reactions of Iso-cyanides. Chem. Soc. Rev..

[6] Carpenter A.E., Mokhtarzadeh C.C., Ripatti D.S., Havrylyuk I., Kamezawa R., Moore C.E., Rheingold A.L., Figueroa J.S. (2015). Comparative Measure of the Electronic Influence of Highly Substituted Aryl Isocyanides. Inorg. Chem..

[7] Barnett B.R., Figueroa J.S. (2016). Zero-valent isocyanides of nickel, palladium and platinum as transition metal σ-type Lewis bases. Chem. Commun..

[8] Lentz D. (1984). Trifluormethylisocyanid, ein starker π-Akzeptor-Ligand – Pentacarbonyl(thrifluormethylisocyanid)chrom und –wolfram. Chem. Ber..

[9] Oberhammer H., Lentz D. (1985). Gas-phase structure of pentacarbonyl(trifluoromethyl isocyanide)chromium, (CF3NC)Cr(CO)5. Inorg. Chem..

[10] Lentz D., Preugschat D. (1992). Preparation of the First Fluorinated Alkenyl lsocyanide F2C=CF-NC by Flash Vacuum Pyrolysis of [Cr(CO)5(CN-CF=CF2)]. Crystal and Molecular Structure of [Cr(CO)5(CN-CF=CF2)] at 125 K. Chem. Commun..

[11] Mujkic M., Lentz D. (2012). Synthesis of highly unsaturated isocyanides via organometallic pathways. Dalton Trans..

[12] Lentz D., Anibarro M., Preugschat D., Bertrand G. (1998). Transition metal complexes and cycloaddition products of pentafluor-ophenyl isocyanide. J. Fluorine Chem..

[13] Robinson R.E., Holovics T.C., Deplazes S.F., Powell D.R., Lushington G.H., Thompson W.H., Barybin M.V. (2005). Five Possible Isocyanoazulenes and Electron-Rich Complexes Thereof: A Quantitative Organometallic Approach for Probing Electronic Inhomogeneity of the Azulenic Framework. Organometallics.

[14] Anderson A.G., Steckler B.M. (1959). Azulene. VIII. A Study of the Visible Absorption Spectra and Dipole Moments of Some 1- and 1,3-Substituted Azulenes1,2. J. Am. Chem. Soc..

[15] Liu R.S.H., Asato A.E. (2003). Tuning the color and excited state properties of the azulenic chromophore: NIR absorbing pigments and materials. J. Photochem. Photobiol. C Photochem. Rev..

[16] Barybin M.V. (2010). Nonbenzenoid aromatic isocyanides: New coordination building blocks for organometallic and surface chemistry. Co-ord. Chem. Rev..

[17] Sun S., Zhuang X., Wand L., Zhang B., Ding J., Zhang F., Chen Y. (2017). Azulene-Bridged Coordinated Framework Based Qua-si-Molecular Rectifier. J. Mater. Chem. C..

[18] Yang C., Schellhammer K.S., Ortmann F., Sun S., Dong R., Karakus M., Mics Z., Löffler M., Zhang F., Zhuang X. (2017). Coordination Polymer Framework Based On-Chip Micro-Supercapacitors with AC Line-Filtering Performance. Angew. Chem. Int. Ed..

[19] Applegate J.C., Okeowo M.K., Erickson N.R., Neal B.M., Berrie C.L., Gerasimchuk N.N., Barybin M.V. (2016). First π-linker featuring mercapto and isocyano anchoring groups within the same molecule: Synthesis, heterobimetallic complexation and self-assembly on Au(III). Chem. Sci..

[20] DuBose D.L., Moody D., Robinson R.E., Holovics T.C., Weintrob E.C., Berrie C.L., Barybin M.V. (2006). Interaction of Mono- and Diisocyanoazulene Derivatives with Gold Surface: First Examples of Self-Assembled Monolayer Films Involving Azulenic Scaffolds. Langmuir.

[21] Neal B.M., Vorushilov A.S., Delarosa A.M., Robinson R.E., Berrie C.L., Barybin M.V. (2011). Ancillary nitrile substituents as convenient IR spectroscopic reporters for self-assembly of mercapto- and isocyanoazulenes on Au(III). Chem. Commun..

[22] Maher T.R., Spaeth A.D., Neal B.M., Berrie C.L., Thompson W.H., Day V.W., Barybin M.V. (2010). Linear 6,6’-Biazulenyl Framework Featuring Isocyanide Termini: Synthesis, Structure, Redox Behavior, Complexation, and Self-assembly on Gold(III). J. Am. Chem. Soc..

[23] Kühne T., Au-Yeung K.H., Eisenhut F., Aiboudi O., Ryndyk D.A., Cuniberti G., Lissel F., Moresco F. (2020). STM induced ma-nipulation of azulene-based molecules and nanostructures: The role of the dipole moment. Nanoscale.

[24] Fathi-Rasekh M., Rohde G.T., Hart M.D., Nakakita T., Zatsikha Y.V., Valiev R.R., Barybin M.V., Nemykin V.N. (2019). Posi-tional Isomers of Isocyanoazulenes as Axial Ligands Coordinated to Ruthenium(II) Tetraphenylporphyrin: Fine-Tuning Redox and Optical Profiles. Inorg. Chem..

[25] De Domingo E., Barcenilla M., Martín-Alvarez J.M., Miguel J.A., Coco S. (2020). The 2-isocyanoazulene-gold(I) fragment as a versatile element for organometallic dyes and liquid crystals. Dye. Pigment..

[26] Pirrung M.C., Ghorai S. (2006). Very few isocyanides with non-offensive smell are known: Versatile, Fragrant, Convertible Isonitriles. J. Am. Chem. Soc..

[27] Drakenberg T., Dahlqvist K.-I., Forsén S., Ragnarsson U., Rasmussen S.E., Sunde E., Sørensen N.A. (1970). The Barrier to Internal Rotation in Amides. II. N,N-Dimethytrichloroacetamide. Experimental Errors in the Evaluation of Rate Constants with the NMR Lineshape Method. Acta Chem. Scand..

[28] Patil P., Ahmadian-Moghaddam M., Dömling A. (2020). Isocyanide 2.0. Green Chem..

[29] Minelli M., Maley W.J. (1989). Multinuclear NMR studies of molybdenum and tungsten carbonyl isocyanide complexes. Inorg. Chem..

[30] Mathieson T., Schier A., Schmidbaur H. (2001). Supramolecular chemistry of gold(I) thiocyanate complexes with thiophene, phosphine and isocyanide ligands, and the structure of 2,6-dimethylphenyl isocyanide. J. Chem. Soc. Dalton Trans..

[31] Ree N., Andersen C.L., Kilde M.D., Hammerich O., Nielsen M.B., Mikkelsen K.V. (2018). The quest for determining one-electron redox potentials of azulene-1-carbonitriles by calculation. Phys. Chem. Chem. Phys..

[32] Sattler W., Parkin G. (2009). Synthesis of transition metal isocyanide compounds from carbonyl complexes via reaction with Li[Me3SiNR]. Chem. Commun..

[33] Holovics T.C., Deplazes S.F., Toriyama M., Powell D.R., Lushington G.H., Barybin M.V. (2004). Organometallic Isocyanocyclopentadienides: A Combined Synthetic, Spectroscopic, Structural, Electrochemical and Theoretical Investigation. Organo Met..

[34] Holovics T.C., Robinson R.E., Weintrob E.C., Toriyama M., Lushington A.G.H., Barybin M.V. (2006). The 2,6-Diisocyanoazulene Motif: Synthesis and Efficient Mono- and Heterobimetallic Complexation with Controlled Orientation of the Azulenic Dipole. J. Am. Chem. Soc..

[35] Cotton F.A., Kraihanzel C.S. (1962). Vibrational Spectra and Bonding in Metal Carbonyls. I. Infrared Spectra of Phos-phine-substituted Group VI Carbonyls in the CO Stretching Region. J. Am. Chem. Soc..

[36] Karakaş D., Kaya C. (2001). Force constant calculations for octahedral complexes of the type M(CO)5L, based on the CO-factored force field. J. Organomet. Chem..

[37] Johnston R.F. (1994). Effects of Multiple Substituents on the Physical Properties of Pentacarbonylarylisocyanidechromium Com-pounds. J. Coord. Chem..

[38] Ugi I., Fetzer U., Eholzer U., Knupfer H., Offermann K. (1965). Isonitril-Synthesen. Angew. Chem..

[39] Dewar M.J.S. (1969). The Molecular Orbital Theory of Organic Chemistry.

[40] Nozoe T., Takase K., Nakazawa T., Fukuda S. (1971). The formation of azulene derivatives from 2H-cyclohepta[b]furan-2-one derivatives. Tetrahedron.

[41] Krimen K.I. (1970). Acetic Formic Anhydride. Org. Synth..

[42] Neese F. (2012). ORCA—An ab initio, Density Functional and Semiempirical Program Package, version 3.0.

[43] Becke A.D. (1986). Density functional calculations of molecular bond energies. J. Chem. Phys..

[44] Perdew J.P. (1986). Density-functional approximation for the correlation energy of the inhomogeneous electron gas. Phys. Rev. B.

[45] Schäfer A., Horn H., Ahlrichs R. (1992). Fully optimized contracted Gaussian basis sets for atoms Li to Kr. J. Chem. Phys..

[46] Schäfer A., Huber C., Ahlrichs R. (1994). Fully optimized contracted Gaussian basis sets of triple zeta valence quality for atoms Li to Kr. J. Chem. Phys..

[47] Neese F. (2003). An improvement of the resolution of the identity approximation for the formation of the Coulomb matrix. J. Comput. Chem..

[48] Becke A.D. (1997). Density-functional thermochemistry. V. Systematic optimization of exchange-correlation functionals. J. Chem. Phys..

[49] Becke A.D. (1993). A new mixing of Hartree-Fock and local density-functional theories. J. Chem. Phys..

[50] Lee C., Yang W., Parr R.G. (1988). Development of the Colle-Salvetti correlation-energy formula into a functional of the electron density. Phys. Rev. B.

[51] Izsák R., Neese F. (2011). An overlap fitted chain of spheres exchange method. J. Chem. Phys..

