# Supplementary material for: Tuning π-Acceptor/σ-Donor Ratio of the 2-Isocyanoazulene Ligand: Non-Fluorinated Rival of Pentafluorophenyl Isocyanide and Trifluorovinyl Isocyanide Discovered
Source: Molecules. 2021 Feb 12;26(4):981. doi: 10.3390/molecules26040981 (PMC7918097; doi:10.3390/molecules26040981)
Supplement: Supplementary file 1 [file molecules-26-00981-s001.pdf]

Supplementary Materials

for

Tuning π-Acceptor/σ-Donor Ratio of the 2-Isocyanoazulene Ligand: A Non-Fluorinated Rival of Pentafluorophenyl Isocyanide and Trifluorovinyl Isocyanide Discovered

Mason D. Hart [1], John J. Meyers, Jr. [1,2,*], Zachary A. Wood [1],
Toshinori Nakakita [1], Jason C. Applegate [1], Nathan R. Erickson [1],
Nikolay N. Gerasimchuk [3,*] and Mikhail V. Barybin [1,*]

[1] Department of Chemistry, The University of Kansas, 1567 Irving Hill Road, Lawrence, KS 66045, USA;
[2] Department of Chemistry & Physics, Clayton State University, 2000 Clayton State Blvd., Morrow, GA 30260, USA;
[3] Department of Chemistry, Missouri State University, 901 S. National Ave., Springfield, MO 65897, USA

* To whom correspondence should be addressed, e-mail: mbarybin@ku.edu; NNGerasimchuk@MissouriState.edu; JohnMeyers@clayton.edu

Table of Contents

A. Crystallographic Work 5

A1. X-ray Crystallographic Characterization of **2**. 5

Figure S1. Packing diagram of **2**. 5

Table S1.1. Crystal data and structure refinement for **2** 6

Table S1.2. Atomic coordinates ($\times 10^4$) and equivalent isotropic displacement parameters ($Å^2 \times 10^3$) for **2**. U(eq) is defined as one third of the trace of the orthogonalized U_{ij} tensor 7

Table S1.3. Bond lengths [Å] for **2** 8

Table S1.4. Bond angles [°] for **2** 9

Table S1.5. Anisotropic displacement parameters ($Å^2 \times 10^3$) for **2**. The anisotropic displacement factor exponent takes the form: $-2\pi^2[h^2a^{*2}U_{11} + ... + 2 h k a^* b^* U_{12}]$ 10

Table S1.6. Torsion angles [°] for **2**. 10

A2. X-ray Crystallographic Characterization of **6**. 11

Figure S2. Packing diagram of **6** (view along the a axis). 11

Figure S3. The π-stacked head-to-tail dimers that form 1D columns in the structure of **6**. 11

Figure S4. Two orthogonal views of the π-stacked head-to-tail pair in the structure of **6** 12

Figure S5. One of two crystallographically independent molecules in the solid-state structure of **6** (the other molecule is shown in Figure 5a of the main article). All thermal ellipsoids are drawn at the 50% probability level 13

Table S2.1. Crystal data and structure refinement for **6** 14

Table S2.2. Atomic coordinates and equivalent isotropic displacement parameters ($Å^2$) for **6**. U(eq) is defined as one third of the trace of the orthogonalized U_{ij} tensor. 16

Table S2.3. Bond lengths [Å] for **6** 18

Table S2.4. Bond angles [°] for **6** 19

Table S2.5. Anisotropic displacement parameters ($Å^2$) for **6**. The anisotropic displacement factor exponent takes the form: $-2\pi^2[h^2a^{*2}U_{11} + ... + 2 h k a^* b^* U_{12}]$. 21

Table S2.6. Torsion angles [°] for **6** 23

A3. X-ray Crystallographic Characterization of **7** 24

Table S3.1. Crystal data and structure refinement for **7** 25

Table S3.2. Atomic coordinates and equivalent isotropic displacement parameters ($Å^2$) for **7**. U(eq) is defined as one third of the trace of the orthogonalized U_{ij} tensor 26

Table S3.3. Bond lengths [Å] for **7** 27
Table S3.4. Bond angles [°] for **7** 28
Table S3.5. Anisotropic displacement parameters ($Å^2$) for **7**. The anisotropic displacement factor exponent takes the form: $-2\pi^2[h^2a^{*2}U_{11} + ... + 2 h k a^* b^* U_{12}]$. 29
Table S3.6. Torsion angles [°] for **7** 30
A4. X-ray Crystallographic Characterization of **8** 31
Figure S6. Solid-state structure of **8** showing a minor disorder of one of the ethoxy groups over two positions. All thermal ellipsoids are drawn at the 50% probability level. 31
Table S4.1. Crystal data and structure refinement for **8** 32
Table S4.2. Atomic coordinates and equivalent isotropic displacement parameters ($Å^2$) for **8**. U(eq) is defined as one third of the trace of the orthogonalized U_{ij} tensor. 34
Table S4.3. Bond lengths [Å] for **8** 36
Table S4.4. Bond angles [°] for **8** 37
Table S4.5. Anisotropic displacement parameters ($Å^2$) for **8**. The anisotropic displacement factor exponent takes the form: $-2\pi^2[h^2a^{*2}U_{11} + ... + 2 h k a^* b^* U_{12}]$. 39
Table S4.6. Torsion angles [°] for **8** 41
A5. X-ray Crystallographic Characterization of **10** 42
Table S5.1. Crystal data and structure refinement for **10** 42
Table S5.2. Atomic coordinates and equivalent isotropic displacement parameters ($Å^2$) for **10**. U(eq) is defined as one third of the trace of the orthogonalized U_{ij} tensor. 44
Table S5.3. Bond lengths [Å] for **10** 45
Table S5.4. Bond angles [°] for **10** 46
Table S5.5. Anisotropic displacement parameters ($Å^2$) for **10**. The anisotropic displacement factor exponent takes the form: $-2\pi^2[h^2a^{*2}U_{11} + ... + 2 h k a^* b^* U_{12}]$. 47
Table S5.6. Torsion angles [°] for **10** 48

B. ^{13}C NMR Studies 49
B1. $\delta(^{13}CO_{cis})$ vs. $\delta(^{13}CN)$ NMR Inverse-Linear Trend 49
Figure S7. Plot of ^{13}C NMR chemical shifts $\delta(^{13}CO_{cis})$ vs. $\delta(^{13}CN)$ for the series of $(OC)_5Cr(CNR)$ complexes listed in Table 5. All ^{13}C NMR data were collected for solutions in $CDCl_3$ 49

C. Electrochemical Work 50

C1. Cyclic Voltammograms....................50

Figure S8. Cyclic voltammogram of *ca*. 0.02 M solution of **6** in 0.1 M [$^nBu_4N$][PF_6]/CH_2Cl_2 vs. external Cp_2Fe/Cp_2Fe^+ at 25 °C. Scan rate = 100 mV/s. 50

Figure S9. Cyclic voltammogram of *ca*. 0.02 M solution of **7** in 0.1 M [$^nBu_4N$][PF_6]/CH_2Cl_2 vs. external Cp_2Fe/Cp_2Fe^+ at 25 °C. Scan rate = 100 mV/s. 51

D. Computational Studies 52

D1. Cartesian Coordinates Pertaining to DFT Calculations 52

Table S6. Cartesian coordinates (Å) for the optimized geometry of azulene....................52

Table S7. Cartesian coordinates (Å) for the optimized geometry of **10**. 53

A. Crystallographic Work

A1. X-ray Crystallographic Characterization of 2.

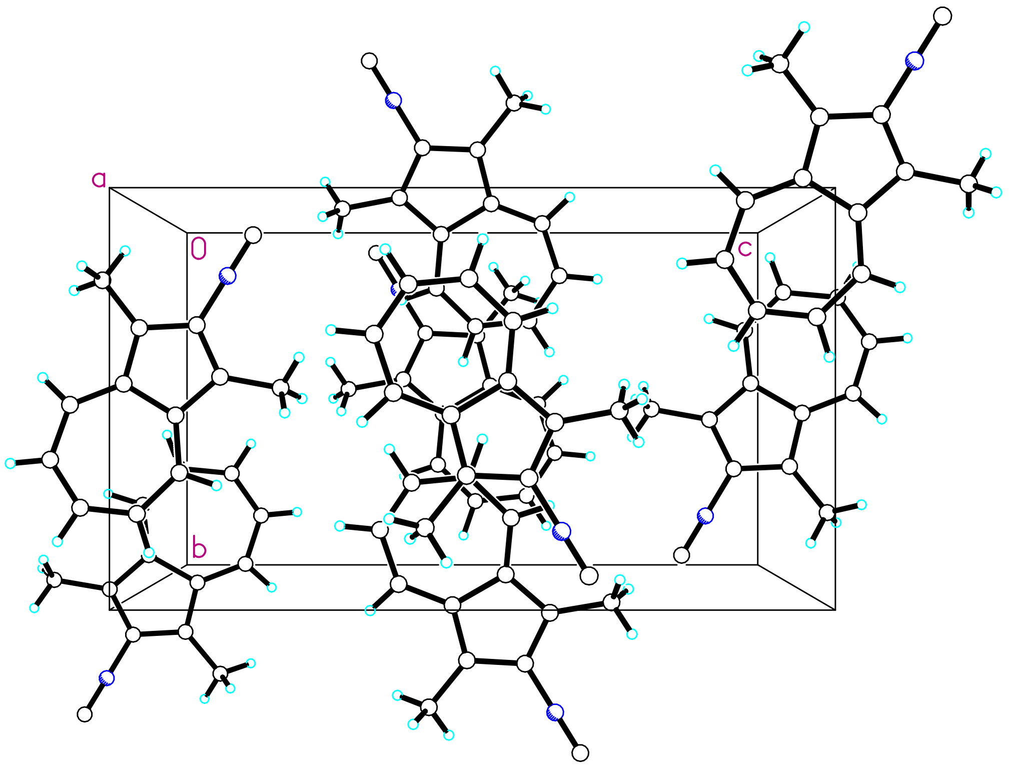

Figure S1. Packing diagram of 2.

Comments

The asymmetric unit contains one $C_{13}H_{11}N$ molecule. All displacement ellipsoids are drawn at the 50% probability level.

Table S1.1. Crystal data and structure refinement for **2**.

Empirical formula	$C_{13}H_{11}N$	
Formula weight	181.23	
Temperature	100(2) K	
Wavelength	0.71073 Å	
Crystal system	Orthorhombic	
Space group	Pbca - D_{2h}^{15} (No. 61)	
Unit cell dimensions	**a** = 13.789(3) Å	**α** = 90.000°
	**b** = 9.154(2) Å	**β** = 90.000°
	**c** = 15.737(3) Å	**γ** = 90.000°
Volume	1986.3(6) $Å^3$	
Z	8	
Density (calculated)	1.212 Mg/m^3	
Absorption coefficient	0.071 mm^{-1}	
F(000)	768	
Crystal size	0.35 x 0.11 x 0.03 mm^3	
Theta range for data collection	3.70 to 30.10°	
Index ranges	$-19 \le h \le 19$, $-12 \le k \le 12$, $-22 \le l \le 20$	
Reflections collected	20632	
Independent reflections	2903 [R_{int} = 0.070]	
Completeness to theta = 30.10°	99.2 %	
Absorption correction	Multi-scan	
Max. and min. transmission	1.000 and 0.978	
Refinement method	Full-matrix least-squares on F^2	
Refinement program	SHELXTL-2000/6.10 (Sheldrick, 2000)	
Data / restraints / parameters	2903 / 0 / 171	
Goodness-of-fit on F^2	1.014	
Final R indices [I>2sigma(I)]	R_1 = 0.058, wR_2 = 0.140	
R indices (all data)	R_1 = 0.094, wR_2 = 0.155	
Largest diff. peak and hole	0.44 and -0.18 $e^-/Å^3$	

$R_1 = \Sigma \,||F_O| - |F_C|| \,/\, \Sigma |F_O|$

$wR_2 = \{ \Sigma [w(F_O^2 - F_C^2)^2] / \Sigma [w(F_O^2)^2] \}^{1/2}$

Table S1.2. Atomic coordinates ($\times 10^4$) and equivalent isotropic displacement parameters ($Å^2 \times 10^3$) for **2**. U(eq) is defined as one third of the trace of the orthogonalized U_{ij} tensor.

	x	y	z	U(eq)
N	1031(1)	1774(1)	3736(1)	35(1)
C(1)	1299(1)	4424(2)	3824(1)	30(1)
C(2)	1109(1)	3068(2)	4198(1)	29(1)
C(3)	1022(1)	3139(1)	5083(1)	28(1)
C(4)	1155(1)	5155(2)	6122(1)	30(1)
C(5)	1295(1)	6585(2)	6402(1)	35(1)
C(6)	1485(1)	7825(2)	5918(1)	36(1)
C(7)	1595(1)	7976(2)	5049(1)	35(1)
C(8)	1532(1)	6915(2)	4416(1)	31(1)
C(9)	1346(1)	5431(2)	4495(1)	27(1)
C(10)	1165(1)	4606(2)	5303(1)	26(1)
C(11)	1422(1)	4715(2)	2898(1)	43(1)
C(12)	976(1)	697(2)	3350(1)	47(1)
C(13)	871(1)	1877(2)	5667(1)	35(1)

Table S1.3. Bond lengths [Å] for **2**.

N-C(12)	1.161(2)	C(6)-C(7)	1.382(2)
N-C(2)	1.394(2)	C(6)-H(6)	0.96(2)
C(1)-C(2)	1.398(2)	C(7)-C(8)	1.394(2)
C(1)-C(9)	1.403(2)	C(7)-H(7)	0.97(2)
C(1)-C(11)	1.491(2)	C(8)-C(9)	1.388(2)
C(2)-C(3)	1.400(2)	C(8)-H(8)	0.96(2)
C(3)-C(10)	1.401(2)	C(9)-C(10)	1.499(2)
C(3)-C(13)	1.490(2)	C(11)-H(11A)	0.95(2)
C(4)-C(10)	1.383(2)	C(11)-H(11B)	0.93(3)
C(4)-C(5)	1.394(2)	C(11)-H(11C)	0.91(2)
C(4)-H(4)	0.97(2)	C(13)-H(13A)	0.99(2)
C(5)-C(6)	1.392(2)	C(13)-H(13B)	0.96(2)
C(5)-H(5)	0.97(2)	C(13)-H(13C)	0.96(2)

Table S1.4. Bond angles [°] for **2**.

C(12)-N-C(2)	179.3(2)	C(9)-C(8)-C(7)	129.0(1)
C(2)-C(1)-C(9)	106.0(1)	C(9)-C(8)-H(8)	115(1)
C(2)-C(1)-C(11)	126.2(1)	C(7)-C(8)-H(8)	116(1)
C(9)-C(1)-C(11)	127.9(1)	C(8)-C(9)-C(1)	125.7(1)
N-C(2)-C(1)	123.4(1)	C(8)-C(9)-C(10)	126.8(1)
N-C(2)-C(3)	123.5(1)	C(1)-C(9)-C(10)	107.5(1)
C(1)-C(2)-C(3)	113.1(1)	C(4)-C(10)-C(3)	125.2(1)
C(2)-C(3)-C(10)	106.2(1)	C(4)-C(10)-C(9)	127.5(1)
C(2)-C(3)-C(13)	126.1(1)	C(3)-C(10)-C(9)	107.2(1)
C(10)-C(3)-C(13)	127.6(1)	C(1)-C(11)-H(11A)	111(2)
C(10)-C(4)-C(5)	129.3(1)	C(1)-C(11)-H(11B)	113(2)
C(10)-C(4)-H(4)	115(1)	H(11A)-C(11)-H(11B)	95(2)
C(5)-C(4)-H(4)	116(1)	C(1)-C(11)-H(11C)	117(2)
C(6)-C(5)-C(4)	128.2(1)	H(11A)-C(11)-H(11C)	112(2)
C(6)-C(5)-H(5)	119(1)	H(11B)-C(11)-H(11C)	107(2)
C(4)-C(5)-H(5)	113(1)	C(3)-C(13)-H(13A)	110(1)
C(7)-C(6)-C(5)	130.1(1)	C(3)-C(13)-H(13B)	110(1)
C(7)-C(6)-H(6)	117(1)	H(13A)-C(13)-H(13B)	111(2)
C(5)-C(6)-H(6)	114(1)	C(3)-C(13)-H(13C)	113(1)
C(6)-C(7)-C(8)	129.1(1)	H(13A)-C(13)-H(13C)	108(2)
C(6)-C(7)-H(7)	116(1)	H(13B)-C(13)-H(13C)	105(2)
C(8)-C(7)-H(7)	115(1)		

Table S1.5. Anisotropic displacement parameters ($Å^2 \times 10^3$) for **2**. The anisotropic displacement factor exponent takes the form: $-2\pi^2[h^2 a^{*2} U_{11} + ... + 2 h k a^* b^* U_{12}]$

	U_{11}	U_{22}	U_{33}	U_{23}	U_{13}	U_{12}
N	36(1)	34(1)	34(1)	-6(1)	-2(1)	2(1)
C(1)	28(1)	35(1)	26(1)	1(1)	0(1)	2(1)
C(2)	28(1)	29(1)	30(1)	-4(1)	-2(1)	2(1)
C(3)	28(1)	25(1)	30(1)	1(1)	-1(1)	2(1)
C(4)	32(1)	31(1)	27(1)	2(1)	0(1)	2(1)
C(5)	38(1)	36(1)	31(1)	-6(1)	-1(1)	2(1)
C(6)	38(1)	28(1)	42(1)	-7(1)	-1(1)	0(1)
C(7)	32(1)	26(1)	45(1)	3(1)	0(1)	-2(1)
C(8)	29(1)	30(1)	33(1)	7(1)	1(1)	0(1)
C(9)	26(1)	29(1)	26(1)	2(1)	0(1)	1(1)
C(10)	24(1)	26(1)	26(1)	2(1)	1(1)	2(1)
C(11)	50(1)	52(1)	26(1)	2(1)	0(1)	-3(1)
C(12)	54(1)	45(1)	42(1)	-10(1)	0(1)	-1(1)
C(13)	44(1)	26(1)	36(1)	6(1)	-1(1)	1(1)

Table S1.6. Torsion angles [°] for **2**.

C(12)-N-C(2)-C(1)	74(13)	C(7)-C(8)-C(9)-C(10)	0.0(2)
C(12)-N-C(2)-C(3)	-105(13)	C(2)-C(1)-C(9)-C(8)	179.2(1)
C(9)-C(1)-C(2)-N	-178.6(1)	C(11)-C(1)-C(9)-C(8)	-0.9(2)
C(11)-C(1)-C(2)-N	1.6(2)	C(2)-C(1)-C(9)-C(10)	-0.4(1)
C(9)-C(1)-C(2)-C(3)	0.4(2)	C(11)-C(1)-C(9)-C(10)	179.5(1)
C(11)-C(1)-C(2)-C(3)	-179.5(1)	C(5)-C(4)-C(10)-C(3)	179.5(1)
N-C(2)-C(3)-C(10)	178.7(1)	C(5)-C(4)-C(10)-C(9)	0.7(2)
C(1)-C(2)-C(3)-C(10)	-0.3(2)	C(2)-C(3)-C(10)-C(4)	-179.0(1)
N-C(2)-C(3)-C(13)	2.2(2)	C(13)-C(3)-C(10)-C(4)	-2.5(2)
C(1)-C(2)-C(3)-C(13)	-176.8(1)	C(2)-C(3)-C(10)-C(9)	0.0(1)
C(10)-C(4)-C(5)-C(6)	0.0(2)	C(13)-C(3)-C(10)-C(9)	176.5(1)
C(4)-C(5)-C(6)-C(7)	-0.9(3)	C(8)-C(9)-C(10)-C(4)	-0.4(2)
C(5)-C(6)-C(7)-C(8)	1.0(3)	C(1)-C(9)-C(10)-C(4)	179.2(1)
C(6)-C(7)-C(8)-C(9)	-0.4(2)	C(8)-C(9)-C(10)-C(3)	-179.4(1)
C(7)-C(8)-C(9)-C(1)	-179.5(1)	C(1)-C(9)-C(10)-C(3)	0.2(1)

A2. X-ray Crystallographic Characterization of 6.

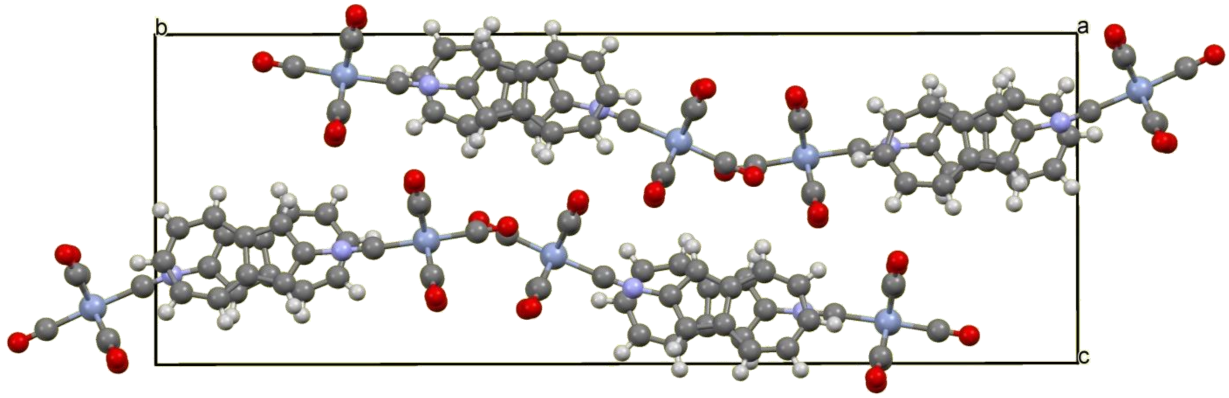

Figure S2. Packing diagram of **6** (view along the a axis).

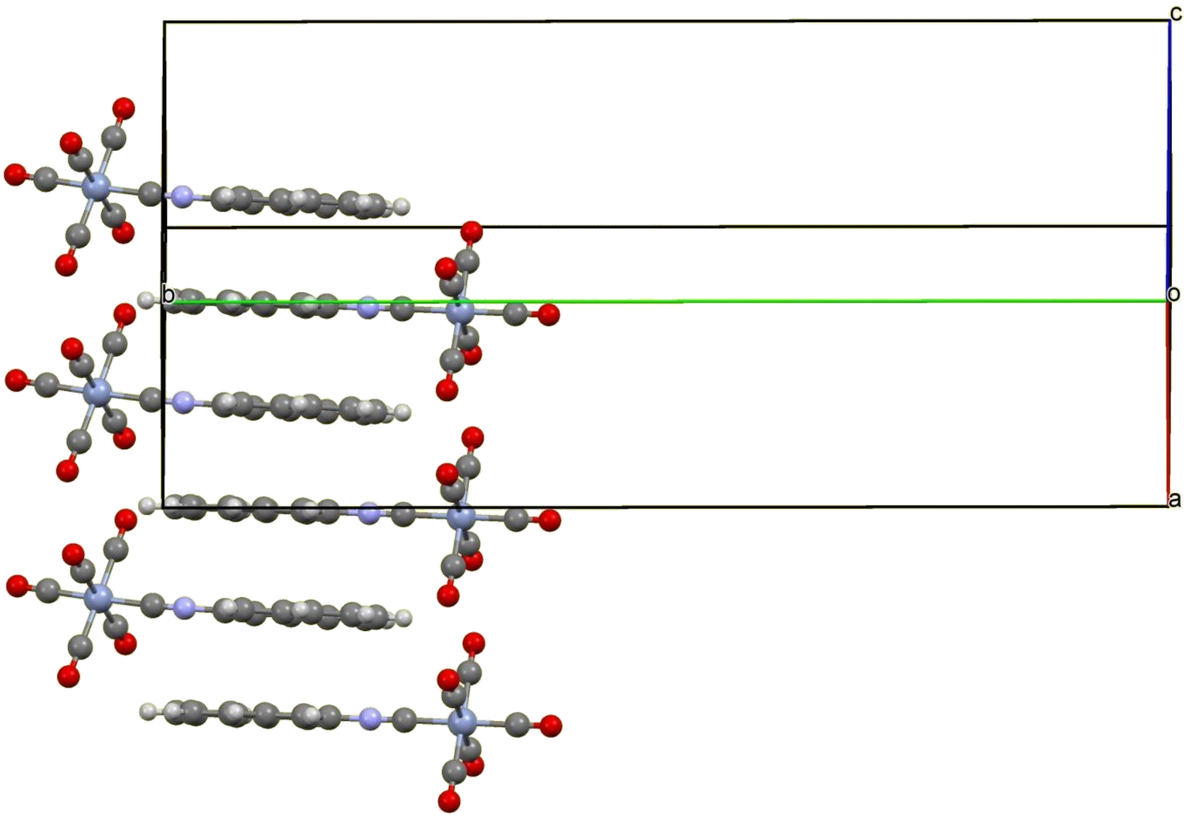

Figure S3. The π-stacked head-to-tail dimers that form 1D columns in the structure of **6**.

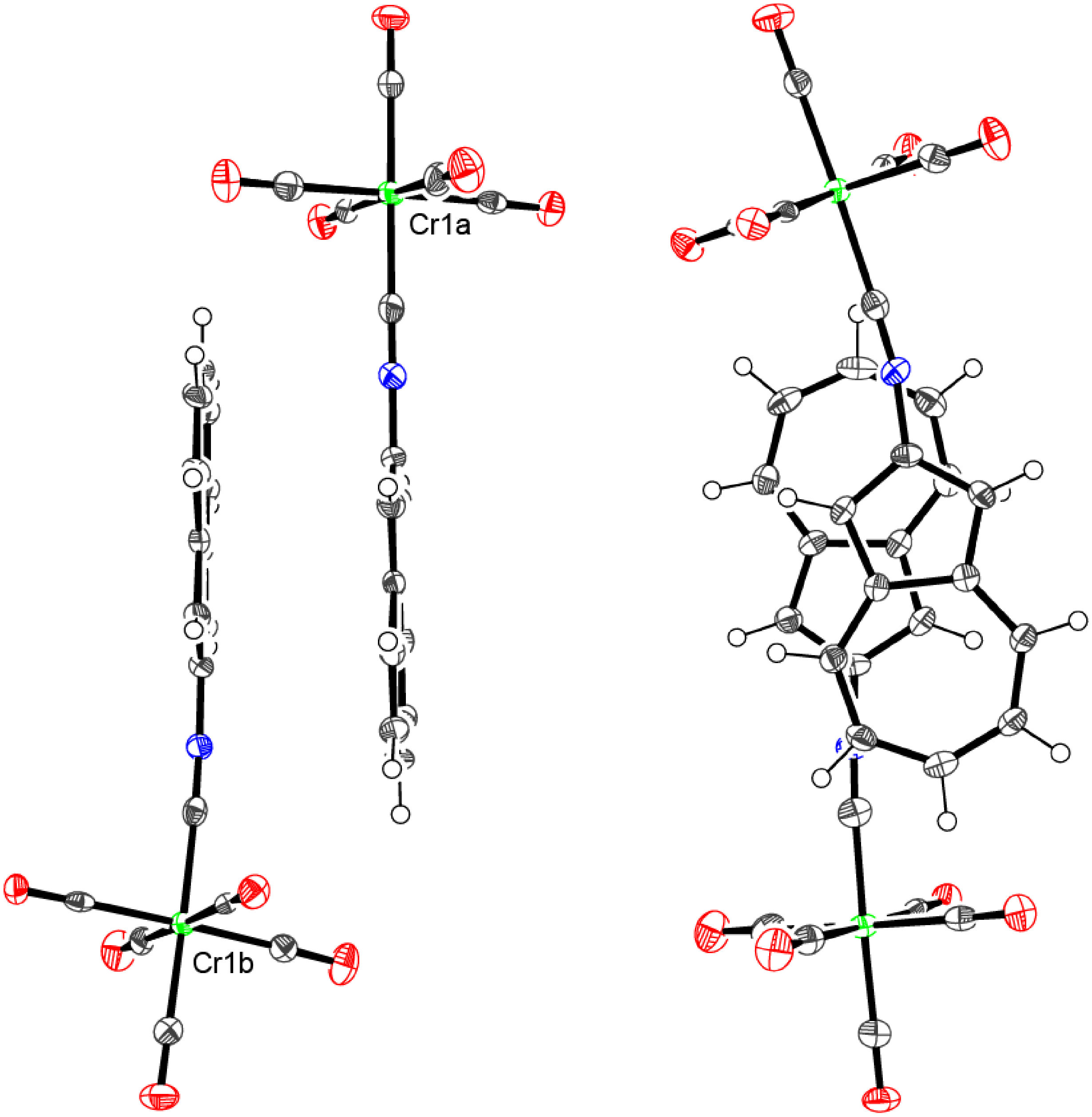

Figure S4. Two orthogonal views of the π-stacked head-to-tail pair in the structure of **6.**

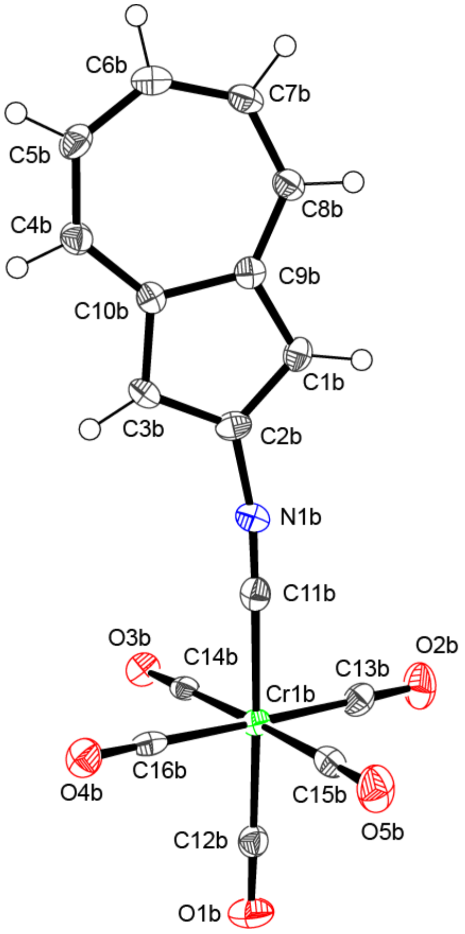

Figure S5. One of two crystallographically independent molecules in the solid-state structure of **6** (the other molecule is shown in Figure 5a of the main article). All thermal ellipsoids are drawn at the 50% probability level.

Table S2.1. Crystal data and structure refinement for **6**.

Chemical formula	$C_{16}H_7CrNO_5$	
Formula weight	345.23 g/mol	
Temperature	100(2) K	
Wavelength	0.71073 Å	
Crystal size	0.085 x 0.099 x 0.246 mm	
Crystal habit	lustrous intense black-red needle	
Crystal system	Monoclinic	
Space group	P 1 21/c 1	
Unit cell dimensions	a = 7.3181(8) Å	α = 90°
	b = 33.828(4) Å	β = 115.582(5)°
	c = 13.3970(12) Å	γ = 90°
Volume	2991.4(6) $Å^3$	
Z	8	
Density (calculated)	1.533 g/cm^3	
Absorption coefficient	0.789 mm^{-1}	
F(000)	1392	
Theta range for data collection	1.20 to 26.41°	
Index ranges	-9<=h<=9, -42<=k<=42, -16<=l<=16	
Reflections collected	29797	
Independent reflections	6118 [R(int) = 0.0956]	
Coverage of independent reflections	99.6%	
Absorption correction	multi-scan	
Max. and min. transmission	0.9360 and 0.8300	
Refinement method	Full-matrix least-squares on F^2	
Refinement program	SHELXL-2014/7 (Sheldrick, 2014)	
Function minimized	Σ w(Fo2 - Fc2)^2	
Data / restraints / parameters	6118 / 0 / 415	
Goodness-of-fit on F^2	1.004	
Final R indices	4053 data; I>2σ(I)	R1 = 0.0522, wR2 = 0.1118
	all data	R1 = 0.0890, wR2 = 0.1303

Weighting scheme	$w=1/[\sigma^2(F_o^2)+(0.0536P)^2]$ where $P=(F_o^2+2F_c^2)/3$
Absolute structure parameter	0.0(1)
Largest diff. peak and hole	0.605 and -0.380 eÅ^{-3}
R.M.S. deviation from mean	0.090 eÅ^{-3}

Table S2.2. Atomic coordinates and equivalent isotropic displacement parameters ($Å^2$) for **6**. U(eq) is defined as one third of the trace of the orthogonalized U_{ij} tensor.

	x/a	y/b	z/c	U(eq)
C1A	0.2014(5)	0.13672(10)	0.4231(3)	0.0238(8)
C2A	0.0763(5)	0.16129(9)	0.3367(3)	0.0234(8)
C3A	0.9503(5)	0.14002(10)	0.2443(3)	0.0232(8)
C4A	0.9017(5)	0.06892(10)	0.1998(3)	0.0239(8)
C5A	0.9346(6)	0.02836(10)	0.2182(3)	0.0293(9)
C6A	0.0679(6)	0.00937(10)	0.3122(4)	0.0324(9)
C7A	0.2065(6)	0.02471(11)	0.4133(3)	0.0296(9)
C8A	0.2437(5)	0.06396(10)	0.4443(3)	0.0262(8)
C9A	0.1550(5)	0.09786(10)	0.3848(3)	0.0204(8)
C10A	0.9922(5)	0.10010(10)	0.2700(3)	0.0207(8)
C11A	0.0834(5)	0.23647(11)	0.3520(3)	0.0274(9)
C12A	0.1262(5)	0.34901(10)	0.4009(3)	0.0260(8)
C13A	0.3771(6)	0.28634(10)	0.4804(3)	0.0236(8)
C14A	0.1856(5)	0.30077(10)	0.2590(3)	0.0279(9)
C15A	0.0171(5)	0.28649(9)	0.4890(3)	0.0227(8)
C16A	0.8262(6)	0.30005(11)	0.2685(3)	0.0300(9)
C1B	0.6773(5)	0.07576(9)	0.3932(3)	0.0213(8)
C2B	0.5386(5)	0.05850(9)	0.2945(3)	0.0218(8)
C3B	0.4211(5)	0.08717(9)	0.2189(3)	0.0208(8)
C4B	0.4053(5)	0.16006(10)	0.2212(3)	0.0232(8)
C5B	0.4586(5)	0.19773(10)	0.2644(3)	0.0253(8)
C6B	0.6063(5)	0.20859(10)	0.3683(3)	0.0247(8)
C7B	0.7369(5)	0.18557(10)	0.4549(3)	0.0234(8)
C8B	0.7571(5)	0.14463(10)	0.4604(3)	0.0220(8)
C9B	0.6494(5)	0.11659(10)	0.3812(3)	0.0188(7)
C10B	0.4846(5)	0.12383(9)	0.2695(3)	0.0184(7)
C11B	0.4859(5)	0.98620(10)	0.2370(3)	0.0220(8)
C12B	0.3816(5)	0.88165(10)	0.1073(3)	0.0236(8)
C13B	0.7006(6)	0.91688(10)	0.2707(3)	0.0240(8)

	x/a	y/b	z/c	U(eq)
C14B	0.5276(5)	0.94917(9)	0.0680(3)	0.0199(7)
C15B	0.3348(5)	0.91401(10)	0.2744(3)	0.0229(8)
C16B	0.1636(6)	0.94862(9)	0.0765(3)	0.0209(8)
Cr1A	0.10259(8)	0.29424(2)	0.37487(5)	0.02245(16)
Cr1B	0.43305(8)	0.93212(2)	0.17284(4)	0.01833(15)
N1A	0.0780(5)	0.20244(9)	0.3440(3)	0.0281(7)
N1B	0.5187(4)	0.01799(8)	0.2711(2)	0.0218(6)
O1A	0.1406(4)	0.38245(7)	0.4163(2)	0.0344(6)
O2A	0.5390(4)	0.28112(7)	0.5433(2)	0.0326(6)
O3A	0.2340(4)	0.30528(8)	0.1895(2)	0.0401(7)
O4A	0.6629(4)	0.30378(8)	0.2056(2)	0.0435(7)
O5A	0.9693(4)	0.28145(7)	0.5587(2)	0.0304(6)
O1B	0.3496(4)	0.85100(7)	0.0671(2)	0.0379(7)
O2B	0.8617(4)	0.90780(8)	0.3307(2)	0.0377(7)
O3B	0.5838(4)	0.95900(7)	0.0040(2)	0.0247(6)
O4B	0.0024(4)	0.95801(7)	0.0192(2)	0.0295(6)
O5B	0.2728(4)	0.90250(8)	0.3339(2)	0.0381(7)

Table S2.3. Bond lengths [Å] for **6**.

C1A-C2A	1.398(5)	C1A-C9A	1.399(4)
C1A-H1A	0.95	C2A-C3A	1.383(5)
C2A-N1A	1.395(4)	C3A-C10A	1.395(5)
C3A-H3A	0.95	C4A-C10A	1.377(5)
C4A-C5A	1.397(5)	C4A-H4A	0.95
C5A-C6A	1.374(5)	C5A-H5A	0.95
C6A-C7A	1.395(5)	C6A-H6A	0.95
C7A-C8A	1.382(5)	C7A-H7A	0.95
C8A-C9A	1.387(5)	C8A-H8A	0.95
C9A-C10A	1.486(5)	C11A-N1A	1.155(4)
C11A-Cr1A	1.974(4)	C12A-O1A	1.146(4)
C12A-Cr1A	1.879(4)	C13A-O2A	1.133(4)
C13A-Cr1A	1.911(4)	C14A-O3A	1.141(4)
C14A-Cr1A	1.908(4)	C15A-O5A	1.142(4)
C15A-Cr1A	1.904(4)	C16A-O4A	1.133(4)
C16A-Cr1A	1.916(4)	C1B-C9B	1.395(4)
C1B-C2B	1.401(5)	C1B-H1C	0.95
C2B-C3B	1.397(5)	C2B-N1B	1.399(4)
C3B-C10B	1.393(4)	C3B-H3C	0.95
C4B-C5B	1.384(5)	C4B-C10B	1.391(4)
C4B-H4C	0.95	C5B-C6B	1.393(5)
C5B-H5C	0.95	C6B-C7B	1.381(5)
C6B-H6C	0.95	C7B-C8B	1.391(4)
C7B-H7C	0.95	C8B-C9B	1.388(4)
C8B-H8C	0.95	C9B-C10B	1.481(5)
C11B-N1B	1.153(4)	C11B-Cr1B	1.987(4)
C12B-O1B	1.145(4)	C12B-Cr1B	1.882(4)
C13B-O2B	1.146(4)	C13B-Cr1B	1.898(4)
C14B-O3B	1.149(4)	C14B-Cr1B	1.903(4)
C15B-O5B	1.143(4)	C15B-Cr1B	1.894(4)
C16B-O4B	1.140(4)	C16B-Cr1B	1.915(4)

Table S2.4. Bond angles [°] for **6.**

C2A-C1A-C9A	106.7(3)	C2A-C1A-H1A	126.7
C9A-C1A-H1A	126.7	C3A-C2A-N1A	124.4(3)
C3A-C2A-C1A	112.1(3)	N1A-C2A-C1A	123.5(3)
C2A-C3A-C10A	107.0(3)	C2A-C3A-H3A	126.5
C10A-C3A-H3A	126.5	C10A-C4A-C5A	129.5(4)
C10A-C4A-H4A	115.2	C5A-C4A-H4A	115.2
C6A-C5A-C4A	128.4(4)	C6A-C5A-H5A	115.8
C4A-C5A-H5A	115.8	C5A-C6A-C7A	130.3(3)
C5A-C6A-H6A	114.9	C7A-C6A-H6A	114.9
C8A-C7A-C6A	127.9(4)	C8A-C7A-H7A	116.0
C6A-C7A-H7A	116.0	C7A-C8A-C9A	129.7(4)
C7A-C8A-H8A	115.1	C9A-C8A-H8A	115.1
C8A-C9A-C1A	126.0(3)	C8A-C9A-C10A	127.1(3)
C1A-C9A-C10A	106.9(3)	C4A-C10A-C3A	125.7(3)
C4A-C10A-C9A	127.0(3)	C3A-C10A-C9A	107.3(3)
N1A-C11A-Cr1A	176.7(4)	O1A-C12A-Cr1A	179.6(3)
O2A-C13A-Cr1A	179.0(3)	O3A-C14A-Cr1A	178.9(3)
O5A-C15A-Cr1A	178.6(3)	O4A-C16A-Cr1A	179.5(3)
C9B-C1B-C2B	107.1(3)	C9B-C1B-H1C	126.5
C2B-C1B-H1C	126.5	C3B-C2B-N1B	123.1(3)
C3B-C2B-C1B	111.3(3)	N1B-C2B-C1B	125.6(3)
C10B-C3B-C2B	107.1(3)	C10B-C3B-H3C	126.5
C2B-C3B-H3C	126.5	C5B-C4B-C10B	129.1(3)
C5B-C4B-H4C	115.4	C10B-C4B-H4C	115.4
C4B-C5B-C6B	128.1(3)	C4B-C5B-H5C	115.9
C6B-C5B-H5C	115.9	C7B-C6B-C5B	130.3(3)
C7B-C6B-H6C	114.8	C5B-C6B-H6C	114.8
C6B-C7B-C8B	128.7(3)	C6B-C7B-H7C	115.7
C8B-C7B-H7C	115.7	C9B-C8B-C7B	128.9(3)
C9B-C8B-H8C	115.6	C7B-C8B-H8C	115.6
C8B-C9B-C1B	125.6(3)	C8B-C9B-C10B	127.3(3)
C1B-C9B-C10B	107.2(3)	C4B-C10B-C3B	125.0(3)

C4B-C10B-C9B	127.6(3)	C3B-C10B-C9B	107.4(3)
N1B-C11B-Cr1B	177.4(3)	O1B-C12B-Cr1B	179.7(4)
O2B-C13B-Cr1B	179.3(3)	O3B-C14B-Cr1B	179.1(3)
O5B-C15B-Cr1B	178.5(3)	O4B-C16B-Cr1B	179.2(3)
C12A-Cr1A-C15A	91.29(14)	C12A-Cr1A-C14A	90.04(15)
C15A-Cr1A-C14A	178.59(15)	C12A-Cr1A-C13A	91.01(15)
C15A-Cr1A-C13A	89.52(15)	C14A-Cr1A-C13A	90.90(15)
C12A-Cr1A-C16A	91.14(15)	C15A-Cr1A-C16A	90.22(15)
C14A-Cr1A-C16A	89.31(16)	C13A-Cr1A-C16A	177.84(15)
C12A-Cr1A-C11A	178.34(17)	C15A-Cr1A-C11A	87.83(14)
C14A-Cr1A-C11A	90.85(15)	C13A-Cr1A-C11A	87.58(15)
C16A-Cr1A-C11A	90.26(15)	C12B-Cr1B-C15B	88.85(14)
C12B-Cr1B-C13B	90.84(15)	C15B-Cr1B-C13B	90.83(15)
C12B-Cr1B-C14B	89.68(14)	C15B-Cr1B-C14B	178.41(14)
C13B-Cr1B-C14B	89.80(14)	C12B-Cr1B-C16B	90.56(14)
C15B-Cr1B-C16B	88.76(14)	C13B-Cr1B-C16B	178.53(15)
C14B-Cr1B-C16B	90.65(14)	C12B-Cr1B-C11B	178.03(14)
C15B-Cr1B-C11B	92.89(14)	C13B-Cr1B-C11B	90.08(14)
C14B-Cr1B-C11B	88.58(13)	C16B-Cr1B-C11B	88.54(13)
C11A-N1A-C2A	178.5(4)	C11B-N1B-C2B	170.5(3)

Table S2.5. Anisotropic displacement parameters ($Å^2$) for **6**. The anisotropic displacement factor exponent takes the form: $-2\pi^2[h^2a^{*2}U_{11} + ... + 2 h k a^* b^* U_{12}]$.

	U_{11}	U_{22}	U_{33}	U_{23}	U_{13}	U_{12}
C1A	0.023(2)	0.0271(19)	0.021(2)	-0.0063(16)	0.0095(17)	-0.0063(15)
C2A	0.026(2)	0.0169(17)	0.033(2)	-0.0035(16)	0.0188(18)	-0.0033(15)
C3A	0.021(2)	0.0231(18)	0.025(2)	0.0000(15)	0.0090(16)	0.0013(15)
C4A	0.023(2)	0.029(2)	0.0209(19)	-0.0023(16)	0.0109(16)	-0.0004(16)
C5A	0.029(2)	0.026(2)	0.037(2)	-0.0128(18)	0.0176(19)	-0.0109(17)
C6A	0.038(2)	0.0160(18)	0.053(3)	-0.0014(18)	0.029(2)	0.0002(17)
C7A	0.027(2)	0.028(2)	0.035(2)	0.0082(17)	0.0144(19)	0.0025(17)
C8A	0.021(2)	0.033(2)	0.026(2)	0.0005(17)	0.0119(17)	-0.0007(16)
C9A	0.0170(19)	0.0257(18)	0.0203(19)	-0.0039(15)	0.0099(16)	-0.0029(14)
C10A	0.022(2)	0.0213(17)	0.024(2)	-0.0054(15)	0.0144(16)	-0.0037(15)
C11A	0.023(2)	0.029(2)	0.035(2)	-0.0028(17)	0.0171(18)	-0.0028(16)
C12A	0.024(2)	0.025(2)	0.033(2)	0.0001(17)	0.0163(18)	-0.0001(16)
C13A	0.028(2)	0.0195(18)	0.030(2)	-0.0012(16)	0.0191(19)	-0.0018(16)
C14A	0.018(2)	0.026(2)	0.036(2)	-0.0024(17)	0.0090(18)	0.0000(15)
C15A	0.0163(19)	0.0166(17)	0.029(2)	-0.0052(15)	0.0037(17)	-0.0017(14)
C16A	0.026(2)	0.032(2)	0.036(2)	-0.0070(18)	0.017(2)	-0.0079(17)
C1B	0.0204(19)	0.0225(18)	0.0218(19)	0.0038(15)	0.0098(16)	0.0047(14)
C2B	0.023(2)	0.0178(17)	0.028(2)	-0.0024(15)	0.0147(17)	0.0003(14)
C3B	0.0198(19)	0.0235(18)	0.0178(18)	-0.0054(15)	0.0070(15)	-0.0031(15)
C4B	0.025(2)	0.0235(18)	0.0206(19)	-0.0023(15)	0.0096(16)	0.0022(15)
C5B	0.029(2)	0.0203(18)	0.027(2)	0.0038(16)	0.0136(18)	0.0071(15)
C6B	0.027(2)	0.0202(18)	0.033(2)	-0.0060(16)	0.0181(18)	-0.0045(16)
C7B	0.025(2)	0.0236(18)	0.022(2)	-0.0058(16)	0.0107(17)	-0.0076(16)
C8B	0.022(2)	0.0245(18)	0.0195(19)	-0.0018(15)	0.0090(16)	-0.0043(15)
C9B	0.0184(19)	0.0227(17)	0.0205(19)	0.0010(15)	0.0132(16)	-0.0012(14)
C10B	0.0180(19)	0.0227(18)	0.0171(18)	-0.0002(14)	0.0100(15)	-0.0003(14)
C11B	0.0151(19)	0.027(2)	0.024(2)	0.0037(16)	0.0084(16)	0.0009(15)
C12B	0.021(2)	0.0238(19)	0.024(2)	0.0030(16)	0.0077(17)	0.0011(16)
C13B	0.029(2)	0.0227(18)	0.026(2)	0.0036(16)	0.0168(18)	0.0017(16)

	U_{11}	U_{22}	U_{33}	U_{23}	U_{13}	U_{12}
C14B	0.0139(18)	0.0128(16)	0.026(2)	-0.0032(15)	0.0023(16)	0.0019(14)
C15B	0.020(2)	0.0249(19)	0.021(2)	-0.0037(16)	0.0062(17)	-0.0016(15)
C16B	0.026(2)	0.0142(17)	0.028(2)	-0.0033(15)	0.0173(18)	-0.0053(15)
Cr1A	0.0221(3)	0.0177(3)	0.0293(3)	-0.0036(2)	0.0128(3)	-0.0015(2)
Cr1B	0.0182(3)	0.0167(3)	0.0207(3)	-0.0006(2)	0.0089(2)	-0.0004(2)
N1A	0.0294(19)	0.0231(17)	0.038(2)	-0.0054(14)	0.0205(16)	-0.0025(13)
N1B	0.0209(17)	0.0223(16)	0.0229(16)	-0.0042(13)	0.0102(14)	-0.0032(13)
O1A	0.0390(17)	0.0188(14)	0.0457(17)	-0.0039(12)	0.0184(14)	-0.0004(12)
O2A	0.0272(16)	0.0402(16)	0.0326(16)	0.0018(13)	0.0150(13)	0.0037(13)
O3A	0.0380(18)	0.0525(18)	0.0375(17)	-0.0006(14)	0.0236(15)	-0.0012(14)
O4A	0.0255(17)	0.057(2)	0.0449(19)	-0.0048(15)	0.0123(15)	-0.0013(14)
O5A	0.0251(15)	0.0371(15)	0.0309(16)	-0.0005(12)	0.0140(13)	-0.0043(12)
O1B	0.0437(18)	0.0222(14)	0.0481(18)	-0.0084(13)	0.0200(15)	-0.0052(12)
O2B	0.0236(16)	0.0490(18)	0.0344(17)	0.0113(14)	0.0069(13)	0.0092(13)
O3B	0.0238(14)	0.0270(13)	0.0283(14)	0.0009(11)	0.0159(12)	0.0007(11)
O4B	0.0193(15)	0.0312(14)	0.0363(16)	0.0056(12)	0.0105(13)	0.0034(11)
O5B	0.0363(17)	0.0513(18)	0.0293(16)	0.0085(13)	0.0167(14)	-0.0040(13)

Table S2.6. Torsion angles [°] for **6.**

C9A-C1A-C2A-C3A	0.3(4)	C9A-C1A-C2A-N1A	179.6(3)
N1A-C2A-C3A-C10A	-179.2(3)	C1A-C2A-C3A-C10A	0.1(4)
C10A-C4A-C5A-C6A	-0.3(6)	C4A-C5A-C6A-C7A	-0.7(7)
C5A-C6A-C7A-C8A	0.2(7)	C6A-C7A-C8A-C9A	0.3(6)
C7A-C8A-C9A-C1A	179.6(3)	C7A-C8A-C9A-C10A	0.4(6)
C2A-C1A-C9A-C8A	-179.9(3)	C2A-C1A-C9A-C10A	-0.5(4)
C5A-C4A-C10A-C3A	179.7(3)	C5A-C4A-C10A-C9A	1.6(6)
C2A-C3A-C10A-C4A	-178.9(3)	C2A-C3A-C10A-C9A	-0.5(4)
C8A-C9A-C10A-C4A	-1.6(5)	C1A-C9A-C10A-C4A	179.1(3)
C8A-C9A-C10A-C3A	180.0(3)	C1A-C9A-C10A-C3A	0.6(4)
C9B-C1B-C2B-C3B	-0.2(4)	C9B-C1B-C2B-N1B	178.8(3)
N1B-C2B-C3B-C10B	-179.0(3)	C1B-C2B-C3B-C10B	0.0(4)
C10B-C4B-C5B-C6B	-0.2(6)	C4B-C5B-C6B-C7B	-0.1(6)
C5B-C6B-C7B-C8B	0.8(6)	C6B-C7B-C8B-C9B	-1.0(6)
C7B-C8B-C9B-C1B	-179.4(3)	C7B-C8B-C9B-C10B	0.2(6)
C2B-C1B-C9B-C8B	180.0(3)	C2B-C1B-C9B-C10B	0.3(3)
C5B-C4B-C10B-C3B	-179.7(3)	C5B-C4B-C10B-C9B	-0.3(6)
C2B-C3B-C10B-C4B	179.8(3)	C2B-C3B-C10B-C9B	0.2(4)
C8B-C9B-C10B-C4B	0.4(5)	C1B-C9B-C10B-C4B	-179.9(3)
C8B-C9B-C10B-C3B	180.0(3)	C1B-C9B-C10B-C3B	-0.3(3)

A3. X-ray Crystallographic Characterization of 7.

Comment

All H-atoms were found on the electron difference map and refined isotropically.

Table S3.1. Crystal data and structure refinement for **7**.

Chemical formula	$C_{18}H_{11}CrNO_5$	
Formula weight	373.28 g/mol	
Temperature	100(2) K	
Wavelength	0.71073 Å	
Crystal size	0.134 x 0.324 x 0.502 mm	
Crystal habit	clear intense black-green plate	
Crystal system	monoclinic	
Space group	P 1 21/c 1	
Unit cell dimensions	a = 14.0814(9) Å	α = 90°
	b = 16.5225(10) Å	β = 103.8080(10)°
	c = 7.3496(5) Å	γ = 90°
Volume	1660.54(18) $Å^3$	
Z	4	
Density (calculated)	1.493 g/cm^3	
Absorption coefficient	0.717 mm^{-1}	
F(000)	760	
Theta range for data collection	1.49 to 29.52°	
Index ranges	-19<=h<=19, -22<=k<=22, -10<=l<=10	
Reflections collected	23401	
Independent reflections	4610 [R(int) = 0.0210]	
Refinement method	Full-matrix least-squares on F^2	
Refinement program	SHELXL-2014/7 (Sheldrick, 2014)	
Function minimized	Σ w(F_o^2 - F_c^2)^2	
Data / restraints / parameters	4610 / 0 / 270	
Goodness-of-fit on F^2	1.068	
Δ/σ_{max}	0.001	
Final R indices	4085 data; I>2σ(I)	R1 = 0.0340, wR2 = 0.0902
	all data	R1 = 0.0385, wR2 = 0.0933
Weighting scheme	w=1/[σ^2(F_o^2)+(0.0563P)^2+0.5299P] where P=(F_o^2+2F_c^2)/3	
Largest diff. peak and hole	0.522 and -0.316 e$Å^{-3}$	
R.M.S. deviation from mean	0.069 e$Å^{-3}$	

Table S3.2. Atomic coordinates and equivalent isotropic displacement parameters (Å^2) for **7**. U(eq) is defined as one third of the trace of the orthogonalized U_{ij} tensor.

	**x/a**	**y/b**	**z/c**	**U(eq)**
C1	0.78241(10)	0.28415(8)	0.55778(18)	0.0192(2)
C2	0.72086(10)	0.34979(8)	0.57414(17)	0.0187(2)
C3	0.62851(9)	0.32524(8)	0.59396(17)	0.0184(2)
C4	0.55461(10)	0.19101(9)	0.62068(18)	0.0210(3)
C5	0.55097(11)	0.10653(9)	0.6308(2)	0.0251(3)
C6	0.62156(12)	0.05052(9)	0.6119(2)	0.0276(3)
C7	0.71415(12)	0.06213(9)	0.5781(2)	0.0270(3)
C8	0.76172(11)	0.13483(9)	0.56001(19)	0.0233(3)
C9	0.72862(9)	0.21391(8)	0.57005(17)	0.0184(2)
C10	0.63054(9)	0.23982(8)	0.59512(17)	0.0175(2)
C11	0.88513(11)	0.29152(10)	0.5358(2)	0.0271(3)
C12	0.54682(11)	0.37964(9)	0.6135(2)	0.0244(3)
C13	0.77530(10)	0.49677(8)	0.57470(18)	0.0200(2)
C14	0.86384(10)	0.71859(8)	0.58678(18)	0.0203(3)
C15	0.90101(10)	0.57629(8)	0.41688(19)	0.0215(3)
C16	0.73384(10)	0.63567(9)	0.7344(2)	0.0227(3)
C17	0.72070(10)	0.63880(9)	0.36174(19)	0.0219(3)
C18	0.92555(10)	0.59021(8)	0.79273(19)	0.0206(2)
Cr1	0.82010(2)	0.61051(2)	0.57780(3)	0.01653(8)
N1	0.74958(9)	0.42971(7)	0.57376(16)	0.0220(2)
O1	0.89010(8)	0.78423(6)	0.59503(16)	0.0294(2)
O2	0.94727(8)	0.55335(7)	0.31981(16)	0.0301(2)
O3	0.68100(8)	0.65000(8)	0.82534(16)	0.0343(3)
O4	0.66343(8)	0.65902(7)	0.23281(15)	0.0314(2)
O5	0.99024(8)	0.58241(7)	0.91814(15)	0.0280(2)

Table S3.3. Bond lengths [Å] for **7**.

C1-C9	1.4006(18)	C1-C2	1.4112(19)
C1-C11	1.4981(19)	C2-N1	1.3812(17)
C2-C3	1.4027(18)	C3-C10	1.4116(18)
C3-C12	1.4935(19)	C4-C10	1.3873(18)
C4-C5	1.399(2)	C4-H4	0.99(2)
C5-C6	1.389(2)	C5-H5	0.96(2)
C6-C7	1.397(2)	C6-H6	0.99(2)
C7-C8	1.397(2)	C7-H7	0.89(2)
C8-C9	1.3950(19)	C8-H8	0.95(2)
C9-C10	1.4988(18)	C11-H11A	0.98(3)
C11-H11B	0.98(2)	C11-H11C	0.91(2)
C12-H12A	0.92(3)	C12-H12B	0.94(3)
C12-H12C	0.97(2)	C13-N1	1.1651(18)
C13-Cr1	1.9808(14)	C14-O1	1.1428(18)
C14-Cr1	1.8849(14)	C15-O2	1.1396(18)
C15-Cr1	1.9131(14)	C16-O3	1.1373(18)
C16-Cr1	1.9081(14)	C17-O4	1.1380(18)
C17-Cr1	1.9075(14)	C18-O5	1.1380(18)
C18-Cr1	1.9218(14)		

Table S3.4. Bond angles [°] for **7.**

C9-C1-C2	106.19(11)	C9-C1-C11	128.69(13)
C2-C1-C11	125.11(12)	N1-C2-C3	123.77(13)
N1-C2-C1	123.26(12)	C3-C2-C1	112.96(12)
C2-C3-C10	105.78(11)	C2-C3-C12	126.18(13)
C10-C3-C12	128.03(12)	C10-C4-C5	128.77(14)
C10-C4-H4	118.3(12)	C5-C4-H4	113.0(12)
C6-C5-C4	128.54(14)	C6-C5-H5	116.5(12)
C4-C5-H5	115.0(12)	C5-C6-C7	130.28(14)
C5-C6-H6	114.2(11)	C7-C6-H6	115.5(11)
C8-C7-C6	128.59(14)	C8-C7-H7	114.3(13)
C6-C7-H7	117.1(12)	C9-C8-C7	128.78(14)
C9-C8-H8	109.7(13)	C7-C8-H8	121.5(13)
C8-C9-C1	125.46(13)	C8-C9-C10	127.11(12)
C1-C9-C10	107.43(11)	C4-C10-C3	124.53(12)
C4-C10-C9	127.82(12)	C3-C10-C9	107.62(11)
C1-C11-H11A	113.9(15)	C1-C11-H11B	111.4(12)
H11A-C11-H11B	106.7(19)	C1-C11-H11C	110.4(13)
H11A-C11-H11C	108.(2)	H11B-C11-H11C	105.6(18)
C3-C12-H12A	110.1(18)	C3-C12-H12B	113.9(15)
H12A-C12-H12B	107.(2)	C3-C12-H12C	108.0(14)
H12A-C12-H12C	110.(2)	H12B-C12-H12C	108.(2)
N1-C13-Cr1	179.51(13)	O1-C14-Cr1	178.90(12)
O2-C15-Cr1	177.49(12)	O3-C16-Cr1	178.69(13)
O4-C17-Cr1	176.87(13)	O5-C18-Cr1	176.01(12)
C14-Cr1-C17	88.04(6)	C14-Cr1-C16	91.28(6)
C17-Cr1-C16	90.15(6)	C14-Cr1-C15	93.66(6)
C17-Cr1-C15	89.13(6)	C16-Cr1-C15	174.98(6)
C14-Cr1-C18	87.40(6)	C17-Cr1-C18	175.28(6)
C16-Cr1-C18	91.16(6)	C15-Cr1-C18	89.96(6)
C14-Cr1-C13	178.68(6)	C17-Cr1-C13	93.13(6)
C16-Cr1-C13	88.10(6)	C15-Cr1-C13	86.99(6)
C18-Cr1-C13	91.45(6)	C13-N1-C2	178.93(15)

Table S3.5. Anisotropic displacement parameters (Å^2) for **7**. The anisotropic displacement factor exponent takes the form: $-2\pi^2[h^2a^{*2}U_{11} + ... + 2 h k a^* b^* U_{12}]$.

	**U11**	**U22**	**U33**	**U23**	**U13**	**U12**
C1	0.0167(6)	0.0214(6)	0.0187(6)	0.0005(5)	0.0025(4)	-0.0028(5)
C2	0.0213(6)	0.0180(6)	0.0159(5)	0.0007(4)	0.0028(4)	-0.0038(5)
C3	0.0195(6)	0.0197(6)	0.0156(5)	0.0004(4)	0.0037(4)	-0.0012(5)
C4	0.0193(6)	0.0254(7)	0.0175(6)	-0.0003(5)	0.0029(5)	-0.0051(5)
C5	0.0281(7)	0.0255(7)	0.0202(6)	0.0011(5)	0.0026(5)	-0.0106(5)
C6	0.0353(8)	0.0200(7)	0.0227(7)	0.0014(5)	-0.0024(6)	-0.0076(6)
C7	0.0321(7)	0.0180(6)	0.0264(7)	-0.0005(5)	-0.0018(6)	0.0037(6)
C8	0.0220(6)	0.0236(7)	0.0218(6)	-0.0012(5)	0.0004(5)	0.0020(5)
C9	0.0173(5)	0.0203(6)	0.0165(5)	-0.0002(4)	0.0019(4)	-0.0016(4)
C10	0.0176(5)	0.0196(6)	0.0147(5)	0.0000(4)	0.0027(4)	-0.0022(4)
C11	0.0176(6)	0.0346(8)	0.0290(7)	0.0017(6)	0.0055(5)	-0.0035(6)
C12	0.0237(7)	0.0256(7)	0.0254(7)	-0.0006(5)	0.0088(5)	0.0036(5)
C13	0.0205(6)	0.0215(6)	0.0185(6)	0.0003(5)	0.0052(5)	-0.0018(5)
C14	0.0202(6)	0.0216(6)	0.0189(6)	0.0010(5)	0.0043(5)	-0.0006(5)
C15	0.0244(6)	0.0176(6)	0.0230(6)	0.0022(5)	0.0065(5)	-0.0016(5)
C16	0.0220(6)	0.0233(6)	0.0232(6)	-0.0019(5)	0.0058(5)	-0.0028(5)
C17	0.0233(6)	0.0206(6)	0.0230(6)	-0.0023(5)	0.0078(5)	-0.0040(5)
C18	0.0240(6)	0.0166(6)	0.0233(6)	-0.0003(5)	0.0096(5)	0.0000(5)
Cr1	0.01782(12)	0.01513(12)	0.01768(12)	-0.00044(7)	0.00628(8)	-0.00186(7)
N1	0.0256(6)	0.0205(6)	0.0192(5)	0.0007(4)	0.0038(4)	-0.0049(4)
O1	0.0330(6)	0.0201(5)	0.0333(6)	0.0010(4)	0.0041(4)	-0.0055(4)
O2	0.0348(6)	0.0294(6)	0.0305(5)	0.0005(4)	0.0166(5)	0.0047(4)
O3	0.0301(6)	0.0437(7)	0.0341(6)	-0.0057(5)	0.0173(5)	-0.0012(5)
O4	0.0293(5)	0.0356(6)	0.0272(5)	0.0023(4)	0.0022(4)	-0.0007(5)
O5	0.0280(5)	0.0281(5)	0.0263(5)	0.0001(4)	0.0031(4)	0.0038(4)

Table S3.6. Torsion angles [°] for **7**.

C9-C1-C2-N1	178.12(11)	C11-C1-C2-N1	-0.7(2)
C9-C1-C2-C3	-0.84(15)	C11-C1-C2-C3	-179.72(12)
N1-C2-C3-C10	-177.54(11)	C1-C2-C3-C10	1.42(15)
N1-C2-C3-C12	1.3(2)	C1-C2-C3-C12	-179.70(12)
C10-C4-C5-C6	-2.2(2)	C4-C5-C6-C7	0.0(3)
C5-C6-C7-C8	2.2(3)	C6-C7-C8-C9	-0.4(3)
C7-C8-C9-C1	177.29(14)	C7-C8-C9-C10	-2.9(2)
C2-C1-C9-C8	179.81(12)	C11-C1-C9-C8	-1.4(2)
C2-C1-C9-C10	-0.07(14)	C11-C1-C9-C10	178.75(13)
C5-C4-C10-C3	-177.46(13)	C5-C4-C10-C9	0.5(2)
C2-C3-C10-C4	176.97(12)	C12-C3-C10-C4	-1.9(2)
C2-C3-C10-C9	-1.38(14)	C12-C3-C10-C9	179.77(12)
C8-C9-C10-C4	2.8(2)	C1-C9-C10-C4	-177.36(12)
C8-C9-C10-C3	-178.95(13)	C1-C9-C10-C3	0.92(14)

A4. X-ray Crystallographic Characterization of 8.

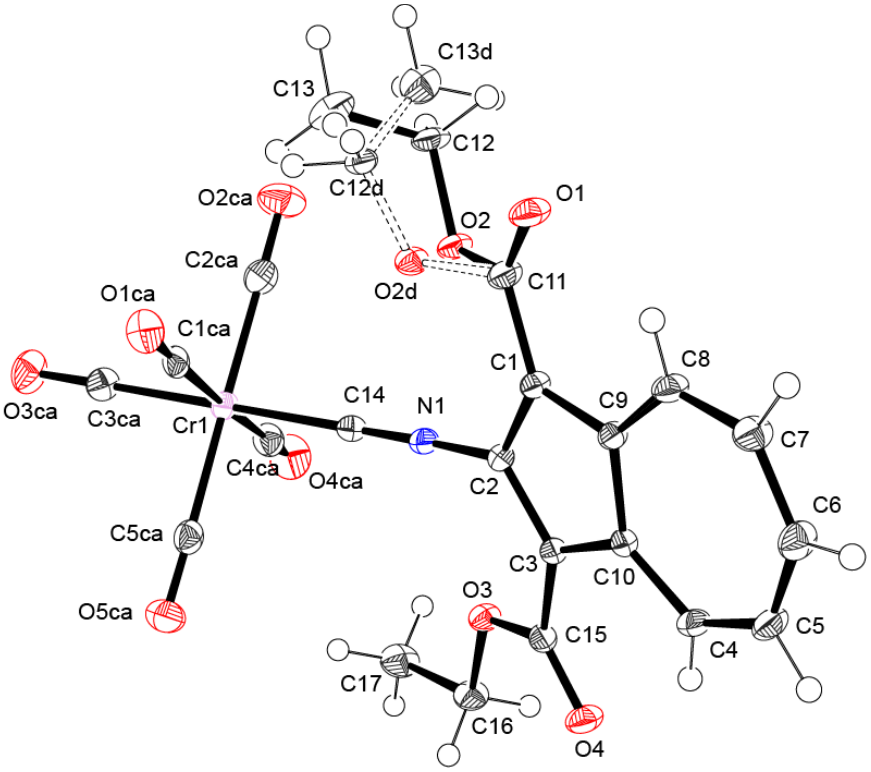

Figure S6. Solid-state structure of **8** showing a minor disorder of one of the ethoxy groups over two positions. All thermal ellipsoids are drawn at the 50% probability level.

Comments

There is a minor disorder in one of the ethoxy-groups, which was successfully modeled over two positions. The H-atoms in that Et-group were placed in geometrically perfect positions according to the hybridization of hosting carbon atoms. However, all other H-atoms were objectively found on the electron difference map and were properly refined.

Table S4.1. Crystal data and structure refinement for **8**.

Chemical formula	$C_{22}H_{15}CrNO_9$	
Formula weight	489.35 g/mol	
Temperature	100(2) K	
Wavelength	0.71073 Å	
Crystal size	0.182 x 0.200 x 0.425 mm	
Crystal habit	clear intense red-pink block	
Crystal system	monoclinic	
Space group	C 1 2/c 1	
Unit cell dimensions	a = 23.9424(14) Å	α = 90°
	b = 15.9966(9) Å	β = 116.6330(10)°
	c = 12.3639(7) Å	γ = 90°
Volume	4232.9(4) $Å^3$	
Z	8	
Density (calculated)	1.536 g/cm^3	
Absorption coefficient	0.596 mm^{-1}	
F(000)	2000	

Theta range for data collection	1.59 to 27.88°
Index ranges	-31<=h<=31, -21<=k<=21, -16<=l<=16
Reflections collected	27455
Independent reflections	5047 [R(int) = 0.0337]
Coverage of independent reflections	99.9%
Absorption correction	multi-scan
Max. and min. transmission	0.7465 and 0.6942
Structure solution technique	direct methods
Structure solution program	SHELXS-1013 (Sheldrick, 2013)
Refinement method	Full-matrix least-squares on F^2
Refinement program	SHELXL-2014/7 (Sheldrick, 2014)
Function minimized	$\Sigma w(F_o^2 - F_c^2)^2$
Data / restraints / parameters	5047 / 0 / 356
Goodness-of-fit on F^2	0.960
Δ/σ_{max}	0.001

Final R indices	4158 data; I>2σ(I)	R1 = 0.0343, wR2 = 0.0818
	all data	R1 = 0.0425, wR2 = 0.0864
Weighting scheme	$w=1/[\sigma^2(F_o^2)+(0.0393P)^2+6.0156P]$<br>where $P=(F_o^2+2F_c^2)/3$	
Largest diff. peak and hole	0.427 and -0.285 eÅ^{-3}	
R.M.S. deviation from mean	0.061 eÅ^{-3}	

Table S4.2. Atomic coordinates and equivalent isotropic displacement parameters ($Å^2$) for **8**. U(eq) is defined as one third of the trace of the orthogonalized U_{ij} tensor.

	**x/a**	**y/b**	**z/c**	**U(eq)**
C1	0.06343(7)	0.18094(9)	0.38736(13)	0.0148(3)
C2	0.08381(7)	0.25523(9)	0.35488(13)	0.0139(3)
C3	0.06065(7)	0.32635(9)	0.38935(13)	0.0138(3)
C4	0.99551(7)	0.34760(10)	0.49878(14)	0.0173(3)
C5	0.96199(8)	0.32400(10)	0.56114(15)	0.0209(3)
C6	0.94902(8)	0.24444(10)	0.58851(17)	0.0234(4)
C7	0.96573(8)	0.16719(10)	0.56052(16)	0.0216(3)
C8	0.00025(8)	0.14981(10)	0.49788(15)	0.0182(3)
C9	0.02794(7)	0.20498(9)	0.44864(13)	0.0146(3)
C10	0.02588(7)	0.29694(9)	0.44917(13)	0.0138(3)
C11	0.07320(8)	0.09440(10)	0.35894(15)	0.0191(3)
C12	0.09952(10)	0.00880(14)	0.23051(19)	0.0213(4)
C13	0.16067(10)	0.96801(14)	0.31219(19)	0.0292(5)
C14	0.16641(7)	0.25573(9)	0.27997(14)	0.0158(3)
C15	0.06894(7)	0.41452(9)	0.36566(13)	0.0155(3)
C16	0.10790(9)	0.50802(10)	0.26729(17)	0.0215(3)
C17	0.14668(10)	0.50231(11)	0.19988(18)	0.0272(4)
C1CA	0.27266(7)	0.17004(10)	0.39126(14)	0.0184(3)
C2CA	0.20725(8)	0.15130(11)	0.15437(15)	0.0230(3)
C3CA	0.31881(8)	0.23163(10)	0.24900(15)	0.0211(3)
C4CA	0.21281(8)	0.31624(11)	0.11838(16)	0.0237(4)
C5CA	0.27767(8)	0.33962(11)	0.36151(15)	0.0211(3)
Cr1	0.24283(2)	0.24525(2)	0.25756(2)	0.01583(8)
N1	0.12478(6)	0.25782(7)	0.30426(12)	0.0160(3)
O1	0.05982(6)	0.03255(7)	0.39915(11)	0.0236(3)
O3	0.09710(5)	0.42262(6)	0.29419(10)	0.0173(2)
O4	0.05165(6)	0.47396(7)	0.40431(11)	0.0227(3)
O1CA	0.29014(6)	0.12320(8)	0.46889(11)	0.0270(3)
O2CA	0.18648(7)	0.09420(8)	0.09492(12)	0.0369(3)

	x/a	y/b	z/c	U(eq)
O3CA	0.36589(6)	0.22279(9)	0.24666(12)	0.0321(3)
O4CA	0.19681(7)	0.35692(9)	0.03417(12)	0.0367(3)
O5CA	0.29935(6)	0.39594(8)	0.42180(12)	0.0312(3)
O2	0.09414(8)	0.09139(8)	0.27427(15)	0.0180(3)
O1D	0.1234(6)	0.0922(6)	0.3270(12)	0.0173(2)
C12D	0.1468(7)	0.0092(9)	0.3191(12)	0.0173(3)
C13D	0.1017(8)	0.9652(12)	0.2136(15)	0.027(4)

Table S4.3. Bond lengths [Å] for **8**.

C1-C2	1.410(2)	C1-C9	1.422(2)
C1-C11	1.472(2)	C2-N1	1.381(2)
C2-C3	1.413(2)	C3-C10	1.419(2)
C3-C15	1.472(2)	C4-C5	1.391(2)
C4-C10	1.400(2)	C4-H4	0.851(19)
C5-C6	1.387(2)	C5-H5	0.93(2)
C6-C7	1.390(2)	C6-H6	0.91(2)
C7-C8	1.391(2)	C7-H7	0.93(2)
C8-C9	1.397(2)	C8-H8	0.869(19)
C9-C10	1.472(2)	C11-O1	1.2130(19)
C11-O2	1.349(2)	C12-O2	1.455(3)
C12-C13	1.504(3)	C12-H12A	0.99
C12-H12B	0.99	C13-H13A	0.98
C13-H13B	0.98	C13-H13C	0.98
C14-N1	1.163(2)	C14-Cr1	1.9767(16)
C15-O4	1.2170(18)	C15-O3	1.3366(18)
C16-O3	1.4563(18)	C16-C17	1.503(2)
C16-H16A	0.93(2)	C16-H16B	1.00(2)
C17-H17A	0.95(2)	C17-H17B	1.00(2)
C17-H17C	0.96(2)	C1CA-O1CA	1.139(2)
C1CA-Cr1	1.9057(17)	C2CA-O2CA	1.138(2)
C2CA-Cr1	1.9073(17)	C3CA-O3CA	1.149(2)
C3CA-Cr1	1.8814(17)	C4CA-O4CA	1.139(2)
C4CA-Cr1	1.9127(18)	C5CA-O5CA	1.136(2)
C5CA-Cr1	1.9146(17)	O1D-C12D	1.461(18)
C12D-C13D	1.45(2)	C12D-H12C	0.99
C12D-H12D	0.99	C13D-H13D	0.98
C13D-H13E	0.98	C13D-H13F	0.98

Table S4.4. Bond angles [°] for **8.**

C2-C1-C9	106.84(13)	C2-C1-C11	127.75(14)
C9-C1-C11	125.33(14)	N1-C2-C1	124.15(13)
N1-C2-C3	124.57(13)	C1-C2-C3	111.11(13)
C2-C3-C10	106.95(13)	C2-C3-C15	127.27(14)
C10-C3-C15	125.76(13)	C5-C4-C10	128.86(15)
C5-C4-H4	115.9(12)	C10-C4-H4	115.2(12)
C6-C5-C4	129.19(15)	C6-C5-H5	114.0(13)
C4-C5-H5	116.8(13)	C5-C6-C7	129.34(16)
C5-C6-H6	116.5(11)	C7-C6-H6	114.1(11)
C6-C7-C8	128.75(15)	C6-C7-H7	115.2(13)
C8-C7-H7	116.0(13)	C7-C8-C9	129.28(15)
C7-C8-H8	117.0(11)	C9-C8-H8	113.7(11)
C8-C9-C1	125.12(14)	C8-C9-C10	127.31(13)
C1-C9-C10	107.57(12)	C4-C10-C3	125.28(14)
C4-C10-C9	127.24(14)	C3-C10-C9	107.48(12)
O1-C11-O2	123.17(14)	O1-C11-C1	124.72(14)
O2-C11-C1	111.89(13)	O2-C12-C13	111.47(19)
O2-C12-H12A	109.3	C13-C12-H12A	109.3
O2-C12-H12B	109.3	C13-C12-H12B	109.3
H12A-C12-H12B	108.0	C12-C13-H13A	109.5
C12-C13-H13B	109.5	H13A-C13-H13B	109.5
C12-C13-H13C	109.5	H13A-C13-H13C	109.5
H13B-C13-H13C	109.5	N1-C14-Cr1	173.09(14)
O4-C15-O3	123.04(14)	O4-C15-C3	124.79(14)
O3-C15-C3	112.17(13)	O3-C16-C17	106.66(13)
O3-C16-H16A	107.3(12)	C17-C16-H16A	111.6(12)
O3-C16-H16B	109.3(12)	C17-C16-H16B	111.3(12)
H16A-C16-H16B	110.5(16)	C16-C17-H17A	109.6(12)
C16-C17-H17B	109.7(12)	H17A-C17-H17B	111.8(17)
C16-C17-H17C	109.4(13)	H17A-C17-H17C	105.5(17)
H17B-C17-H17C	110.8(18)	O1CA-C1CA-Cr1	178.01(14)
O2CA-C2CA-Cr1	178.55(15)	O3CA-C3CA-Cr1	178.36(16)

O4CA-C4CA-Cr1	176.98(16)	O5CA-C5CA-Cr1	178.20(15)
C3CA-Cr1-C2CA	91.40(7)	C3CA-Cr1-C1CA	88.85(7)
C2CA-Cr1-C1CA	87.94(7)	C3CA-Cr1-C4CA	90.06(7)
C2CA-Cr1-C4CA	89.48(7)	C1CA-Cr1-C4CA	177.18(7)
C3CA-Cr1-C5CA	89.30(7)	C2CA-Cr1-C5CA	179.30(7)
C1CA-Cr1-C5CA	91.97(7)	C4CA-Cr1-C5CA	90.61(7)
C3CA-Cr1-C14	175.34(7)	C2CA-Cr1-C14	89.80(7)
C1CA-Cr1-C14	86.70(6)	C4CA-Cr1-C14	94.45(7)
C5CA-Cr1-C14	89.49(6)	C14-N1-C2	168.91(15)
C15-O3-C16	115.82(12)	C11-O2-C12	116.52(14)
C13D-C12D-O1D	110.4(15)	C13D-C12D-H12C	109.6
O1D-C12D-H12C	109.6	C13D-C12D-H12D	109.6
O1D-C12D-H12D	109.6	H12C-C12D-H12D	108.1
C12D-C13D-H13D	109.5	C12D-C13D-H13E	109.5
H13D-C13D-H13E	109.5	C12D-C13D-H13F	109.5
H13D-C13D-H13F	109.5	H13E-C13D-H13F	109.5

Table S4.5. Anisotropic displacement parameters ($Å^2$) for **8**. The anisotropic displacement factor exponent takes the form: $-2\pi^2[h^2a^{*2}U_{11} + ... + 2 h k a^* b^* U_{12}]$.

	**U11**	**U22**	**U33**	**U23**	**U13**	**U12**
C1	0.0167(7)	0.0124(7)	0.0163(7)	0.0002(5)	0.0083(6)	0.0013(5)
C2	0.0136(7)	0.0141(7)	0.0150(7)	-0.0009(5)	0.0071(6)	0.0005(5)
C3	0.0131(7)	0.0125(7)	0.0152(7)	-0.0003(5)	0.0058(6)	0.0002(5)
C4	0.0200(8)	0.0116(7)	0.0212(8)	-0.0010(6)	0.0099(6)	0.0010(6)
C5	0.0244(8)	0.0175(8)	0.0266(8)	-0.0021(6)	0.0167(7)	0.0034(6)
C6	0.0280(9)	0.0231(9)	0.0287(9)	0.0005(7)	0.0212(8)	0.0005(7)
C7	0.0285(9)	0.0161(8)	0.0279(9)	0.0018(6)	0.0194(8)	-0.0020(7)
C8	0.0234(8)	0.0115(7)	0.0225(8)	0.0004(6)	0.0126(7)	0.0012(6)
C9	0.0146(7)	0.0136(7)	0.0151(7)	-0.0006(5)	0.0064(6)	0.0012(5)
C10	0.0127(7)	0.0138(7)	0.0142(7)	-0.0004(5)	0.0054(6)	-0.0003(5)
C11	0.0234(8)	0.0139(7)	0.0254(8)	-0.0011(6)	0.0157(7)	0.0010(6)
C12	0.0303(11)	0.0118(10)	0.0268(11)	-0.0055(8)	0.0172(9)	-0.0001(9)
C13	0.0394(12)	0.0239(11)	0.0282(11)	0.0025(8)	0.0187(10)	0.0115(9)
C14	0.0176(7)	0.0132(7)	0.0166(7)	-0.0008(6)	0.0078(6)	-0.0009(6)
C15	0.0145(7)	0.0141(7)	0.0166(7)	0.0007(6)	0.0059(6)	0.0000(5)
C16	0.0267(9)	0.0118(7)	0.0293(9)	0.0034(6)	0.0155(8)	-0.0017(6)
C17	0.0347(11)	0.0206(9)	0.0333(10)	0.0009(8)	0.0213(9)	-0.0059(7)
C1CA	0.0138(7)	0.0228(8)	0.0213(8)	-0.0046(6)	0.0104(6)	-0.0021(6)
C2CA	0.0246(9)	0.0254(9)	0.0223(8)	0.0002(7)	0.0135(7)	0.0019(7)
C3CA	0.0238(8)	0.0223(8)	0.0219(8)	0.0011(6)	0.0145(7)	-0.0002(6)
C4CA	0.0214(8)	0.0256(9)	0.0258(9)	-0.0016(7)	0.0120(7)	-0.0025(7)
C5CA	0.0182(8)	0.0234(8)	0.0251(8)	0.0007(7)	0.0128(7)	0.0005(6)
Cr1	0.01611(13)	0.01717(14)	0.01764(14)	-0.00089(9)	0.01062(10)	-0.00037(9)
N1	0.0173(6)	0.0124(6)	0.0192(6)	-0.0011(5)	0.0091(5)	-0.0004(5)
O1	0.0333(7)	0.0120(5)	0.0357(7)	-0.0002(5)	0.0245(6)	0.0000(5)
O3	0.0221(6)	0.0120(5)	0.0218(6)	0.0019(4)	0.0134(5)	-0.0004(4)
O4	0.0301(7)	0.0126(5)	0.0328(7)	-0.0018(5)	0.0208(6)	-0.0001(5)
O1CA	0.0254(6)	0.0321(7)	0.0224(6)	0.0048(5)	0.0098(5)	0.0004(5)
O2CA	0.0476(9)	0.0315(7)	0.0333(7)	-0.0133(6)	0.0197(7)	-0.0079(6)

	U_{11}	U_{22}	U_{33}	U_{23}	U_{13}	U_{12}
O3CA	0.0278(7)	0.0382(7)	0.0409(8)	0.0037(6)	0.0248(6)	0.0027(6)
O4CA	0.0390(8)	0.0390(8)	0.0301(7)	0.0125(6)	0.0139(6)	0.0019(6)
O5CA	0.0304(7)	0.0270(7)	0.0374(7)	-0.0103(6)	0.0164(6)	-0.0069(5)
O2	0.0247(9)	0.0128(6)	0.0213(8)	-0.0028(5)	0.0146(8)	0.0011(5)
O1D	0.0221(6)	0.0120(5)	0.0218(6)	0.0019(4)	0.0134(5)	-0.0004(4)
C12D	0.0200(8)	0.0116(7)	0.0212(8)	-0.0010(6)	0.0099(6)	0.0010(6)
C13D	0.033(9)	0.024(9)	0.030(8)	0.005(7)	0.018(7)	0.003(7)

Table S4.6. Torsion angles [°] for **8**.

C9_a-C1_a-C2_a-N1	173.35(14)	C11-C1-C2-N1	-9.7(3)
C9-C1-C2-C3	-2.17(18)	C11-C1-C2-C3	174.74(15)
N1-C2-C3-C10	-173.68(14)	C1-C2-C3-C10	1.81(18)
N1-C2-C3-C15	7.9(2)	C1-C2-C3-C15	-176.62(14)
C10-C4-C5-C6	-1.3(3)	C4-C5-C6-C7	-0.4(3)
C5-C6-C7-C8	0.3(3)	C6-C7-C8-C9	1.2(3)
C7-C8-C9-C1	178.94(17)	C7-C8-C9-C10	-1.5(3)
C2-C1-C9-C8	-178.72(15)	C11-C1-C9-C8	4.3(3)
C2-C1-C9-C10	1.64(17)	C11-C1-C9-C10	-175.37(14)
C5-C4-C10-C3	-178.22(16)	C5-C4-C10-C9	1.9(3)
C2-C3-C10-C4	179.34(14)	C15-C3-C10-C4	-2.2(2)
C2-C3-C10-C9	-0.72(16)	C15-C3-C10-C9	177.74(14)
C8-C9-C10-C4	-0.3(3)	C1-C9-C10-C4	179.36(15)
C8-C9-C10-C3	179.79(15)	C1-C9-C10-C3	-0.57(16)
C2-C1-C11-O1	172.50(16)	C9-C1-C11-O1	-11.1(3)
C2-C1-C11-O2	-12.7(2)	C9-C1-C11-O2	163.65(16)
C2-C3-C15-O4	-174.77(15)	C10-C3-C15-O4	7.1(3)
C2-C3-C15-O3	6.3(2)	C10-C3-C15-O3	-171.86(14)
C1-C2-N1-C14	-69.5(8)	C3-C2-N1-C14	105.4(7)
O4-C15-O3-C16	1.8(2)	C3-C15-O3-C16	-179.26(13)
C17-C16-O3-C15	173.05(14)	O1-C11-O2-C12	0.8(3)
C1-C11-O2-C12	-174.11(16)	C13-C12-O2-C11	-84.4(2)

A5. X-ray Crystallographic Characterization of 10.

Comment

All H-atoms were found on a difference map and refined isotropically.

Table S5.1. Crystal data and structure refinement for **10**.

Chemical formula	$C_{18}H_5CrN_3O_5$	
Formula weight	395.25 g/mol	
Temperature	100(2) K	
Wavelength	0.71073 Å	
Crystal size	0.130 x 0.204 x 0.259 mm	
Crystal habit	clear light orange-red plate	
Crystal system	triclinic	
Space group	P -1	
Unit cell dimensions	a = 6.1716(14) Å	α = 85.048(4)°
	b = 9.244(2) Å	β = 87.266(4)°
	c = 15.070(3) Å	γ = 76.705(4)°
Volume	833.2(3) $Å^3$	
Z	2	
Density (calculated)	1.575 g/cm^3	
Absorption coefficient	0.723 mm^{-1}	
F(000)	396	
Theta range for data collection	1.36 to 25.00°	
Index ranges	-7<=h<=7, -10<=k<=10, -17<=l<=17	
Reflections collected	9244	
Independent reflections	2929 [R(int) = 0.0309]	
Coverage of independent reflections	100.0%	
Absorption correction	multi-scan	
Refinement method	Full-matrix least-squares on F^2	
Refinement program	SHELXL-2014/7 (Sheldrick, 2014)	
Function minimized	$\Sigma w(F_o^2 - F_c^2)^2$	

Data / restraints / parameters	2929 / 0 / 264	
Goodness-of-fit on F^2	1.048	
Final R indices	2283 data; $I>2\sigma(I)$	R1 = 0.0426, wR2 = 0.0995
	all data	R1 = 0.0575, wR2 = 0.1089
Weighting scheme	$w=1/[\sigma^2(F_o^2)+(0.0453P)^2+0.5538P]$ where $P=(F_o^2+2F_c^2)/3$	
Largest diff. peak and hole	0.276 and -0.621 $e\text{Å}^{-3}$	
R.M.S. deviation from mean	0.054 $e\text{Å}^{-3}$	

Table S5.2. Atomic coordinates and equivalent isotropic displacement parameters ($Å^2$) for **10**. U(eq) is defined as one third of the trace of the orthogonalized U_{ij} tensor.

	**x/a**	**y/b**	**z/c**	**U(eq)**
C1	0.6665(4)	0.8092(3)	0.95156(18)	0.0298(6)
C2	0.5635(4)	0.7398(3)	0.89203(17)	0.0307(6)
C3	0.3970(4)	0.6781(3)	0.93681(18)	0.0296(6)
C4	0.2530(4)	0.6621(3)	0.09390(19)	0.0322(6)
C5	0.2478(5)	0.6805(3)	0.1841(2)	0.0397(7)
C6	0.3798(5)	0.7480(3)	0.2312(2)	0.0445(7)
C7	0.5507(5)	0.8181(3)	0.2015(2)	0.0407(7)
C8	0.6321(4)	0.8385(3)	0.11495(19)	0.0343(6)
C9	0.5691(4)	0.7897(3)	0.03759(18)	0.0298(6)
C10	0.3952(4)	0.7069(3)	0.02757(18)	0.0296(6)
C11	0.8369(4)	0.8876(3)	0.92602(18)	0.0319(6)
C12	0.2522(4)	0.6043(3)	0.89722(18)	0.0320(6)
C13	0.7106(4)	0.7416(3)	0.7329(2)	0.0446(7)
C14	0.9682(5)	0.7854(5)	0.4980(2)	0.0664(11)
C15	0.6198(5)	0.6800(4)	0.5692(2)	0.0608(10)
C16	0.6549(5)	0.9535(5)	0.59854(18)	0.0481(9)
C17	0.0626(5)	0.8465(4)	0.6646(2)	0.0593(10)
C18	0.0376(6)	0.5673(5)	0.6331(2)	0.0656(11)
Cr1	0.84482(8)	0.76179(7)	0.61492(3)	0.0542(2)
N1	0.9708(3)	0.9510(2)	0.90140(16)	0.0391(6)
N2	0.1324(4)	0.5446(2)	0.86641(16)	0.0398(6)
N3	0.6218(3)	0.7336(2)	0.80334(15)	0.0359(5)
O1	0.0378(4)	0.8009(3)	0.42652(15)	0.0811(9)
O2	0.4803(4)	0.6345(3)	0.54262(18)	0.0760(8)
O3	0.5370(4)	0.0694(3)	0.58895(13)	0.0543(6)
O4	0.1915(4)	0.8978(3)	0.69434(15)	0.0722(8)
O5	0.1526(5)	0.4519(4)	0.64328(19)	0.0822(9)

Table S5.3. Bond lengths [Å] for **10**.

C1-C2	1.401(4)	C1-C9	1.418(3)
C1-C11	1.429(3)	C2-N3	1.370(3)
C2-C3	1.405(3)	C3-C10	1.415(4)
C3-C12	1.422(4)	C4-C5	1.383(4)
C4-C10	1.398(3)	C4-H4	1.03(3)
C5-C6	1.386(4)	C5-H5	0.88(3)
C6-C7	1.397(4)	C6-H6	0.96(3)
C7-C8	1.388(4)	C7-H7	0.97(3)
C8-C9	1.389(4)	C8-H8	0.93(3)
C9-C10	1.474(3)	C11-N1	1.149(3)
C12-N2	1.154(3)	C13-N3	1.176(3)
C13-Cr1	1.937(3)	C14-O1	1.150(4)
C14-Cr1	1.901(3)	C15-O2	1.143(4)
C15-Cr1	1.908(3)	C16-O3	1.150(4)
C16-Cr1	1.890(4)	C17-O4	1.141(4)
C17-Cr1	1.914(3)	C18-O5	1.140(4)
C18-Cr1	1.920(5)		

Table S5.4. Bond angles [°] for **10.**

C2-C1-C9	108.2(2)	C2-C1-C11	123.9(2)
C9-C1-C11	127.8(2)	N3-C2-C1	123.7(2)
N3-C2-C3	126.1(2)	C1-C2-C3	110.2(2)
C2-C3-C10	107.6(2)	C2-C3-C12	126.0(2)
C10-C3-C12	126.4(2)	C5-C4-C10	128.1(3)
C5-C4-H4	115.0(14)	C10-C4-H4	116.9(14)
C4-C5-C6	129.0(3)	C4-C5-H5	113.3(18)
C6-C5-H5	117.6(18)	C5-C6-C7	130.3(3)
C5-C6-H6	115.7(18)	C7-C6-H6	114.0(18)
C8-C7-C6	128.3(3)	C8-C7-H7	117.2(18)
C6-C7-H7	114.4(18)	C9-C8-C7	128.4(2)
C9-C8-H8	115.5(16)	C7-C8-H8	116.1(16)
C8-C9-C1	125.4(2)	C8-C9-C10	128.1(2)
C1-C9-C10	106.5(2)	C4-C10-C3	124.9(2)
C4-C10-C9	127.7(3)	C3-C10-C9	107.5(2)
N1-C11-C1	176.8(3)	N2-C12-C3	178.8(3)
N3-C13-Cr1	177.3(3)	O1-C14-Cr1	178.4(3)
O2-C15-Cr1	178.0(3)	O3-C16-Cr1	179.1(3)
O4-C17-Cr1	179.6(4)	O5-C18-Cr1	179.6(3)
C16-Cr1-C14	90.08(15)	C16-Cr1-C15	89.00(15)
C14-Cr1-C15	90.25(13)	C16-Cr1-C17	89.94(14)
C14-Cr1-C17	91.75(13)	C15-Cr1-C17	177.74(13)
C16-Cr1-C18	179.32(14)	C14-Cr1-C18	90.53(16)
C15-Cr1-C18	91.30(16)	C17-Cr1-C18	89.73(16)
C16-Cr1-C13	88.43(13)	C14-Cr1-C13	178.27(15)
C15-Cr1-C13	88.86(12)	C17-Cr1-C13	89.12(12)
C18-Cr1-C13	90.97(13)	C13-N3-C2	164.8(3)

Table S5.5. Anisotropic displacement parameters (Å^2) for **10**. The anisotropic displacement factor exponent takes the form: $-2\pi^2[h^2a^{*2}U_{11} + ... + 2 h k a^* b^* U_{12}]$.

	**U11**	**U22**	**U33**	**U23**	**U13**	**U12**
C1	0.0164(12)	0.0245(13)	0.0463(15)	0.0047(11)	0.0096(11)	-0.0050(10)
C2	0.0209(12)	0.0257(13)	0.0413(15)	0.0069(11)	0.0096(11)	-0.0026(10)
C3	0.0201(12)	0.0207(12)	0.0456(16)	0.0050(11)	0.0075(11)	-0.0041(10)
C4	0.0221(13)	0.0231(13)	0.0499(17)	0.0025(11)	0.0116(11)	-0.0063(11)
C5	0.0370(16)	0.0368(16)	0.0474(18)	0.0000(13)	0.0185(13)	-0.0180(13)
C6	0.0453(17)	0.0442(17)	0.0453(18)	-0.0028(14)	0.0193(14)	-0.0169(14)
C7	0.0347(15)	0.0378(16)	0.0513(18)	-0.0033(13)	0.0113(13)	-0.0145(13)
C8	0.0239(13)	0.0249(13)	0.0537(18)	-0.0005(12)	0.0124(12)	-0.0080(11)
C9	0.0198(12)	0.0202(12)	0.0464(16)	0.0036(11)	0.0103(11)	-0.0031(10)
C10	0.0186(12)	0.0189(12)	0.0474(16)	0.0048(11)	0.0112(11)	-0.0015(10)
C11	0.0220(12)	0.0217(13)	0.0478(16)	0.0052(11)	0.0091(11)	-0.0014(11)
C12	0.0220(13)	0.0275(13)	0.0423(15)	0.0093(11)	0.0109(11)	-0.0040(11)
C13	0.0261(14)	0.064(2)	0.0493(18)	0.0070(15)	0.0021(13)	-0.0257(14)
C14	0.0468(19)	0.118(3)	0.052(2)	-0.004(2)	0.0081(16)	-0.057(2)
C15	0.0473(19)	0.095(3)	0.054(2)	-0.0178(18)	0.0258(16)	-0.045(2)
C16	0.0404(18)	0.097(3)	0.0220(15)	0.0028(16)	-0.0045(13)	-0.0480(19)
C17	0.0373(17)	0.109(3)	0.0390(17)	0.0151(17)	0.0046(14)	-0.0410(19)
C18	0.040(2)	0.107(3)	0.058(2)	-0.006(2)	0.0261(17)	-0.039(2)
Cr1	0.0368(3)	0.0976(5)	0.0398(3)	0.0019(3)	0.0093(2)	-0.0440(3)
N1	0.0262(11)	0.0321(12)	0.0573(15)	0.0055(11)	0.0128(10)	-0.0094(10)
N2	0.0285(12)	0.0338(13)	0.0565(15)	0.0025(11)	0.0096(11)	-0.0098(11)
N3	0.0234(11)	0.0428(13)	0.0422(14)	0.0064(10)	0.0064(10)	-0.0143(10)
O1	0.0651(16)	0.157(3)	0.0439(14)	-0.0097(15)	0.0212(12)	-0.0753(17)
O2	0.0496(14)	0.108(2)	0.0900(19)	-0.0392(16)	0.0269(13)	-0.0535(15)
O3	0.0525(14)	0.0984(19)	0.0265(11)	-0.0012(12)	-0.0036(10)	-0.0475(14)
O4	0.0407(12)	0.137(2)	0.0535(14)	0.0131(14)	-0.0065(11)	-0.0575(15)
O5	0.0589(18)	0.105(2)	0.086(2)	-0.0083(18)	0.0329(15)	-0.0331(17)

Table S5.6. Torsion angles [°] for **10**.

C9-C1-C2-N3	-178.1(2)	C11-C1-C2-N3	3.1(4)
C9-C1-C2-C3	1.4(3)	C11-C1-C2-C3	-177.5(2)
N3-C2-C3-C10	178.6(2)	C1-C2-C3-C10	-0.8(3)
N3-C2-C3-C12	-3.2(4)	C1-C2-C3-C12	177.4(2)
C10-C4-C5-C6	0.3(5)	C4-C5-C6-C7	1.3(5)
C5-C6-C7-C8	0.2(5)	C6-C7-C8-C9	-2.5(5)
C7-C8-C9-C1	-177.2(3)	C7-C8-C9-C10	1.7(4)
C2-C1-C9-C8	177.7(2)	C11-C1-C9-C8	-3.5(4)
C2-C1-C9-C10	-1.3(3)	C11-C1-C9-C10	177.4(2)
C5-C4-C10-C3	177.0(3)	C5-C4-C10-C9	-2.4(4)
C2-C3-C10-C4	-179.6(2)	C12-C3-C10-C4	2.2(4)
C2-C3-C10-C9	0.0(3)	C12-C3-C10-C9	-178.3(2)
C8-C9-C10-C4	1.3(4)	C1-C9-C10-C4	-179.6(2)
C8-C9-C10-C3	-178.2(2)	C1-C9-C10-C3	0.9(2)
C1-C2-N3-C13	8.1(11)	C3-C2-N3-C13	-171.3(9)

B. ^{13}C NMR Studies

B1. δ($^{13}CO_{cis}$) vs. δ(^{13}CN) NMR Inverse-Linear Trend

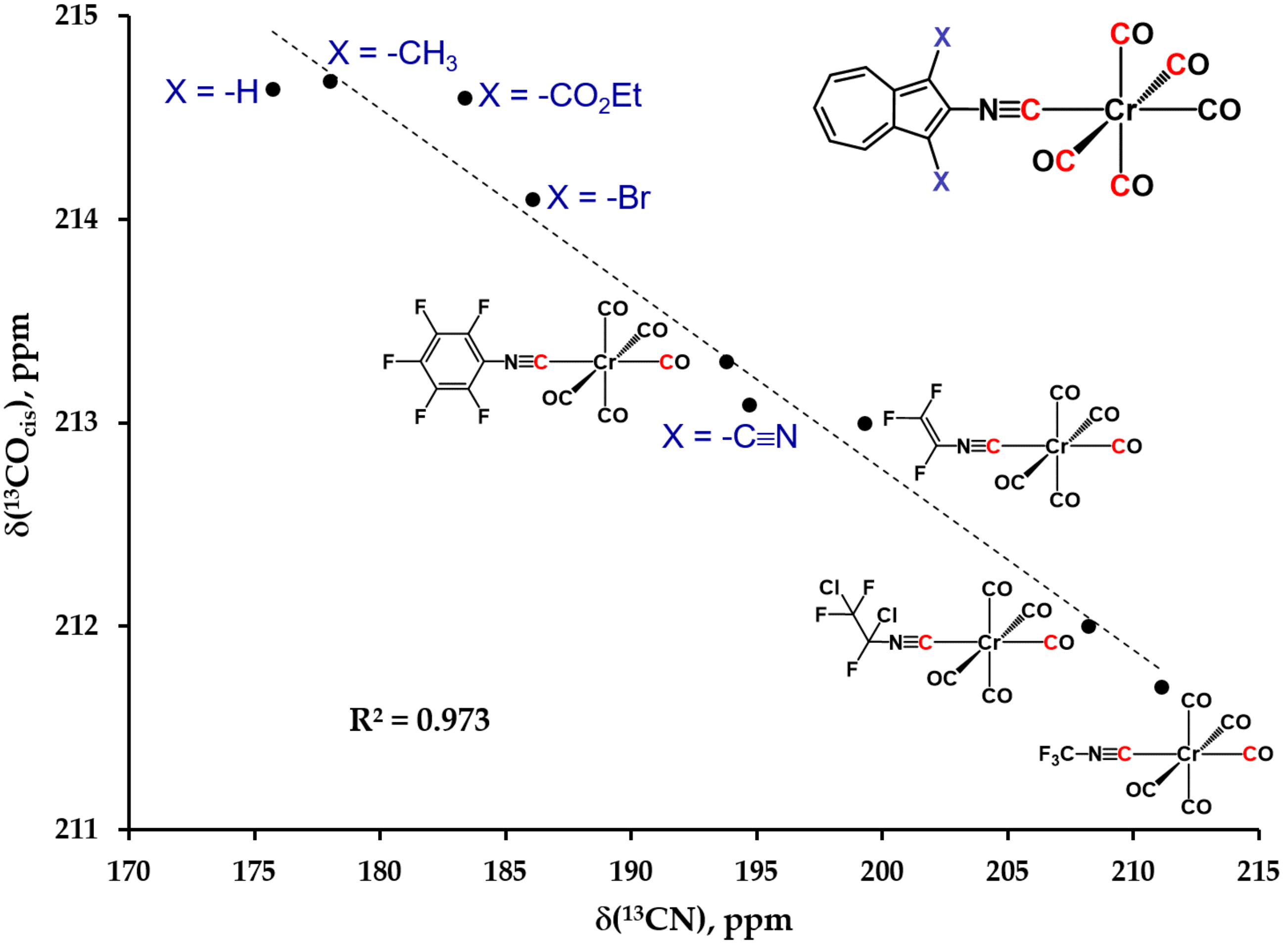

Figure S7. Plot of ^{13}C NMR chemical shifts δ($^{13}CO_{cis}$) vs. δ(^{13}CN) for the series of $(OC)_5Cr(CNR)$ complexes listed in Table 5. All ^{13}C NMR data were collected for solutions in $CDCl_3$.

C. Electrochemical Work

C1. Cyclic Voltammograms

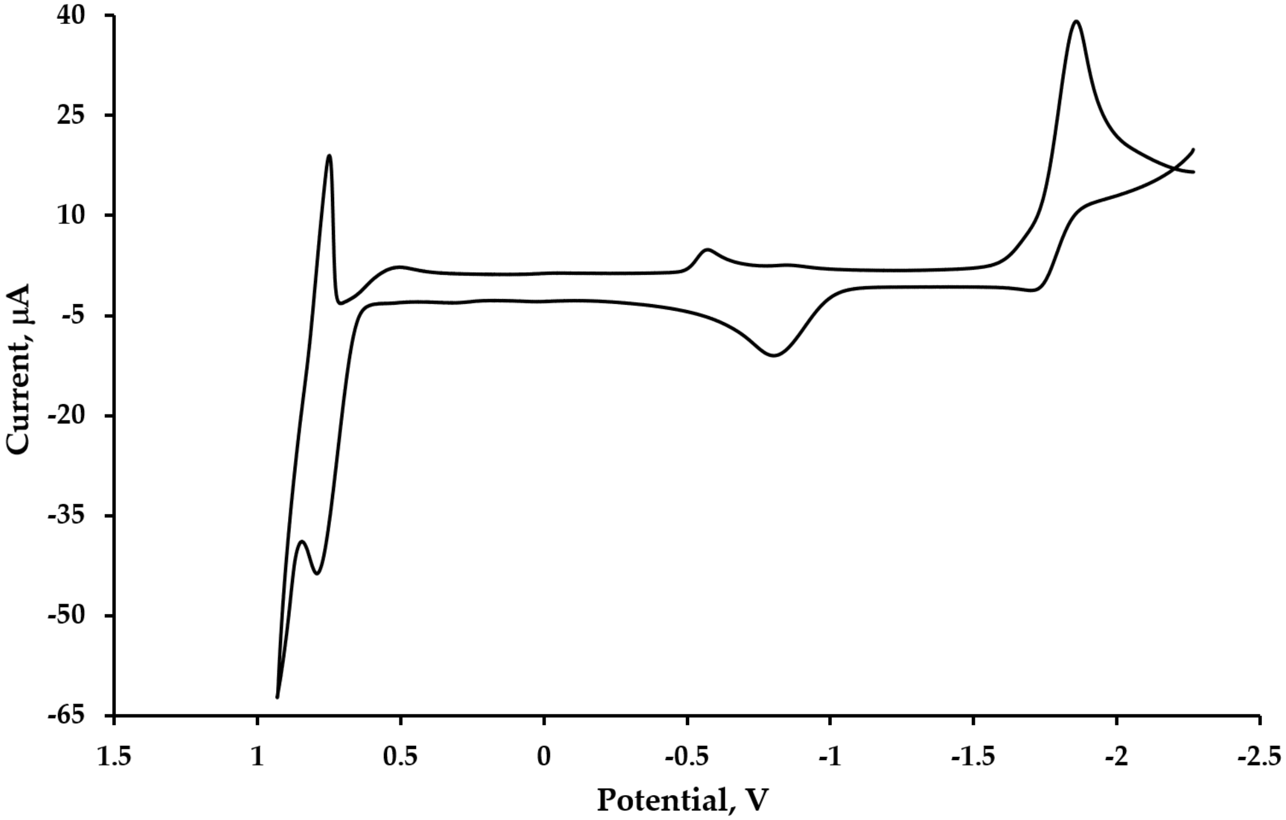

Figure S8. Cyclic voltammogram of *ca*. 0.02 M solution of **6** in 0.1 M [$^{n}Bu_4N$][PF_6]/CH_2Cl_2 vs. external Cp_2Fe/Cp_2Fe^{+} at 25 °C. Scan rate = 100 mV/s.

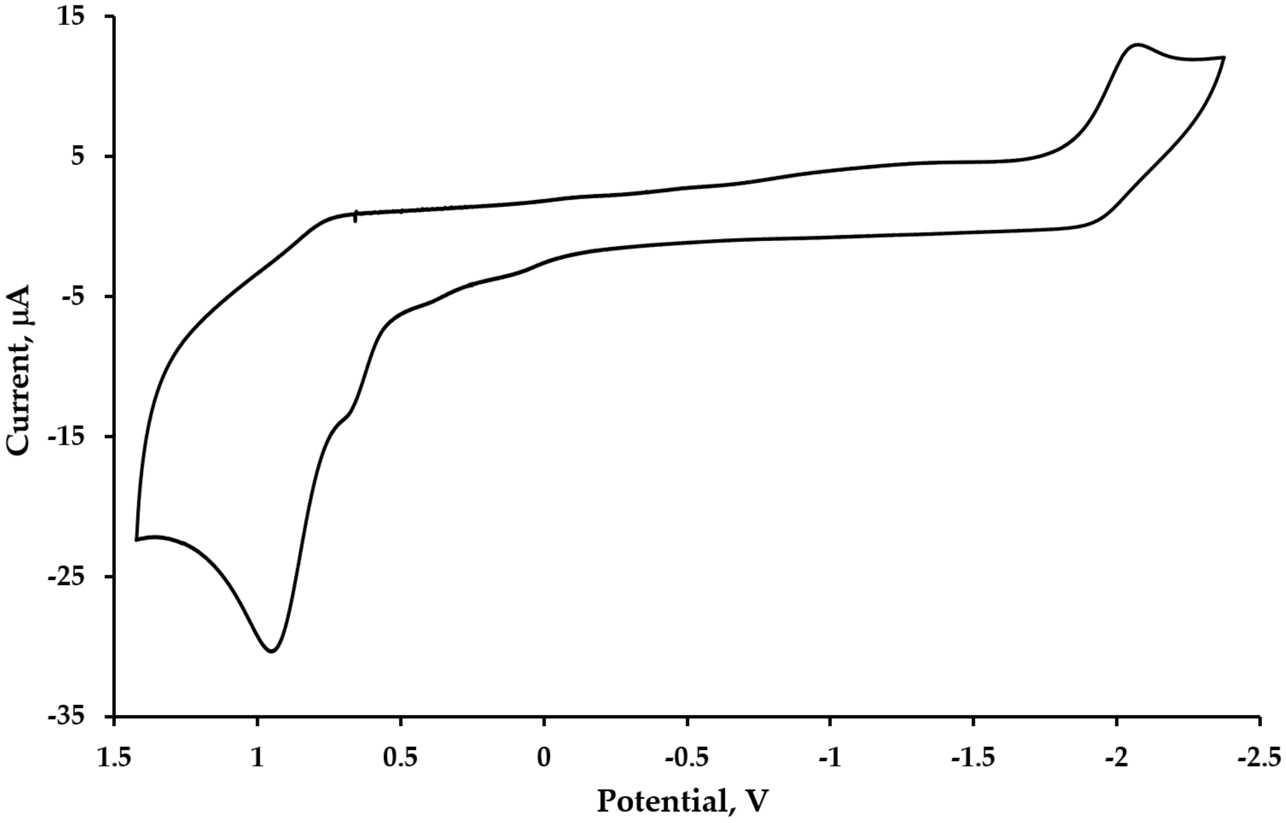

Figure S9. Cyclic voltammogram of *ca*. 0.02 M solution of **7** in 0.1 M $[^nBu_4N][PF_6]/CH_2Cl_2$ vs. external Cp_2Fe/Cp_2Fe^+ at 25 °C. Scan rate = 100 mV/s.

D. Computational Studies

D1. Cartesian Coordinates Pertaining to DFT Calculations

Table S6. Cartesian coordinates (Å) for the optimized geometry of azulene.

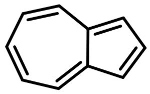

Atom	x	y	z
C	-0.005342	-0.006744	0.004805
H	0.912824	-0.575501	-0.018636
C	-1.304721	-0.531462	0.010988
C	-2.240137	0.510560	0.042209
C	-1.548645	1.731847	0.057129
C	-0.088454	1.393413	0.032518
C	0.981896	2.277209	0.035749
C	0.967989	3.670837	0.062107
C	-0.129649	4.529782	0.092498
C	-1.492866	4.240194	0.104630
C	-2.120772	2.995516	0.088980
H	-3.208031	3.013174	0.103978
H	-2.153818	5.099938	0.130718
H	0.116062	5.587527	0.109802
H	1.939692	4.153262	0.058887
H	1.965417	1.814211	0.013808
H	-3.315254	0.401706	0.053640
H	-1.552031	-1.584480	-0.006665

Table S7. Cartesian coordinates (Å) for the optimized geometry of **10**.

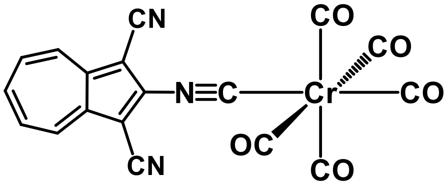

Atom	x	y	z
C	22.147174	15.070753	-2.442840
C	21.511124	14.026948	-1.760307
C	20.194173	13.941864	-1.292231
C	19.167166	14.882945	-1.388457
C	19.200515	16.149379	-1.978155
C	20.337401	16.807112	-2.646316
C	21.627708	16.306067	-2.836365
C	18.099952	17.046537	-2.033162
C	18.524882	18.225217	-2.710001
C	19.890795	18.083020	-3.085368
H	22.133736	13.150199	-1.563871
N	17.756590	19.303470	-2.956242
C	16.811760	16.809538	-1.508967
C	20.666637	19.039542	-3.773447
C	17.078311	20.257479	-3.177296
Cr	15.983516	21.794142	-3.537408
C	15.184377	21.622472	-1.813843
C	17.335548	22.927426	-2.811075
C	14.632531	20.658837	-4.262630
C	16.787366	21.961340	-5.259371
C	14.897665	23.318255	-3.895767
O	14.240483	24.241971	-4.111747
O	14.701084	21.522761	-0.772043
O	13.815228	19.974997	-4.702542
O	18.151267	23.612729	-2.370529
O	17.270088	22.064584	-6.301080
H	23.192947	14.896432	-2.705197
H	19.932583	13.011812	-0.782974
H	18.207137	14.598872	-0.947006
H	22.315003	16.974452	-3.363940
N	21.324545	19.819032	-4.342837
N	15.752949	16.591616	-1.066116
